# Evaluating the potential health and economic impacts of chlamydia vaccination strategies in the United States: a mathematical modeling and cost-effectiveness simulation study

**DOI:** 10.1016/j.lana.2026.101502

**Published:** 2026-05-26

**Authors:** Gregory K. Zane, Dobromir Dimitrov, Carol E. Levin, Christine M. Khosropour, Ann Duerr

**Affiliations:** a Department of Epidemiology, University of Washington, 3980 15th Ave NE, Seattle, WA, 98195, USA; b Vaccine and Infectious Disease Division, Fred Hutch Cancer Center, 1100 Fairview Ave N, Seattle, WA, 98109, USA; c Department of Applied Mathematics, University of Washington, 4182 W Stevens Way NE, Seattle, WA, 98105, USA; d Department of Global Health, University of Washington, 3980 15th Ave NE, Seattle, WA, 98195, USA

**Keywords:** Chlamydia trachomatis, Vaccination, Mathematical model, Cost-effectiveness, Epidemiology, United States

## Abstract

**Background:**

*Chlamydia trachomatis* (chlamydia) is the most commonly reported sexually transmitted infection in the United States (US), causing substantial morbidity and costs despite effective treatment. Vaccination may be a promising prevention strategy, but its national epidemiologic and economic impact remains understudied.

**Methods:**

We developed an age- and sex-structured compartmental model of heterosexual chlamydia transmission among US individuals aged 15–64 years from 2000 to 2075. The model was calibrated to adjusted annual chlamydia cases and validated against national prevalence data. We simulated routine vaccination of adolescent females or both sexes beginning in 2025, with or without a one-time catch-up campaign for unvaccinated females aged 15–24 years in 2035. Outcomes included chlamydia incidence rates, infections and sequelae averted by 2075, and incremental cost-effectiveness ratios through 2050.

**Findings:**

Without vaccination, incidence rates were projected to rise from 16.9 to 18.7 infections per 1000 person-years between 2025 and 2075. Under base-case assumptions (50% coverage, 70% efficacy, 10-year protection), incidence rates declined to 4.0 and 0.4 per 1000 person-years under female-only and sex-neutral vaccination, respectively. By 2075, female-only and sex-neutral vaccination reduced infections by 37.4% and 52.3%, respectively. Catch-up vaccination provided modest additional benefit. All strategies were cost-saving; sex-neutral vaccination yielded 145,943 additional quality-adjusted life years and $354 million in savings versus female-only vaccination by 2050. Results were robust across sensitivity analyses.

**Interpretation:**

Chlamydia vaccination could substantially reduce infections and sequelae in the US and is likely to be cost-saving or highly cost-effective across plausible scenarios.

**Funding:**

This work was primarily supported by the 10.13039/100000002National Institutes of Health (1F31AI181431-01A1).

**Research in context**

**Evidence before this study**

The use of vaccination to achieve population-level declines in infectious disease burden is well-documented. However, in the absence of a viable candidate in late-stage development, the potential epidemiologic and economic impact of chlamydia vaccination remains understudied. We reviewed the literature to identify studies using mathematical modeling and/or economic evaluations to assess the potential benefits of chlamydia vaccination by searching the following terms on PubMed between January 2000 and July 2025 without language restrictions (“Chlamydia” [Mesh] OR “Chlamydia” [tiab]) AND (“Vaccines” [Mesh] OR “Immunization” [Mesh] OR “Vaccination” [Mesh] OR “vaccine∗” [tiab] OR “vaccination” [tiab]) AND (“Models, Theoretical” [Mesh] OR “mathematical” [tiab] OR “compartment∗” [tiab] OR “Epidemiological Models” [Mesh] OR “Cost-Benefit Analysis” [Mesh] OR “Economics, Medical” [Mesh] OR “Costs and Cost Analysis” [Mesh] OR “Cost of Illness” [Mesh] OR cost-effectiveness [tiab]). We identified six relevant studies. Prior modeling studies have provided important insights into the potential population-level impact of chlamydia vaccination. Four focused on changes in disease burden, including one study restricted to pulse vaccination and several using simplified or non-nationally representative populations. Two studies incorporated economic components, though only one conducted a formal cost-effectiveness analysis and focused on individuals under 25 years of age. Across studies, assumptions of coverage, efficacy, and duration of protection varied widely, reflecting uncertainty surrounding potential vaccine characteristics. Collectively, these studies highlighted a need for updated, nationally representative modeling that integrates both epidemiologic and economic outcomes.

**Added value of this study**

We advance existing evidence by presenting the first cost-effectiveness analysis of chlamydia vaccination using an age- and sex-structured dynamic transmission model applied to a nationally representative population in the US. The study assessed both the epidemiologic impact of vaccination and its economic value from a societal perspective, demonstrating that vaccination could be cost saving or highly cost-effective under realistic assumptions. These findings fill a critical gap in the STI prevention literature and offer evidence to guide future vaccine development and policy.

**Implications of all the available evidence**

The substantial burden of chlamydia in the US has prompted growing efforts to identify effective biomedical prevention strategies. Our analysis builds on prior work by demonstrating that chlamydia vaccination, when implemented as a complement to existing screening and treatment programs, could reduce infections and sequelae soon after introduction. Despite initial costs, vaccination may yield cost-saving or cost-effective outcomes under realistic implementation strategies. Future research should evaluate both expanded population-level approaches and targeted vaccination of high-risk groups to capture the full epidemiologic and economic benefits. Such evidence will be critical to advancing clinical development of vaccine candidates.

## Introduction

*Chlamydia trachomatis* (chlamydia) is the most common notifiable sexually transmitted infection (STI) in the United States (US), with approximately 1.6 million cases reported in 2023.^1^ The persistence of chlamydia is multifactorial, driven by stigmatization, fragmented and inequitable access to healthcare services, and high rates of asymptomatic presentation, contributing to missed treatment and ongoing transmission to sexual partners.^1^, ^2^, ^3^ Chlamydia disproportionately affects women and individuals aged ≤25 years and is the most costly non-viral STI in the US.^1^^,^^4^, ^5^, ^6^ Lifetime direct medical and productivity costs of chlamydia infections acquired in 2018 were estimated at ∼$870 million and $560 million, respectively (2025 USD).^5^^,^^6^ Women bear a disproportionate share of these costs due to the high burden of pelvic inflammatory disease, infertility, ectopic pregnancy, and chronic pelvic pain from untreated infections.

Screening remains a widely implemented prevention strategy in the US, with testing recommended for sexually active women under 25, at-risk women 25 and older, pregnant individuals, and men who have sex with men (MSM).^7^ Screening coverage is suboptimal, prompting interest in emerging strategies like doxycycline post-exposure prophylaxis (doxy-PEP).^8^^,^^9^ Concerns of increasing antimicrobial resistance in *Neisseria gonorrhoeae* and the absence of supportive data in heterosexual populations limit broader doxy-PEP implementation.^9^ Consequently, vaccination has emerged as a complementary strategy, with pre-clinical and early-stage clinical trials showing encouraging results.^10^

In the absence of a viable candidate, mathematical modeling remains a critical tool for estimating the potential population-level impacts of vaccination. Previous modeling studies suggest that a partially efficacious vaccine could lead to substantial reductions in chlamydia burden shortly after introduction.^11^, ^12^, ^13^, ^14^, ^15^, ^16^ To date, two studies have evaluated vaccine impact at the population level.^11^^,^^15^ Importantly, the economic value of chlamydia vaccination is largely understudied. A 2015 analysis estimated that vaccinating 14-year-old girls, either alone or with catch-up vaccination for women aged 15–24 years, would be cost-effective.^12^ However, researchers modeled a simplified population of 100,000 individuals aged 15–24 years, only. To our knowledge, no prior work has assessed the cost-effectiveness of female-only, sex-neutral, or catch-up vaccination strategies at the national level, representing an important opportunity to extend the evidence base.

Building upon prior studies, this study aimed to evaluate the potential health and economic impacts of chlamydia vaccination strategies in the US. Specifically, we assessed how different vaccination strategies and characteristics could affect future disease burden and evaluated cost-effectiveness using quality-adjusted life years (QALYs) and incremental cost-effectiveness ratios (ICERs). These findings aim to support future vaccine policy and implementation planning.

## Methods

### Model description

**Fig. 1:**
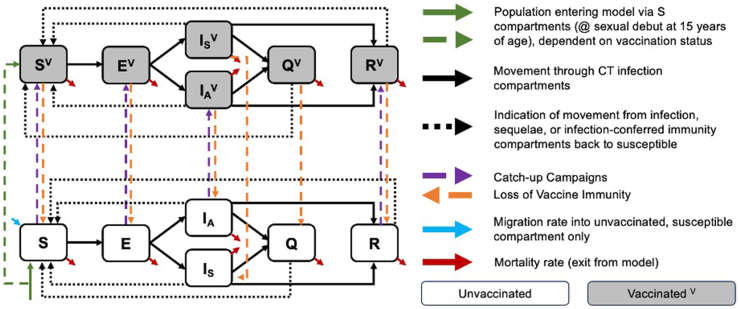
*Chlamydia trachomatis* (CT) transmission model. Simulated population is stratified by CT infection status and progression towards sequelae: susceptible (S), exposed non-infectious (E), infectious symptomatic infection (IS), infectious asymptomatic infection (IA), sequelae-experiencing (Q), or infection-conferred immunity (R). The population is further stratified by age (15–24, 25–39, 40–64 years), biological sex assigned at birth (males, females), and vaccination status (^V^).

An age- and sex-structured deterministic, compartmental mathematical model using ordinary differential equations was constructed to simulate chlamydia transmission among the general population aged 15–64 years in the US from 2000 to 2075 (Fig. 1; Supplementary Methods). The model was structured using a contemporary understanding of disease transmission and natural history, leveraging other published models of STIs.^11^, ^12^, ^13^, ^14^^,^^17^

The simulated population was divided into six groups by biological sex at birth and age: males and females aged 15–24, 25–39, and 40–64 years. Age groups were selected *a priori* to reflect the higher burden of chlamydia among individuals aged 15–24 years compared to those 25 years and older.^18^ Additional stratification for the 25–39 and 40–64 age groups was included to reflect the age-related decline in acquisition risk, influenced by decreasing clinical and behavioral risk factors and the potential protective effects of long-lasting partial immunity to reinfection.^7^^,^^19^ Partial assortative mixing by age was assumed as individuals preferred to form sexual partnerships within the same age group.^20^ Our model does not explicitly account for same-sex transmission, instead focusing on the substantial burden of chlamydia among individuals with female reproductive anatomy who subsequently experience the most severe sequelae following infection.^18^^,^^21^ The simulated population was further stratified by vaccination status and disease stage: susceptible, exposed non-infectious, infectious symptomatic infection, infectious asymptomatic infection, sequelae-experiencing, or infection-conferred immunity. Model calibration was performed using adjusted annual chlamydia cases from the US Centers for Disease Control and Prevention's AtlasPlus application, resulting in 100 parameter sets incorporating parameter uncertainty.^18^ A description of model structure, differential equations, parameterization, calibration, and validation with National Health and Nutrition Examination Survey (NHANES) data can be found in the Supplementary Methods (Supplementary Figures S1–S14; Supplementary Tables S1–S3).^22^

### Vaccination scenarios

The referent scenario applied the calibrated model without vaccination, reflecting the current standard of care in the US, inclusive of screen-and-treat for asymptomatic infections and test-and-treat for symptomatic infections. The impact of vaccination, introduced in 2025, was evaluated through four scenarios. Routine vaccination was assessed as vaccination of 15-year-old females entering the simulated population (Scenario A) or vaccination of 15-year-old females and males entering the simulated population (Scenario B). Vaccination was set to begin at age 15 due to data availability and concerns of parental hesitancy impacting coverage. Within each scenario group, we modeled vaccination coverage increasing linearly from 0% in 2025 to 50% by 2035 and remaining fixed at that level through 2075. A simplified linear scale-up was used to provide a transparent, interpretable implementation scenario given uncertainty in future vaccine uptake. We varied efficacy in reducing the risk of chlamydia acquisition (20%–90%) and duration of vaccine-conferred immunity (0.5–20 years) to assess the potential impact of future vaccines with different characteristics. Additional scenarios with coverage levels of 30% and 70% by 2035 were considered to understand the importance of vaccine uptake on the projected results.

Next, the inclusion of a single catch-up campaign in 2035 among unvaccinated females aged 15–24 years was added to female-only routine vaccination (Scenario C) or sex-neutral routine vaccination (Scenario D). We held routine vaccination coverage constant at 50% by 2035 and assumed that a catch-up campaign in 2035 would reach 5%, 10%, or 15% of unvaccinated females living without symptomatic infection or sequelae. Efficacy and duration of vaccine-conferred immunity were varied in the same manner as Scenarios A/B. An outline of scenarios under base-case assumptions are presented in Table 1. Additional details on vaccination scenarios and vaccine characteristics can be found in the Supplementary Methods (Supplementary Tables S4 and S5).

**Table 1:** Referent and intervention scenarios under base-case assumptions.^a^

Scenario	Description	Target population	Coverage	Start year	Catch-up campaign
Referent	Calibrated model without vaccination; reflects U.S. standard of care (screen-and-treat for asymptomatic, test-and-treat for symptomatic)	N/A	N/A	N/A	No
A	Female-only routine vaccination	15-year-old females	Linear scale-up to 50% by 2035	2025	No
B	Sex-neutral routine vaccination	15-year-old females and males	Linear scale-up to 50% by 2035	2025	No
C	Female-only routine + catch-up	Scenario A + unvaccinated females aged 15–24	50% routine + 10% catch-up (2035)	2025 (routine), 2035 (catch-up)	Yes (females 15–24 years)
D	Sex-neutral routine + catch-up	Scenario B + unvaccinated females aged 15–24	50% routine + 10% catch-up (2035)	2025 (routine), 2035 (catch-up)	Yes (females 15–24 years)

a Base-case assumes 10 years of vaccine-conferred immunity, 70% vaccine efficacy, and 50% routine coverage attained by 2035, with 10% coverage for a catch-up campaign in 2035.

### Epidemiologic outcomes

The effectiveness of vaccination on disease burden across all scenarios was compared to the referent scenario with no vaccination over the 50-year period from 2025 to 2075. Epidemiologic outcomes of interest included 1) the cumulative number of chlamydia infections prevented by 2075, 2) the proportional reduction in infections prevented by 2075, and 3) the reduction in annual incidence rates by 2050 and 2075 among individuals aged 15–64 years. The number of infections prevented per 1000 vaccination doses by 2075 was used to estimate the efficiency of each implementation strategy. Formal definitions of all outcomes of interest are included in Supplementary Table S6. Estimates were reported as the median and interquartile range, when applicable, across all 100 plausible parameter sets from model calibration, with values summarized separately for each combination of efficacy and duration of protection to account for uncertainty within scenarios. The main text presents results from the base-case scenario, assuming 10 years of immunity, 70% efficacy, and 50% coverage by 2035, unless stated otherwise. Finally, to understand the broader impacts of vaccination, we conducted secondary analyses to estimate the cumulative and proportional reductions in sequalae events, including pelvic inflammatory disease, tubal factor infertility, ectopic pregnancy, chronic pelvic pain, and epididymitis, by 2075 under base-case assumptions (Supplementary Methods).

### Costs, health benefits, and economic outcomes

We used ingredient-based methods to estimate the costs of chlamydia prevention and treatment using literature-derived unit costs. Cost components included screening, diagnostic testing and treatment for acute infections, sequelae management, and vaccination, which included the price-per-dose and per-dose implementation costs representing vaccine administration during a clinical visit. To reflect the societal perspective, direct medical costs and productivity losses (indirect costs) were collected separately. Direct medical costs included labor, cost of diagnostic tests, medical supplies, and medications, among other relevant costs. Treatment costs associated with pelvic inflammatory disease incorporated both acute care and the expected value of downstream sequelae. Productivity losses captured the value of time lost due to travel, waiting, and receipt of services.^6^ Rather than modeling each complication separately, we applied average per-case estimates of direct medical costs and productivity losses from published sources that accounted for the likelihood and timing of these outcomes. This approach reflected the full burden of pelvic inflammatory disease without requiring explicit tracking of long-term sequelae within the model. We assumed a base-case price of $100 USD per dose. In the absence of a licensed vaccine, vaccination was assumed to occur during a routine clinical visit with costs consistent with an injectable adolescent vaccine in the US. We also examined a price-per-dose range of $25 to $1000 in sensitivity analyses, informed by the costs of analogous vaccines (e.g., hepatitis B, Mpox, human papillomavirus (HPV)).^23^ For all costs of chlamydia prevention and treatment, we identified a primary estimate with plausible lower and upper bounds for use in sensitivity analyses to account for uncertainty in the published literature. All values were adjusted to 2025 US dollars using the Consumer Price Index.^24^ After adjustment, direct medical costs and productivity losses were combined into total unit costs (Table 2).

**Table 2:** Outline of cost and health utility estimates.

Category	Direct costs^a^^,^^i^ (USD 2025)	Indirect costs^b^^,^^i^ (USD 2025)	Total costs^c^^,^^i^ (2025 USD)	Sources^j^
**A. Costs applied to both sexes**				
Vaccination (Implementation)^d^	$35.17 ($26.38, $43.96)	$35.17 ($15.35, $69.11)	$70.34 ($41.73, $113.07)	S2,S60–65
Vaccination (Price Per Dose)	$ per dose ($25–$1000)	N/A	$ per dose ($25–$1000)	S65
Screening without treatment	$147.00 ($98.00, $196.00)	$35.17 ($15.35, $69.11)	$182.17 ($113.35, $265.11)	S58,S60 SS66–68
**B. Female-Specific costs**				
Treatment of acute infection	$187.00 ($164.00, $210.00)	$68.48 ($29.30, $135.66)	$255.48 ($193.30, $345.66)	S60,S66,S69
Treatment of PID^e^	$3049.26 ($2377.03, $4568.92)	$2265.49 ($853.86, $4690.49)	$5314.75 ($3230.89, $9259.41)	S60,S69
**C. Male-Specific costs**				
Treatment of acute infection	$194.00 ($160.00, $228.00)	$68.48 ($29.30, $135.66)	$262.48 ($189.30, $363.66)	S60,S66,S69
Treatment of epididymitis	$465.96 ($288.28, $643.65)	$740.22 ($279.41, $1532.57)	$1206.18 ($567.69, $2176.22)	S60,S69
**Category**		**Females**	**Males**	**Sources**^j^

**D. Health utilities (QALYs)**^f^				
Acute symptomatic CT infection		0.010 (0.005, 0.015)	0.006 (0.003, 0.009)	S2,S48,S57
Acute asymptomatic CT infection^g^		0	0	S2,S58,S59
Epididymitis		–	0.010 (0.005, 0.015)	S2,S48,S57
PID & Complications^h^		0.4144 (0.2072, 0.6216)	–	S2,S48,S59

Abbreviations: PID, pelvic inflammatory disease; CT, *Chlamydia trachomatis*; QALYs, quality-adjusted life years.

a Direct costs include all medical costs (supplies, diagnostics, provider salaries, etc.) for one unit of the corresponding category.

b Indirect costs represent productivity losses for one unit of the corresponding category, capturing the value of time lost due to travel, waiting, and receipt of CT-related services.

c Total costs include the total direct and indirect costs for one unit of the corresponding category.

d Implementation costs represent per-dose vaccine administration costs (e.g., provider time, clinical visit costs, and supplies) based on published estimates of vaccine administration in U.S. clinical settings. These costs reflect recurrent delivery expenses applied per dose and do not include one-time vaccine introduction costs such as training, program start–up activities, or social mobilization.

e Costs for PID were estimated by combining the probability and discounted cost of each PID-related outcome (outpatient and inpatient treatment, ectopic pregnancy, tubal factor infertility, chronic pelvic pain), using a 3% annual discount rate and assuming the time from infection to PID onset was under one year.

f Health utilities represent average QALYs lost per incident event.

g Asymptomatic CT infection assumed to have no impact on QALYs.

h QALYs lost for PID accounts for the ‘average’ case of PID, based on probability of further development of chronic pelvic pain, ectopic pregnancy, infertility, or no other complications.

i Costs are presented as main unit cost (lower bound, upper bound).

j Source numbers based on references in Supplemental Material.

Unit costs were applied to model-projected numbers of individuals screened, treated, vaccinated, or experiencing sequelae to estimate total costs for each scenario. Costs were assigned to corresponding outflows in the model and estimated annually from 2025 to 2050. To ensure inclusion of diagnostic misclassification and missed treatments, we included model parameters on test sensitivity, specificity, and the probability of treatment following positive test results to ensure appropriate tracking of costs dependent on screening and treatment outcomes. A 3% annual discount rate was applied to future costs, and final cost outcomes were presented in 2025 USD. Additional details on model parameters, sources, and equations can be found in the Supplementary Methods (Supplementary Tables S7 and S8).

Health outcomes were measured in QALYs, a summary measure capturing both life expectancy gains and improvements in health-related quality of life.^25^ In line with previous work on chlamydia vaccination, utility losses were applied to acute symptomatic and asymptomatic infections and associated sequelae (Table 2; Supplementary Table S9).^12^ For pelvic inflammatory disease, the utility weight represented a weighted average based on the probabilities of experiencing downstream sequelae, including tubal factor infertility, ectopic pregnancy, and chronic pelvic pain.^12^^,^^26^ Cumulative QALYs for each intervention scenario were estimated from 2025 to 2050 and discounted at 3% annually. Incremental QALYs gained were calculated as the difference in total QALYs between an intervention and its comparator scenario (see Supplementary Methods and Supplementary Tables S10–S11).

We estimated ICERs, defined as the additional cost per QALY gained when moving from one vaccination strategy to a more extensive strategy. Therefore, ICERs either reflect the incremental cost-effectiveness of introducing vaccination compared to the current standard of care or the incremental cost-effectiveness of expanding vaccination beyond a simpler program. Sequential analysis was conducted separately for female-only (Scenario A to Scenario C) and sex-neutral routine vaccination (Scenario A to Scenario B to Scenario D). We evaluated ICERs over a time horizon from 2025 to 2050 and interpreted ICERs to be very cost-effective if they fell below the willingness-to-pay threshold (WTP) of $50,000 USD per QALY gained ($/QALY).^27^ Although WTP values for the U.S. have been cited up to $150,000 USD, we took a conservative approach to reflect potential bias against sexual and reproductive health services. For simplicity, ICERs were estimated under the base-case scenario, assuming 10 years of vaccine-conferred immunity, 70% efficacy, and 50% routine coverage attained by 2035, with 10% coverage for a catch-up campaign in 2035. Additional details on ICER estimation can be found in the Supplementary Methods.

### Sensitivity analysis

To examine the robustness of ICER findings, we conducted one-way sensitivity analyses by individually varying the price-per-dose, treatment and prevention costs, QALY estimates, and discounting rates using feasible ranges informed by published studies. Model calibration incorporated key epidemiological uncertainties for several parameters, including the probability of symptomatic presentation and likelihood of sequalae development. As such, these parameters were not separately varied in one-way sensitivity analyses. All sensitivity analyses were performed on the base-case scenario (Scenarios A-D: 50% coverage by 2035, 10-year duration of protection, and 70% efficacy; Scenarios C/D: 10% catch-up campaign coverage in 2035). A scenario analysis was also conducted to assess how variation in assumptions of efficacy, duration of protection, and coverage by 2035 influenced ICERs under the base-case scenario (see Supplementary Methods and Supplementary Table S12).

### Software and reporting

The model was developed using R (version 4.4.1). The cost-effectiveness analysis was conducted using R (version 4.4.1) and Microsoft Excel (version 16.99.2). We reported our cost-effectiveness analysis in accordance with the Consolidated Health Economic Evaluation Reporting Standards (CHEERS) 2022 checklist (see Supplementary Materials for the completed checklist).

### Ethics approval

This study used secondary, deidentified data and did not involve human subjects. Institutional review board approval was not required.

### Role of the funding source

The funders had no role in the design, collection, analysis, and interpretation of data, decision to publish, or preparation of the manuscript.

## Results

### Epidemiologic outcomes

**Fig. 2:**
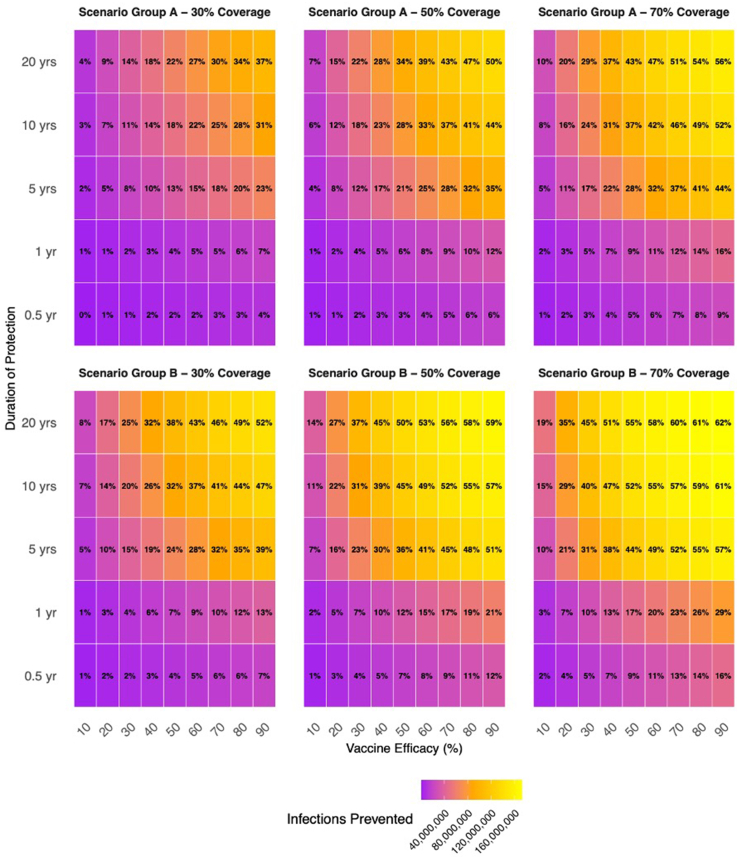
Cumulative CT infections prevented among males and females aged 15–64 years between 2025 and 2075, across combinations of vaccine coverage, efficacy, and duration of protection, for Scenario Groups A and B compared to no vaccination (referent scenario). Heatmap: Cumulative infections prevented represented by colors. Text Values: Relative reduction in CT infections. Scenario A: Vaccinating 15-year-old females entering the model between 2025 and 2075. Scenario B: Vaccinating 15-year-old females and males entering the model between 2025 and 2075.

The model adequately reproduced key epidemiologic patterns of chlamydia in the US during calibration (Supplementary Figures S15 and S16). In the absence of vaccination, 277 million cumulative infections were projected between 2025 and 2075. Vaccinating 15-year-old-females (Scenario A) under the base-case scenario averted 104 million cumulative infections (37.4% reduction) compared to the referent scenario with no vaccination (Fig. 2). The inclusion of vaccinating males (sex-neutral vaccination, Scenario B) contributed to an additional 41 million infections averted over the 50-year period, of which roughly ∼53% were infections among females. Adding a catch-up campaign for unvaccinated females aged 15–24 in 2035 with 10% coverage modestly increased impact, averting an additional 4.7 million infections in Scenario C (female-only; relative to Scenario A) and 2.2 million in Scenario D (sex-neutral; relative to Scenario B). Results from scenario analysis, assuming different vaccine characteristics beyond the base-case, can be found in Supplementary Figures S17 and S18 and Supplementary Tables S13–S16).

**Fig. 3:**
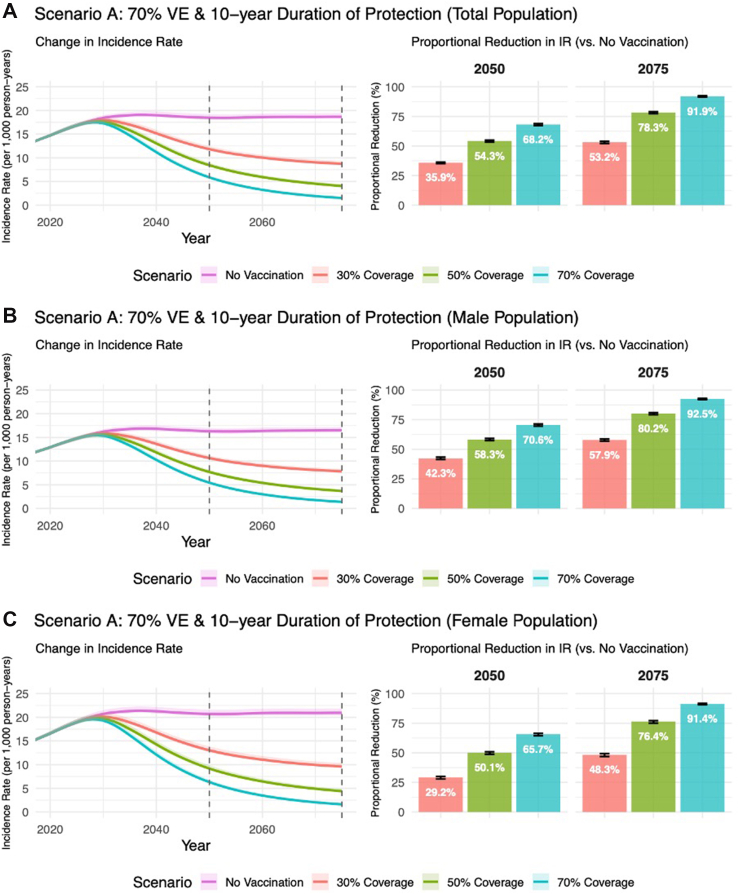
**The absolute and proportional reduction in CT incidence rates(IR)for Scenario A** in 2050 and 2075 when compared to the Referent Scenario (no vaccination), assuming 10-year duration of vaccine-conferred immunity and 70% efficacy (base-case assumption). Results are presented by vaccine coverage attained by 2035 and sex assigned at birth. CT incidence rates estimated among individuals aged 15–64 years. Sections A, B, and C represent the total population, male only population, and female only population, respectively.

**Fig. 4:**
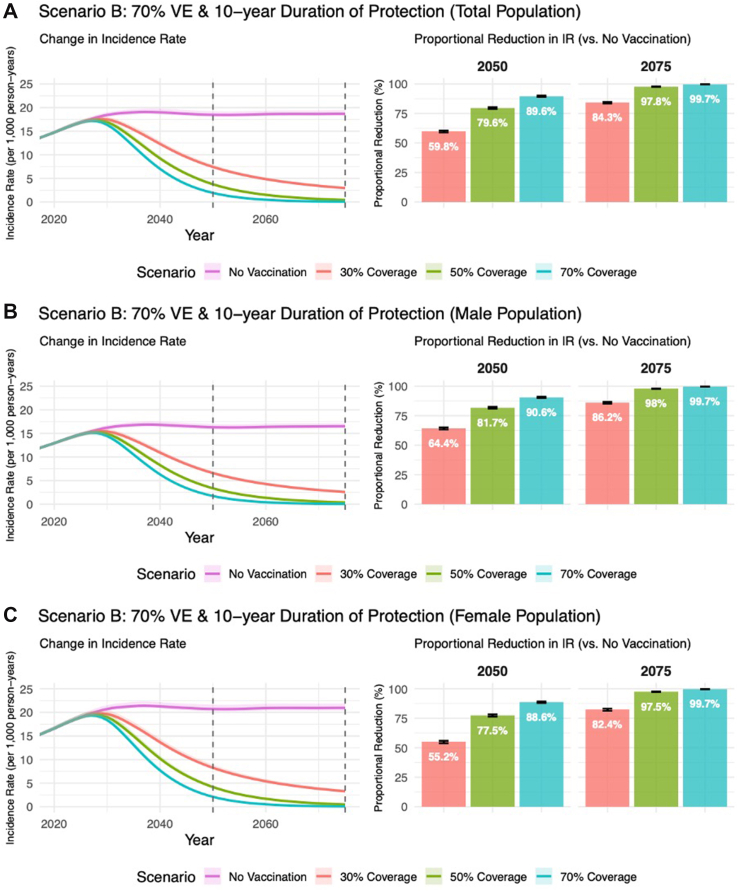
**The absolute and proportional reduction in CT incidence rates(IR)for Scenario B** in 2050 and 2075 when compared to the Referent Scenario (no vaccination), assuming 10-year duration of vaccine-conferred immunity and 70% efficacy (base-case assumption). Results are presented by vaccine coverage attained by 2035 and sex assigned at birth. CT incidence rates estimated among individuals aged 15–64 years. Sections A, B, and C represent the total population, male only population, and female only population, respectively.

In the absence of vaccination, chlamydia incidence rates among individuals aged 15–64 years increased from 16.9 infections per 1000 person-years (PYs) in 2025 to 18.7 by 2075. Female-only vaccination (Scenario A, Fig. 3) reduced incidence rates to 4.0 per 1000 PYs by 2075 (78.3% reduction), while sex-neutral vaccination (Scenario B, Fig. 4) further reduced rates to 0.4 per 1000 PYs (97.8% reduction). Notably, female-only (80.2% versus 76.4%) and sex-neutral routine vaccination (98.0% versus 97.5%) achieved slightly greater reductions in incidence rates by 2075 among males than females, respectively. Additional analysis assuming equal screening rates for males and females by age nearly eliminated this sex-specific difference, suggesting that the projected greater male benefit is largely driven by lower baseline screening in males. Catch-up campaigns (Scenarios C/D) provided marginal improvement in incidence rates by 2050 and 2075. Additional results from scenario analysis and sensitivity analysis can be found in Supplementary Figures 19 and S20 and Supplementary Tables S17–S21.

Vaccination efficiency, measured as infections prevented per 1000 doses, was higher for female-only (∼2500) versus sex-neutral vaccination (∼2000), reflecting more targeted coverage. More durable protection and higher efficacy consistently improved efficiency, as vaccines with ≥50% efficacy conferring ≥5 years of protection prevented more than one infection per dose across all coverage levels. Implementation of a catch-up campaign and higher routine coverage averted more total infections but reduced efficiency, indicating diminishing epidemiologic returns with broader uptake (see Supplementary Figures S21 and S22 and Supplementary Tables S22–S25).

Under base-case assumptions, all strategies substantially reduced sequelae compared to no vaccination (Supplementary Table S26). By 2075, female-only routine vaccination (Scenario A) averted an estimated 430,000 cases of epididymitis and 3.46 million cases of pelvic inflammatory disease (37.1% reduction), respectively. Sex-neutral vaccination (Scenario B) achieved greater impact, preventing 618,000 cases of epididymitis and 4.78 million cases of pelvic inflammatory disease (51.4% reduction), respectively. Adding a one-time catch-up campaign among unvaccinated females aged 15–24 years in 2035 provided modest additional benefits across all sequalae.

### Cost-effectiveness outcomes

Under base-case assumptions, vaccination among females aged 15 years (Scenario A) led to substantial benefits compared to current standards of care (Table 3). This strategy was dominant, with 254,841 QALYs gained and $1.97 billion USD in cost savings between 2025 and 2050. Adding a catch-up campaign for unvaccinated females aged 15–24 years in 2035 (Scenario C) was dominant over routine vaccination of females only, yielding an additional 23,836 QALYs and $210 million in cost savings compared to Scenario A.

**Table 3:** Sequential cost-effectiveness analysis for female-only, sex-neutral, and catch-up vaccination strategies, under base-case assumptions^a^ (2025–2050).

Step	Intervention	Comparator	Total cost (Million USD)^b^^,^^c^	Total QALY decrements^2^	Δ Cost (Million USD)	Δ QALYs gained	ICER ($/QALY)
**A. Female-only vaccination**							
1	No Vaccination	–	147,921.94	1,257,109	–	–	–
2	Scenario A	No Vaccination	145,948.52	1,002,268	−1973.42	254,841	**Cost Saving**
3	Scenario C	Scenario A	145,738.31	978,432	−210.20	23,836	**Cost Saving**
**B. Sex-neutral vaccination**							
1	No Vaccination	–	147,921.94	1,257,109	–	–	–
2	Scenario A	No Vaccination	145,948.52	1,002,268	−1973.42	254,841	**Cost Saving**
3	Scenario B	Scenario A	145,594.28	856,325	−354.23	145,943	**Cost Saving**
4	Scenario D	Scenario B	145,539.70	841,442	−54.59	14,883	**Cost Saving**

Scenario A: Vaccinating 15-year-old females entering the model between 2025 and 2075. Scenario B: Vaccinating 15-year-old females and males entering the model between 2025 and 2075. Scenario C: Scenario A at 50% coverage + catch-up campaign among unvaccinated 15–24-year-old females in 2035. Scenario D: Scenario B at 50% coverage + catch-up campaign among unvaccinated 15–24-year-old females in 2035.

Abbreviations: USD, United States Dollar, QALY, quality-adjusted life years; VE, vaccine efficacy.

a Base-case assumptions include Scenarios A-D: 50% coverage by 2035, 10-year duration of protection, and 70% VE; Scenarios C/D: 10% catch-up campaign coverage in 2035.

b Costs and QALYs are discounted at 3% annually.

c Costs are in 2025 USD.

When considering sex-neutral vaccination strategies, the addition of vaccinating males at age 15 (Scenario B) to the female-only strategy (Scenario A) was dominant, resulting in 145,943 QALYs gained and $354 million in cost savings. Expansion of Scenario B with catch-up campaigns for unvaccinated females aged 15–24 years in 2035 (Scenario D) led to an additional 14,883 QALYs and $55 million in cost savings. Although Scenario B provided meaningful benefits, it was dominated by Scenario D, which achieved greater health gains at lower cost. All strategies under the base-case assumption were cost-saving and well below conservative U.S. WTP thresholds of $50,000/QALY. Results from scenario analysis, assuming different vaccine characteristics and coverage beyond the base-case, can be found in Supplementary Figures S23–S25 and Supplementary Tables S27–S30.

### Sensitivity analysis

Across all comparisons, ICERs were most sensitive to vaccine price-per-dose. For female-only vaccination (Scenario A), ICERs ranged from cost-saving to $37,809/QALY and ceased to be cost-saving above $250 per dose. Other influential parameters included pelvic inflammatory disease treatment costs, screening, treatment, and vaccination visit costs, QALY estimates, and discounting, though results remained cost-saving. Results were similar when incrementally adding catch-up vaccination (Scenario C), with ICERs for price-per-dose ranging from cost-saving to $35,219/QALY, and remaining cost-effective under all other parameter variations.

Expanding to sex-neutral vaccination (Scenario B), ICERs were most sensitive to vaccine price-per-dose (cost-saving to $79,876/QALY), with loss of cost-saving results at a vaccine price-per-dose above $125. ICERs were also sensitive to pelvic inflammatory disease treatment costs (cost-saving to $1744/QALY) and vaccination visit costs (cost-saving to $1480/QALY). Similar results were observed for Scenario D, with ICERs sensitive to price-per-dose (cost-saving to $66,887/QALY) and pelvic inflammatory disease treatment costs (cost-saving to $624/QALY). Importantly, all ICERs remained well below common WTP thresholds ($100,000–$150,000/QALY), with most parameter values yielding cost-saving results. All results from sensitivity analysis can be found in Supplementary Figures S26–S29.

## Discussion

We developed a deterministic, compartmental model of heterosexual chlamydia transmission in the US to evaluate the potential impact and cost-effectiveness of hypothetical vaccination strategies beginning in 2025. Under base-case assumptions, vaccination of adolescent females averted 37.4% of cumulative infections from 2025 to 2075 and reduced the annual incidence rate in 2075 by 78.3% compared with no vaccination. Vaccinating adolescent females and males provided additional benefits to cumulative infections (52.3% reduction) and incidence (97.8% reduction). Female-only and sex-neutral routine vaccination prevented 3.46 million and 4.78 million cases of pelvic inflammatory disease by 2075, respectively, with further reductions in downstream sequalae contributing to reproductive morbidity. Counterintuitively, vaccination was projected to have a greater relative impact on males because they are not routinely screened and thus stand to benefit more from additional prevention services. Catch-up vaccination of unvaccinated females provided incremental gains under conservative coverage assumptions used in our model. Higher catch-up coverage or broader eligibility criteria could produce greater epidemiologic and economic benefits, suggesting that catch-up strategies still hold important public health value depending on implementation. Nonetheless, sustained routine vaccination is likely to have the greatest impact on long-term reductions in disease burden. Importantly, we modeled only a single campaign in 2035 with limited coverage; therefore, broader or multi-year catch-up strategies, including vaccination of males, warrant further evaluation.

Durability of vaccine protection emerged as a key determinant of effectiveness. For example, with a sex-neutral vaccine of 70% efficacy reaching 50% coverage by 2035, extending durability from 5 to 20 years resulted in an 11% absolute increase in cumulative infections averted by 2075. This underscores the importance of developing vaccines with durable immunity, even at moderate efficacy. Our results also suggest that vaccines exceeding 50% efficacy and five years of protection would prevent more infections than doses administered, representing a benchmark for product development. While estimating infections averted per 1000 doses provided a reasonable metric of intervention efficiency, it does not capture economic effects. Diminishing returns at higher coverage levels, suggested by our analysis, do not necessarily translate into poorer cost-effectiveness, which requires explicit incorporation of costs and health outcomes.

When these elements were included, vaccination was consistently cost-saving or highly cost-effective. Adding routine vaccination of 15-year-old females to current screening practices was cost-saving, as was extending vaccination to 15-year-old males. Incorporation of a catch-up campaign among unvaccinated females aged 15–24 years also yielded cost savings. Findings remained consistent when efficacy, durability, and coverage assumptions were varied, with all scenarios producing ICERs that were cost-saving or cost-effective under WTP thresholds of $50,000/QALY. One-way sensitivity analyses showed ICERs were influenced by price-per-dose and costs of pelvic inflammatory disease treatment, with smaller effects from QALY estimates, discounting, and visit costs. Female-only vaccination was cost-saving across nearly all parameter values, while sex-neutral strategies showed greater uncertainty at higher vaccine prices, with ICERs exceeding $50,000/QALY. Even at $1000 per dose, ICERs for sex-neutral vaccination remained well below WTP thresholds of $100,000–$150,000/QALY, reinforcing the robustness of vaccination as a cost-effective intervention. Importantly, cost-effectiveness outcomes were evaluated over a shorter time horizon (2025–2050), and economic benefits are likely to increase over longer evaluation periods. To our knowledge, our findings are the first to estimate cost-effectiveness of chlamydia vaccination at the national level.

Our findings build on, and diverge from, prior studies of chlamydia vaccination. Makhoul et al. (2024) also found that sex-neutral vaccination could substantially reduce burden under optimistic implementation parameters (80% coverage by 2035 among 15–49-year-olds, 20-year protection, and 50% efficacy).^11^ In contrast, our base-case assumptions were intentionally more conservative to reflect the current US landscape where vaccine uptake is hindered by hesitancy, stigma, and access barriers.^28^ Despite these differences, both studies found that a partially efficacious vaccine could significantly reduce chlamydia burden, nationally. Although their study did not directly incorporate cost data, Makhoul et al. concluded that a vaccine could be cost-effective under realistic scenarios, a conclusion consistent with our findings.^11^

Our work extends findings from Owusu-Edusei et al. (2015) that a vaccination program targeting 14-year-old girls, with or without catch-up vaccination for women aged 15–24 years, would be cost-effective at $35,300/QALY and $53,200/QALY, respectively.^12^ While both studies used dynamic, compartmental models and a societal perspective, their simplified population of 100,000 individuals aged 15–24 years did not capture contributions from older age groups or age-based sexual mixing, limiting national representativeness. By incorporating age- and sex-structured compartments for those aged 15–64 years and calibrating to both national case and prevalence data, our model provided a comprehensive and realistic assessment of vaccination impacts, suggesting greater potential for cost-savings. Our results differ from Ditkowsky et al. (2018), who concluded that vaccination would reduce morbidity but increase healthcare costs by $41.77 million (2015 USD).^14^ This discrepancy largely reflects methodological differences. Their static Markov model did not capture herd effects, indirect costs, or longer-term dynamics. In contrast, our dynamic, nationally representative model incorporated costs averted among males, a longer time horizon, broader stratification, and more flexible vaccine assumptions. These differences allowed our model to capture wider benefits of vaccination, leading to findings of cost savings under certain scenarios.

Vaccination should be considered within the broader landscape of chlamydia prevention programs. Our model assumed ongoing screening, diagnosis, and treatment at current levels, reflecting integration of vaccination into existing infrastructure. Screening is currently used to detect infections, monitor transmission, and identify disparities in burden among at-risk populations, although the population-level effectiveness and optimal implementation of opportunistic testing strategies remain areas of debate.^7^^,^^29^ If vaccination were widely implemented and substantially reduced incidence, the need for routine screening in some populations may decline as underlying disease burden decreases. Changes in prevention strategies could in turn influence the long-term epidemiologic and economic impact of vaccination. However, experiences with other vaccines show that multi-component, tailored strategies can improve uptake in marginalized groups, and may be essential for equitable vaccine rollout given rising hesitancy and stigma around sexual health.^30^ Additional biomedical strategies, including doxy-PEP, may serve as complementary tools for those unwilling or unable to access vaccination.^9^ Future studies should evaluate how prevention strategies may evolve following vaccine introduction and how these changes may influence long-term cost-effectiveness.

Our study has important limitations. First, we did not explicitly stratify by sexual behavior, which may lead to underestimation of vaccine impact in individuals with higher behavioral risk factors.^11^^,^^12^ Instead, we used age- and sex-based differences in mixing as a proxy for risk heterogeneity to minimize the influence of uncertainty among sexual transmission parameters by age, sex, and risk groups on our model. While this approach aimed to capture key differences in transmission risk across demographic groups, explicit stratification by behavioral risk could further refine future modeling efforts as more robust data become available. Second, we modeled only heterosexual, urogenital transmission. Availability of nationally representative data on chlamydia burden among MSM is limited, and including both heterosexual and non-heterosexual mixing would have introduced additional structural complexity and uncertainty in the absence of necessary sexual behavior data. Although cases among MSM were indirectly included in calibration targets, they were not explicitly modeled as contributors to transmission. Given their smaller contribution to total infections, MSM-attributed cases likely have limited influence on model outcomes. Nonetheless, MSM represent a population with a disproportionate burden of chlamydia and would benefit from future modeling studies to elucidate the impact of targeted vaccination within this population.^1^ Third, the absence of stratification by race and ethnicity limited our ability to examine disparities, despite well-documented differences in chlamydia burden.^18^ Future models should seek to include race and ethnicity to capture heterogeneity in disease transmission and inform equitable vaccination strategies.

Additional uncertainty surrounds vaccine-specific parameters, including costs, efficacy, and duration of protection, in the absence of a candidate in late-stage development. The trajectory of vaccination coverage is highly uncertain and may differ from a simplified linear scale-up. Future studies could explore alternative vaccination uptake patterns informed by vaccine characteristics and the broader policy environment. Significant gaps also remain in our understanding of chlamydia natural history and transmission, particularly regarding the duration and strength of infection-conferred immunity. Some studies assumed longer-lasting full or partial immunity following infection, which could reduce reinfection rates and attenuate the projected incremental benefits of vaccination.^11^^,^^15^^,^^19^ To address this, we calibrated the model to both AtlasPlus and NHANES data using 100 parameter sets, then estimated epidemiologic outcomes and ICERs across this distribution under different assumptions of vaccine characteristics and implementation strategies.^18^^,^^22^ Variability in model fit across demographic groups reflected uncertainty in epidemiologic data and the calibration approach used in this study, which prioritized alignment with adjusted incidence estimates and improved fit in younger, higher-burden populations while accepting greater variability in lower-incidence groups. One-way sensitivity analyses further assessed the influence of parameter uncertainty on ICERs. Reliability of future models will depend on the continued collection of high-quality data to mitigate uncertainty in key parameters.

Next, we did not model vaccine impacts on infectiousness, duration, or symptom presentation, though these could enhance cost savings, as highlighted previously.^11^ Instead, vaccination was assumed to reduce only the probability of acquiring infection. This assumption provides a conservative estimate of vaccine impact in the absence of empirical data on vaccine performance. If vaccination also reduces infectiousness or shortens infection duration among breakthrough infections, the resulting reductions in transmission could further enhance impact and cost-effectiveness. Our analysis is also specific to the US, as underlying epidemiological burden of chlamydia, direct and indirect costs, and transmission patterns may differ from other settings. Additional studies are needed to assess the generalizability of our findings in settings outside the US.

While our cost estimates for pelvic inflammatory disease incorporated the average case, accounting for the probability of subsequent complications, they may underestimate the true burden of infertility, including acute and long-term mental health impacts.^31^ As a result, vaccine-attributable cost savings may be underestimated. In contrast, vaccination costs in our analysis included per-dose administration costs but did not explicitly incorporate one-time program introduction costs (e.g., training, communication campaigns, system start-up), which could increase overall program costs and decrease projected cost savings. Additional implementation costs associated with catch-up campaigns conducted after routine vaccine introduction were also not explicitly modeled, although introducing catch-up vaccination alongside routine rollout could generate efficiencies through shared training and outreach activities. However, sensitivity analyses explored vaccine prices up to $1000/dose, substantially higher than expected market prices, suggesting that even considerably higher delivery or introduction costs would be unlikely to alter findings. We also did not model multi-dose series or boosters following waned vaccine-conferred immunity, which could increase costs necessary for durable protection. Finally, our model assumed routine vaccination at age 15 years to approximate immunization prior to sexual debut, a strategy that maximizes the preventive potential of vaccination by providing protection before exposure occurs. Routine vaccination implemented across a broader adolescent age range or after sexual debut may yield smaller reductions in infections and attenuate projected cost-effectiveness.

To our knowledge, this is the first chlamydia vaccination model to incorporate annual cases and prevalence data in model calibration, strengthening the validity of our findings.^18^^,^^22^ By including individuals aged 15–64 years, we captured both direct benefits that vaccination provides to younger individuals who are recipients of the intervention and indirect benefits to older individuals who remain unvaccinated but are still at risk of infection. By exploring multiple strategies, including female-only or sex-neutral routine vaccination and catch-up campaigns, under varying coverage, efficacy, and duration assumptions, we also evaluated realistic implementation scenarios and vaccine characteristics which may inform future public health decisions. Importantly, we simulated less favorable coverage assumptions to account for real-world challenges in STI vaccine uptake, informed by experience with HPV vaccination^28^ Our cost-effectiveness analysis incorporated updated US cost estimates and productivity losses, reflecting a societal perspective. Finally, structural detail, including age- and sex-based sexual mixing, provided the necessary framework for assessing vaccination impact and cost-effectiveness at the population-level to inform future policy recommendations.

Ultimately, our findings suggest that even a partially efficacious chlamydia vaccine could meaningfully advance US efforts to reduce disease burden. Vaccination, when implemented among adolescent females or through broader, sex-neutral strategies, has the potential to be cost-saving or highly cost-effective across a range of realistic scenarios. Importantly, vaccination should be viewed as a complement for ongoing prevention programs. By using a dynamic, age- and sex-stratified transmission model, we captured both direct and indirect benefits of vaccination while addressing key uncertainties in disease burden and vaccine performance. As vaccine development advances, these results underscore the importance of continued investment in vaccine research, integration of emerging evidence on chlamydia natural history, and refinement of mathematical models to guide equitable implementation across diverse populations and geographies.

## Contributors

All authors have contributed significantly to the conceptualization, analysis, synthesis, and write-up of this work. GKZ and DD directly accessed and verified the underlying data reported in the manuscript. All authors were responsible for the decision to submit the manuscript.

## Data sharing statement

All data used in this study are publicly available and documented in the referenced literature and Supplementary Appendix.

## Declaration of interests

G.K.Z. has received a research grant from the NIH [1F31AI181431-01A1], related to this work. G.K.Z. has also gained employment as a Clinical Health Equity Consultant with Boston Scientific, unrelated to this work, and received travel support from the Gates Foundation, unrelated to this work. D.D. has received a grant from the NIH [UM1 AI068617, paid to Fred Hutch], related to this work. D.D. has received grants from the NIH [R01 AI179417 paid to Fred Hutch], U.S. CDC [NU38OT000297 paid to Fred Hutch], and Gilead Sciences, Inc. [SRA240503 paid to Fred Hutch], all unrelated to this work. D.D. provided consultancy for a joint modeling project of the HIV Modeling Consortium with the University of South Africa. C.M.K. has received grants from the NIH and Gilead Sciences, Inc., unrelated to this work. C.M.K. has received research supplies from Hologic, Inc., unrelated to this work. A.D. has received research grants from the NIH, Gilead Sciences, Inc., and the UC Berkeley Forum for Collaborative HIV Research, all unrelated to this work. A.D. has received travel support and provision of HPV vaccine from Merck & Co., Inc., unrelated to this work. C.E.L. has no conflicts of interest to report.
