# Supplementary material for: Evaluating the potential health and economic impacts of chlamydia vaccination strategies in the United States: a mathematical modeling and cost-effectiveness simulation study
Source: Lancet Reg Health Am. 2026 May 26;60:101502. doi: 10.1016/j.lana.2026.101502 (PMC13234485; doi:10.1016/j.lana.2026.101502)
Supplement: Supplementary Material [file mmc1.docx]

**Evaluating the Potential Health and Economic Impacts of Chlamydia Vaccination Strategies in the United States: A Mathematical Modeling and Cost-Effectiveness Simulation Study - Supplementary Appendix**

Gregory K. Zane, PhD, MPH, Dobromir Dimitrov, PhD, Carol E. Levin, PhD, MSc, Christine M. Khosropour, PhD, MPH, Ann Duerr, MD, PhD, MPH

**Table of Contents:**

**Supplementary Methods ...……………………………………………………………………....2**

**Appendix A. Model Development……………………………………………………………….….. 2**

Appendix A.1. CT Transmission Model…………………………………………………………... 2

A.1.1. Model Summary…………………………………………………………................2

A.1.2. Detailed Model Description …………………………………………..…................2

Appendix A.2. Data Sources & Parameterization………………………………………………….4

A.2.1. Demographic Data ………………………………………………………................4

A.2.2. CT Surveillance Data ……………………………………………………................4

A.2.3. Model Parameters ………………………………………………………………….9

Appendix A.3. Model Calibration & Uncertainty Assessment………………………………….....9

A.3.1. Calibration Approach ………………………………………………………………9

A.3.2. Validation with NHANES Data ……………………………………………………9

A.3.3. Results of Model Calibration ………………………………………………………9

**Appendix B. Epidemiologic Outcomes ...………………………………………………………….11**

Appendix B.1. Referent and Intervention Scenarios (Base-Case) ………………………….…….11

Appendix B.2. Vaccination Scenarios ……………………………………………………………11

Appendix B.3. Epidemiologic Outcomes …..…………………………………………………….11

**Appendix C. Costs, Health Utilities, and Cost-effectiveness Analyses ………………………….12**

Appendix C.1. Health Outcomes and Cost Equations ……………………………………………12

C.1.1. Health Outcome Parameters, Equations, and Discounting ……………………….12

C.1.2. Cost Outcome Parameters, Equations, and Discounting…………...……………..12

C.1.3. Outline of Model-specific Parameters for Health and Cost Outcome Equations....14

Appendix C.2. QALY and Cost Estimates ……………………………………………………….14

C.2.1. Cost Estimates …………………………………………………………………….14

C.2.2. Health Utility (QALY) Estimates …………………………………………………14

Appendix C.3. Referent and Intervention Scenarios……………………………………………...14

Appendix C.4. ICER Estimation ……………………………………………………………...…..14

Appendix C.5. Scenario Analysis ……………………………………………………………...…14

Appendix C.6. Sensitivity Analysis ……………………………………………………………....15

**Supplementary Results…………………………………………………………………………16**

**Appendix D. Epidemiologic Outcomes ...………………………………………………………….16**

Appendix D.1. Scenario Analysis - Absolute and Relative Reductions in CT Infections ………..16

Appendix D.2. Scenario Analysis – Reductions in CT Incidence Rates………………………….16

Appendix D.3. Scenario Analysis – Vaccination Efficiency ……………………………………..16

Appendix D.4. Secondary Analysis – Sequalae Prevented (Base-case) ………………………….16

**Appendix E. Costs, Health Utilities, and Cost-effectiveness Analyses …………………………..17**

Appendix E.1. Scenario Analysis ……………………………………………………………...….17

Appendix E.2. Sensitivity Analysis ……...………………………………………………………..17

**Appendix F. Consolidated Health Economic Evaluation Reporting Standards (CHEERS) 2022 Checklist ………….……………………………………………...………………………………….18**

**Supplementary Figures………………………………………………………………………....20**

**Supplementary Tables…………………………………………………………………………. 45**

**Supplementary References……………………………………………………………………..78**

**Supplementary Methods**

**Appendix A. Model Development**

**Appendix A.1 CT Transmission Model**

**A.1.1. Model Summary**

An age- and sex-structured deterministic, compartmental mathematical model using ordinary differential equations was constructed to simulate transmission of CT among individuals aged 15 to 64 years in the U.S. from 2000 to 2075 and assess the impacts of CT vaccination strategies on disease burden from 2025 to 2075 (see **Figure S1**). The model was structured using a contemporary understanding of disease transmission and natural history, leveraging other published models of sexually transmitted infections.^1–6^ The simulated population was further stratified by vaccination status and disease stage: susceptible (S), exposed non-infectious (E), infectious symptomatic infection (I_S_), infectious asymptomatic infection (I_A_), sequelae-experiencing (Q), or infection-conferred immunity(R). Sequelae were defined as epididymitis for males or PID for females. Infection-conferred immunity occurred among individuals who naturally cleared their infection without treatment. Immunity was assumed to be short in duration and individuals returned to the susceptible population once immunity waned. The population was further stratified into six groups by biological sex at birth and age: males and females aged 15–24, 25–39, and 40–64 years. Age groups were selected *a priori* to reflect the higher burden of CT among individuals aged 15–24 years compared to those 25 years and older.^7,8^ Partial assortative mixing by age was assumed as individuals preferred to form sexual partnerships within the same age group.^9,10^ Our model does not explicitly account for same-sex transmission, instead focusing on the substantial burden of chlamydia among individuals with female reproductive anatomy who subsequently experience the most severe sequelae following infection .^8,11^

**A.1.2. Detailed Model Description**

The model comprises 72 ordinary differential equations, structured across 12 disease stage compartments, 2 biological sex groups, and 3 age groups, with indexing as follows:

- Biological sex at birth *j* ∈ {1 = male; 2 = female}
- Age group *k* ∈ {1 = 15-24 years; 2 = 25-39 years; 3 = 40-64 years}

Compartments among the simulated, unvaccinated population include:

- *S_j,k_* : Susceptible population of sex *j* and age group *k*
- *E_j,k_* : Infected, non-infectious population of sex *j* and age group *k*
- *Is_j,k_* : Symptomatically infected, infectious population of sex *j* and age group *k*
- *Ia_j,k_* : Asymptomatically infected, infectious population of sex *j* and age group *k*
- *Q_j,k_* : Sequelae-experiencing population of sex *j* and age group *k*, where *j* = 1 for epididymitis; *j* = 2 for pelvic inflammatory disease (PID)
- *R_j,k_* : Infection-conferred immune population (natural recovery) of sex *j* and age group *k*

Compartments among the simulated, vaccinated population include:

- *S^V^_j,k_* : Susceptible population of sex *j* and age group *k*
- *E^V^_j,k_* : Infected, non-infectious population of sex *j* and age group *k*
- *Is^V^_j,k_* : Symptomatically infected, infectious population of sex *j* and age group *k*
- *Ia^V^_j,k_* : Asymptomatically infected, infectious population of sex *j* and age group *k*
- *Q^V^_j,k_* : Sequelae-experiencing population of sex *j* and age group *k*, where j = 1 for epididymitis; *j* = 2 for pelvic inflammatory disease (PID)
- *R^V^_j,k_* : Infection-conferred immune population (natural recovery) of sex *j* and age group *k*

Model parameters include:

- *η* : Relative size of the population entering the model at age 15 (“birth” rate), dependent on time *t*
- *N* : Total size of the simulated population, dependent on time *t*
- *ξ* : Migration rate into susceptible, unvaccinated compartment (*S_j,k_*), dependent on time *t*
- *μ* : Background mortality rate among simulated population, dependent on time *t*
- *χ _j,k_* : Rate of aging transition, among individuals from age group *k* to age group *k+1*, with aging into the youngest age group (*k = 1*) defined by separate ‘birth rate’ parameters
- *Vx _j_* : Proportion of individuals of sex *j* vaccinated upon entering the population at age 15 years
- *β _j,k_* : Rate of vaccination coverage via catch-up campaigns, among individuals of sex *j* and age group *k*
- *θ _k_* : Rate of wanning vaccination immunity
- *VE_S_* : Vaccine efficacy, defined as the proportional reduction in susceptibility to infection, comparing vaccinated to unvaccinated individuals
- *λ _j,k_* : Force of infection on susceptible individuals of sex *j* and age group *k*
- *ϕ* : Rate of transition of infected individuals from exposed, non-infectious to infectious (1/incubation period)
- *ζ _j_* : Proportion of infections that present symptomatically, among individuals of sex *j*
- *τ _j,k_* : Recovery rate of symptomatically infected individuals due to treatment, among individuals of sex *j* and age group *k*, calculated as the product of:
  - *dur_tx _j_* : Duration of treated infection, among individuals of sex *j*
  - *ε* : Sensitivity of diagnostic testing for CT detection
  - *p_txt* : Probability of treatment for symptomatic infection
  - *tx_eff* : Efficacy of antibiotic treatment among individuals receiving treatment
- *γ _j,k_* : Recovery rate of asymptomatically infected individuals due to screen-and-treat, among individuals of sex *j* and age group *k*, calculated as the product of:
  - *r _j,k_* : Annual screening rate for CT, among individuals of sex *j* and age group *k*
  - *ε* : Sensitivity of diagnostic testing for CT detection
  - *υ* : Probability of post-screening treatment among individuals following a positive nucleic acid amplification test (NAAT) result
  - *tx_eff* : Efficacy of antibiotic treatment among individuals receiving treatment
- *ι* : Rate of natural resolution among untreated symptomatic or asymptomatic infection
- *κ _j_* : Proportion of resolved symptomatic or asymptomatic infections that develop into sequelae, among individuals of sex *j*
- *ϖ _j_* : Rate of sequelae resolution, among individuals of sex *j*
- *ψ* : Rate of waning infection-conferred immunity

The force of infection *λ _j,k_* on susceptible individuals of sex *j* and age group *k* was estimated as:

$\lambda_{j,k}=c_{j,k}*\sum_{j^{'}} \left[ (1-\delta_{jj^{'}})\sum_{k^{'}} {t_{j,k,j^{'},k^{'}}*m}_{j,k,j^{'},k^{'}}\frac{I_{j^{'},k^{'}}^{Inf}}{N_{j^{'},k^{'}}} \right]$, where:

- *c _j,k_* : Sexual partner change (number of sexual partners in the past 12 months), among susceptible individuals of sex *j* and age group *k*
- $\delta_{jj^{'}}$ : Kronecker delta function, where $\delta_{jj^{'}}$= 1 if individuals of *j* = *j’*, ensuring heterosexual mixing only
- $t_{j,k,j^{'},k^{'}}$ : Transmission probability per sexual partnership from an infected individual of sex *j’* and age group *k’* to a susceptible individual of sex *j* and age group *k*
- $m_{j,k,j^{'},k^{'}}$ : Number of partners that susceptible individual of sex *j* and age group *k* has from sex *j’* and age group *k’.* It is based on the sexual mixing equation, representing partial assortative mixing between age groups *k* and *k'*, estimated as:

$m_{j,k,j^{'},k^{'}}= (1-\delta_{jj^{'}})\left[ \rho\delta_{kk^{'}}+(1-\rho)\left( \frac{c_{j^{'},k^{'}}*N_{j^{'},k^{'}}}{\sum_{k^{'}} c_{j^{'},k^{'}}*N_{j^{'},k^{'}}} \right) \right]$, where:

- - *ρ* : Assortative coefficient, where *ρ* = 0 if fully proportionate mixing and *ρ* = 1 if fully assortative mixing
  - $c_{j^{'},k^{'}}$ : Sexual partner change (number of sexual partners in the past 12 months), among sexual contacts of sex *j’* and age group *k’*
  - $N_{j^{'},k^{'}}$ : Total population size among sexual contacts of sex *j’* and age group *k’*
- $I_{j^{'},k^{'}}^{Inf}$ : Infectious individuals among sex *j’* and age group *k’*, dependent on time *t* estimated as:

$I_{j^{'},k^{'}}^{Inf}={Is}_{j^{'},k^{'}}+{Ia}_{j^{'},k^{'}}+{Is}_{j^{'},k^{'}}^{V}+{Ia}_{j^{'},k^{'}}^{V}$

The model is defined by the following sets of differential equations:

*Unvaccinated Individuals*

$$\frac{d}{dt}S_{j,k}= -\lambda_{j,k}S_{j,k}-\beta_{j,k}S_{j,k}-\chi_{j,k}S_{j,k}-\mu S_{j,k}+\varpi_{j}Q_{j,k}+\psi R_{j,k}+\tau_{j,k}{Is}_{j,k}+\gamma_{j,k}{Ia}_{j,k}+\left( 1-{Vx}_{j} \right){\eta N}_{j}+\theta_{k}S_{j,k}^{V}+\xi N_{j,k}+\chi_{j,k-1}S_{j,k-1}$$

$$\frac{d}{dt}E_{j,k}=-\beta_{j,k}E_{j,k}-\varphi E_{j,k}-\chi_{j,k}E_{j,k}-\mu E_{j,k}+\lambda_{j,k}S_{j,k}+\theta_{k}E_{j,k}^{V}+\chi_{j,k-1}E_{j,k-1}$$

$$\frac{d}{dt}{Is}_{j,k}=-\tau_{j,k}{Is}_{j,k}-\kappa_{j}\iota{Is}_{j,k}-{(1-\kappa}_{j})\iota{Is}_{j,k}-\chi_{j,k}{Is}_{j,k}-\mu{Is}_{j,k}+\zeta_{j}\varphi E_{j,k}+\theta_{k}{Is}_{j,k}^{V}+\chi_{j,k-1}{Is}_{j,k-1}$$

$$\frac{d}{dt}{Ia}_{j,k}=-\beta_{j,k}{Ia}_{j,k}-\gamma_{j,k}{Ia}_{j,k}-\kappa_{j}\iota{Ia}_{j,k}-{(1-\kappa}_{j})\iota{Ia}_{j,k} {- \chi}_{j,k}{Ia}_{j,k}-\mu{Ia}_{j,k}+{(1-\zeta}_{j})\varphi E_{j,k}+\theta_{k}{Ia}_{j,k}^{V}+\chi_{j,k-1}{Ia}_{j,k-1}$$

$$\frac{d}{dt}Q_{j,k}=-\varpi_{j}Q_{j,k} {- \chi}_{j,k}Q_{j,k}-\mu Q_{j,k}+\kappa_{j}\iota({Is}_{j,k}+{Ia}_{j,k})+\theta_{k}Q_{j,k}^{V}+\chi_{j,k-1}Q_{j,k-1}$$

$$\frac{d}{dt}R_{j,k}=-\beta_{j,k}R_{j,k}-\psi R_{j,k}-\chi_{j,k}R_{j,k}-\mu R_{j,k}+{(1-\kappa}_{j})\iota({Is}_{j,k}+{Ia}_{j,k})+\theta_{k}R_{j,k}^{V}+\chi_{j,k-1}R_{j,k-1}$$

*Vaccinated Individuals*

$$\frac{d}{dt}S_{j,k}^{V}= -{\left( 1-{VE}_{S} \right)\lambda}_{j,k}S_{j,k}^{V}-\theta_{k}S_{j,k}^{V}-\chi_{j,k}S_{j,k}^{V}-\mu S_{j,k}^{V}+\varpi_{j}Q_{j,k}^{V}+\psi R_{j,k}^{V}+\tau_{j,k}{Is}_{j,k}^{V}+\gamma_{j,k}{Ia}_{j,k}^{V}+{Vx}_{j}{\eta N}_{j}+\chi_{j,k-1}S_{j,k-1}^{V}+\beta_{j,k}S_{j,k}$$

$$\frac{d}{dt}E_{j,k}^{V}=-\varphi E_{j,k}^{V}-\theta_{k}E_{j,k}^{V}-\chi_{j,k}E_{j,k}^{V}-\mu E_{j,k}^{V}+{\left( 1-{VE}_{S} \right)\lambda}_{j,k}S_{j,k}^{V}+\chi_{j,k-1}E_{j,k-1}^{V}+\beta_{j,k}E_{j,k}$$

$$\frac{d}{dt}{Is}_{j,k}^{V}=-\tau_{j,k}{Is}_{j,k}^{V}-\kappa_{j}\iota{Is}_{j,k}^{V}-{(1-\kappa}_{j})\iota{Is}_{j,k}^{V}-\theta_{k}{Is}_{j,k}^{V}- \chi_{j,k}{{Is}^{V}}_{j,k}-\mu{Is}_{j,k}^{V}+\zeta_{j}\varphi E_{j,k}^{V}+\chi_{j,k-1}{{Is}^{V}}_{j,k-1}$$

$$\frac{d}{dt}{Ia}_{j,k}^{V}=-\gamma_{j,k}{Ia}_{j,k}^{V}-\kappa_{j}\iota{Ia}_{j,k}^{V}-{(1-\kappa}_{j})\iota{Ia}_{j,k}^{V}-\theta_{k}{Ia}_{j,k}^{V} {-\chi}_{j,k}{Ia}_{j,k}^{V}-{\mu Ia}_{j,k}^{V}+{(1-\zeta}_{j})\varphi E_{j,k}^{V}+\chi_{j,k-1}{Ia}_{j,k-1}^{V}+\beta_{j,k}{Ia}_{j,k}$$

$$\frac{d}{dt}Q_{j,k}^{V}=-\varpi_{j}Q_{j,k}^{V} -\theta_{k}Q_{j,k}^{V}{- \chi}_{j,k}Q_{j,k}^{V}-\mu Q_{j,k}^{V}+\kappa_{j}\iota({Is}_{j,k}^{V}+{Ia}_{j,k}^{V})+\chi_{j,k-1}Q_{j,k-1}^{V}$$

$$\frac{d}{dt}R_{j,k}^{V}=-\psi R_{j,k}^{V}{- \theta}_{k}R_{j,k}^{V}-\chi_{j,k}R_{j,k}^{V}-\mu R_{j,k}^{V}+{(1-\kappa}_{j})\iota({Is}_{j,k}^{V}+{Ia}_{j,k}^{V})+\chi_{j,k-1}R_{j,k-1}^{V}+\beta_{j,k}R_{j,k}$$

**Appendix A.2 Data Sources & Parameterization**

**A.2.1. Demographic Data**

Age- and sex-specific demographics from 2000 to 2075 were based on 2024 Revision of World Population Prospects, provided by the Population Division of the Department of Economic and Social Affairs of the United Nations.^12^ Rates for model entry, aging, background mortality, and migration were estimated to fit United Nations data by sex and year (**Figure S2**).

**A.2.2. CT Surveillance Data**

Annual reported age- and sex-specific CT cases between 2000 to 2022 are publicly available through the U.S. Centers for Disease Control and Prevention’s (CDC) National Center for HIV/AIDS, Viral Hepatitis, STD, and TB Prevention (NCHHSTP) AtlasPlus tool.^13^ Reported cases are impacted by several factors, including changes in treatment-seeking behaviors, screening coverage, diagnostic misclassification, and reporting practices, in addition to limitations in the complete capture of asymptomatic infections.^14,15^ Consequently, CT cases in AtlasPlus underestimate the true number of incident CT infections in a given year. A study by Learner et al. (2020) quantified this underestimation among U.S. women aged 15–24 years from 2000 to 2017.^14^ They found that accounting for incomplete screening, imperfect diagnostic tests, and under-reporting led to substantially higher estimates of CT burden in young women across the study period when compared to reported estimates. As biases in these factors have diminished over time, the gap between reported and adjusted CT cases has narrowed since 2000.

To use AtlasPlus data for model calibration, we expanded the analytical approach used in Learner et al. (2020) to estimate adjusted annual CT case counts by sex, age group, and year, accounting for parameters including imperfect reporting, diagnostic test misclassification, imperfect screening coverage, reason for CT testing, and uncaptured asymptomatic infections among populations unlikely to receive annual screen-and-treat services (i.e., men who have sex with women).^14^ Imperfect screening coverage refers to suboptimal uptake of recommended annual screening among eligible populations. Within each year between 2000 to 2022, 200 random draws from triangular distributions were taken to define parameter inputs. Distributions were determined through a combination of assumptions from Learner et al. (2020) and other published sources.^14,16,17^ Using the values in each individual draw, adjusted number of cases were estimated for each year, stratified by the six demographic groups defined in the model (males 15-24 years, males 25-39 years, males 40-64 years, females 15-24 years, females 25-39 years, and females 40-64 years). The median adjusted case count by demographic group and year was estimated across each of the 200 draws and corresponding 95% confidence intervals were calculated. A detailed description of the analytic approach, details on parameter selection, methodological limitations, and results are provided below:

Analytic Approach - Females

Adjusted annual CT cases among females aged 15-24, 25-39, and 40-64 years were estimated as the sum of infections due to either treatment-seeking behaviors (symptomatic infections, exposure, etc.) or annual screen-and-treat services (asymptomatic infections) in a given year. Case counts from treatment-seeking behaviors in a given year (*A_T_*) were calculated by adjusting the reported cases from AtlasPlus (*C_R_*) due to treatment-seeking behaviors (1 - *p_scr_*) and imperfect reporting to the CDC (*R*), where *p_scr_* represents the proportion of all diagnosed CT cases due to screening:

$$A_{T}= \frac{C_{R}* {(1-p}_{scr})}{R}$$

This assumes all diagnosed cases from treatment-seeking behaviors were reported to the CDC. Adjusted CT case counts from annual asymptomatic screening (*A_S_*) were estimated through a multi-step process accounting for under-reporting, diagnostic test performance, and screening coverage. Sensitivity (*Se*) and specificity (*Sp*) of CT diagnostic testing were calculated as weighted averages dependent on the sensitivity and specificity of NAAT (*SeNAAT*; *SpNAAT*) and non-NAAT (*SeNonNAAT*; *SpNonNAA*T) diagnostic tests and the proportion of NAAT tests among all diagnostic tests performed in a given year (*PrNAAT*):

$$Se=\left( PrNAAT*SeNAAT \right)+\left( 1-PrNAAT \right)(SeNonNAAT)$$

$$Sp=\left( PrNAAT*SpNAAT \right)+(1-PrNAAT)(SpNonNAAT)$$

The prevalence of true asymptomatic CT in the screened population (*P)* was estimated using reported cases due to screening (*C_R_* x *p_scr_*), reporting fraction (*R*), the total at-risk population (*N_F_*), age- and year-specific screening coverage among the at-risk population (*Sc*), and weighted-average diagnostic test performance (*Se*; *Sp*). The total at-risk population (*N_F_*) was estimated as the product of the total population size and the proportion of women eligible for screening, by age and year.

$$P= \frac{\frac{C_{R}*p_{scr}}{R}-Sc*N_{F}(1-Sp)}{Sc*N_{F}(Se+Sp-1)}$$

False positive diagnoses among females screened (*FrT*) were estimated using the weighted-average specificity (*Sp)*, prevalence of true asymptomatic CT in the screened population (*P*), the screening coverage (*Sc*), and the total at-risk population (*N_F_*):

$$FrT=\left( 1-Sp \right)\left( 1-P \right)*Sc*N_{F}$$

The number of true CT cases among females screened in a given year (*A*) was estimated as:

$$A= \frac{\frac{C_{R}*p_{scr}}{R}-FrT}{Se}$$

Finally, the total number of infections that would be expected as a result of asymptomatic screening (*A_s_*), assuming perfect reporting, diagnostic testing, and screening coverage, were estimated as the quotient of the number of true CT cases among females screened in a given year (*A*) and the screening coverage (*Sc*):

$$A_{S}=\frac{A}{Sc}$$

The total adjusted, annual CT cases (*Total Cases_Female_*) were the sum of infections due to treatment-seeking behaviors (*A_T_*) and annual screening (*A_S_*):

$${Total Cases}_{Female}=A_{T}+A_{S}$$

Analytic Approach – Males

Adjusted annual CT cases among males aged 15-24, 25-39, and 40-64 years were estimated as the sum of infections due to either treatment-seeking behaviors (symptomatic infections, exposure, etc.), annual screen-and-treat services (asymptomatic among men who have sex with men, high-risk men who have sex with women, etc.), or asymptomatic infections unaccounted for by treatment-seeking behaviors or screen-and-treat services (low-risk men who have sex with women) in a given year. Low-risk men who have sex with women are unlikely to receive any screening services; therefore, diagnosis of cases in this population rely on treatment seeking behaviors that would not capture all asymptomatic infections and need to be further accounted for in case adjustments.^18^

Case counts from treatment-seeking behaviors in a given year (*A_T_*) were calculated by adjusting the reported cases from AtlasPlus (*C_R_*) due to treatment-seeking behaviors (1 - *p_scr_*) and imperfect reporting to the CDC (*R*), where *p_scr_* represents the proportion of all diagnosed CT cases due to screening:

$$A_{T}= \frac{C_{R}* {(1-p}_{scr})}{R}$$

This assumes all diagnosed cases from treatment-seeking behaviors were reported to the CDC. Adjusted CT case counts from annual asymptomatic screening (*A_S_*) were estimated through a multi-step process accounting for under-reporting, diagnostic test performance, and screening coverage. Sensitivity (Se) and specificity of CT diagnostic testing were calculated in the same manner as above:

$$Se=\left( PrNAAT*SeNAAT \right)+\left( 1-PrNAAT \right)(SeNonNAAT)$$

$$Sp=\left( PrNAAT*SpNAAT \right)+(1-PrNAAT)(SpNonNAAT)$$

The prevalence of true asymptomatic CT in the screened population (*P)* was estimated using reported cases due to screening (*C_R_* x *p_scr_*), reporting fraction (*R*), the total at-risk population (*N_M_*), age- and year-specific screening coverage among the at-risk population (*Sc_M_*), and weighted-average diagnostic test performance (*Se*; *Sp*). The total at-risk population (*N_M_*) was estimated as the product of the total population size and the proportion of all males eligible for screening (men who have sex with men, high-risk men who have sex with women, etc.), by age and year.

$$P= \frac{\frac{C_{R}*p_{scr}}{R}-{Sc}_{M}*N_{M}(1-Sp)}{{Sc}_{M}*N_{M}(Se+Sp-1)}$$

False positive diagnoses among males screened (*FrT*) were estimated using the weighted-average specificity (*Sp)*, prevalence of true asymptomatic CT in the screened population (*P*), the screening coverage (*Sc*), and the total at-risk population (*N_M_*):

$$FrT=\left( 1-Sp \right)\left( 1-P \right)*{Sc}_{M}*N_{M}$$

The number of true CT cases among males screened in a given year (*A*) was estimated as:

$$A= \frac{\frac{C_{R}*p_{scr}}{R}-FrT}{Se}$$

Finally, the total number of infections that would be expected as a result of asymptomatic screening (*A_s_*), assuming perfect reporting, diagnostic testing, and screening coverage, were estimated as the quotient of the number of true CT cases among males screened in a given year (*A*) and the screening coverage (*Sc_M_*):

$$A_{S}=\frac{A}{{Sc}_{M}}$$

Adjustment for asymptomatic infections not captured among males via treatment-seeking behaviors (*A_T_*) or screening (*A_S_*) in a given year (*A_A_*) were estimated as the quotient of adjusted treatment-seeking cases (*A_T_*) by the proportion of all infections that present asymptomatically among males (*PrAsymp*) over one minus the proportion of asymptomatically presenting infections:

$$A_{A}= \frac{A_{T} *PrAsymp}{1-PrAsymp}$$

Adjusted, annual CT cases were estimated as the sum of infections due to treatment-seeking behaviors (*A_T_*), annual screen-and-treat services (*A_S_*), and uncaptured asymptomatic infections (*A_A_*):

$${Total Cases}_{Male}=A_{T}+A_{S}+A_{A}$$

Parameter Selection

*Annual CT Cases:*

Annual CT cases from 2000 to 2022 by sex and age group were pulled directly from AtlasPlus (see **Figure S3**).^13^

*Reported Cases by Reason for Testing:*

Limited data are available quantifying the proportion of reported CT cases due to either treatment-seeking behaviors or annual screen-and-treat services. We developed sex- and age-specific triangular distributions for reported cases due to annual screening using data from Tao et al. 2024, with further refinement using data from additional sources.^16,19,20^ The modes for each distribution (males 15-24: 58·9%, males 25-39: 58·8%, males 40-64: 55·0%, females 15-24: 62·8%, females 25-39: 60·0%, females 40-64: 58·5%) remained fixed over time but were accompanied by +/- 5% lower and upper bounds.

*Reporting Fraction:*

Year-specific triangular distributions for reporting fraction (proportion of diagnosed cases successfully reported to CDC) were created using the same approach as Learner et al (2020).^14^ We assumed the mode of annual reporting fractions rose from 70% in 2000 to 95% in 2022, as to account for incomplete data entry, transmission errors, and the improvement of reporting systems over time. We also assumed an asymptotically decreasing range to the triangular distribution to account for improved reporting certainty over time (**Figure S4**).

*Weighted Diagnostic Test Performance:*

Year-specific triangular distributions for diagnostic test performance were created to account for the transition from non-NAAT to NAAT.^14^ Similar to Learner et al. (2020), we made the following assumptions:

- Non-NAAT Sensitivity: 75% (LB: 65%, UB: 85%)
- Non-NAAT Specificity: 99·5% (LB: 99%, UB: 100%)
- NAAT Sensitivity: 96% (LB: 93%, UB: 99%)
- NAAT Specificity: 99·5% (LB: 99%, UB: 100%)

A triangular distribution for the proportion of NAAT tests among all diagnostic tests performed in a given year was also estimated as to account for the increased use of NAAT testing over time (**Figure S5**).

Using the above distributions for NAAT performance, non-NAAT performance, and the proportion of NAAT tests over time, we estimated weighted sensitivity and specificity values by year (**Figure S6**).

*Total At-risk Population (N_F_ & N_M_):*

Estimates of the total at-risk population for annual screening were calculated using United Nations (U.N.) Population Projections data by demographic group.^12^ U.N. data by demographic group from 2000 to 2022 is presented in **Figure S7**.

To estimate the proportion of the population eligible for screening services (N_F_ and N_M_), we assumed all females aged 15-39 years could be screened annually, reflecting CDC recommendations for women 15-24 years and observed screening uptake in women aged 25-39 years, despite the lack of formal screening recommendations for this group.^18,21^ For females aged 40-64 years, where data is limited, we used a triangular distribution (mode: 15%, LB: 10%, UB: 20%) to account for lower screening uptake and greater uncertainty. Thus, we assume 15% of the total female population aged 40-64 years, based on U.N. population projections, were “at risk” for screening annually.

For males, we assumed that only those at greatest risk for CT acquisition would be eligible for screening. To estimate the proportion of males eligible for screening, we used published literature to develop age- and year-specific triangular distributions (**Figure S8**).^22–24^ These values were then applied to UN data to identify the population of males by age group that were “at risk” for screening annually.

*Screening Coverage in At-risk Population:*

We used a similar approach as Learner et al. (2020) to develop year- and age-specific triangular distributions for screening coverage estimates among females, further informed by published literature (**Figure S9**):^14,21,25,26^

Data on screening coverage among eligible males by age and year is limited. To estimate coverage, we used American Men's Internet Survey (AMIS) data (2013-2019) to approximate CT screening rates among eligible males.^17^ We assumed age-dependent screening coverage increased linearly from 2013-2022. For 2000-2012, we applied a similar linear increase but at half the annual rate to account for the influence of HIV PrEP uptake on screening practices in the later time period.^27–30^ To reflect uncertainty, we incorporated a ±5% lower and upper bound in all triangular distributions. Age- and year-specific distributions for males are shown in **Figure S10**. Importantly, these estimates represent screening coverage only among males eligible for screening, not the entire male population, which explains the relatively high coverage values.

*Proportion of Symptomatic Cases Among Males:*

A triangular distribution for the proportion of all CT cases presenting symptomatically was developed using published literature (mode: 50% (LB: 20%, UB; 80%)).^1,2,31^ We assumed that year and age did not impact this distribution.

Results

Adjusted annual CT cases are provided for females (**Figure S11**), males (**Figure S12**), and total population (**Figure S13**) for all years between 2000 and 2022. Values by year and demographic group are further presented in **Table S1**.

Limitations

There are important limitations to our analysis. First, several parameters were developed using limited data from published sources. As such, we included plausible distributions around parameter modes and took random draws from those distributions to account for uncertainty. Future research is necessary to refine adjusted case analyses, given these known data gaps. Second, we could not explicitly account for extragenital CT cases, and little is known about screening coverage and diagnostic test performance by age and sex over time for extragenital testing. Third, adjusted CT cases among males were estimated as the sum of infections due to treatment-seeking behaviors, annual screen-and-treat services, and uncaptured asymptomatic infections. Due to data limitations, uncaptured asymptomatic infections were estimated using the reported symptomatic cases and the proportion of all infections presenting asymptomatically in males. As we were not able to explicitly differentiate symptomatic infections by sub-populations (MSM vs MSW), our estimates of uncaptured asymptomatic infections may repeat infections covered by screening among MSM. Fourth, we could not account for false positives among treatment-seeking cases due to high uncertainty in identifying individuals classified as “at risk” for seeking treatment for symptomatic infections. However, it is unlikely that adjustment for false positives among treatment-seeking cases would impact our adjusted estimates substantially.

Despite uncertainty in the adjusted estimates, they provide a more accurate representation of true CT infections in the United States and are essential for model calibration. Relying on unadjusted AtlasPlus data would underestimate the impact of CT vaccination for both disease burden and costs.

**A.2.3. Model Parameters**

Model parameters, including those for CT natural history, sexual behaviors, and treatment, were collected through an assessment of peer-reviewed literature and publicly available data sources. When significant parameter uncertainty existed, arising from inconsistencies in the literature or lack of empirical data (e.g., CT vaccine efficacy, duration of vaccine-induced immunity), parameter ranges were informed by existing evidence and reference values from analogous STI vaccines, including the human papillomavirus vaccine (see *A.3.1. Calibration Approach* for table of uncertain parameters). Previous mathematical models of CT transmission and other STIs were reviewed for consistency and to update parameters to reflect contemporary evidence. Fixed model parameters and sources are outlined in **Table S2**.

**Appendix A.3 Model Calibration & Uncertainty Assessment**

**A.3.1. Calibration Approach**

To perform model calibration, median adjusted case counts by demographic group and year (estimated above) were used. Although annual CT counts were available from 2000 to 2022, only data from 2013 to 2019 were included, due to uncertainty in early reporting of CT as a notifiable condition in the 2000s, as highlighted by Learner et al. (2020), and the widescale impacts of COVID-19 on disease reporting from 2020 onward.^14,41^ Model parameters subject to significant uncertainty were identified for model calibration and a plausible lower and upper bound were defined based on literature, prior models, and expert input (**Table S3**). The best fitting parameter set was identified by minimizing the sum of squared errors (SSEs) between simulated and observed data for the six demographic groups from 2013-2019. It was identified through an optimization routine using the Levenberg-Marquardt algorithm, a gradient-based method for nonlinear least squares optimization that combines features of both gradient descent and Gauss-Newton methods, which was run for 1000 steps to ensure convergence.^42^ Weighting was applied to prioritize better model fit for younger age groups and females, reflecting the higher CT burden in these populations.^8,11^ The SSE from the optimized parameter set served as a benchmark for evaluating fit in subsequent model runs.

Parameter uncertainty was incorporated into model calibration by generating 2,500 parameter sets uniformly sampled from a narrower sampling distribution informed by the best fitting parameter set from optimization. The model was run for all 2,500 sets, and the corresponding SSEs were calculated using the same weighting applied in the optimization. Parameter sets with SSE values falling within a predefined error tolerance level (2·75 times the optimized SSE) were retained. This process yielded a distribution of 100 plausible parameter sets that produced simulated outcomes consistent with observed surveillance data, thereby capturing uncertainty in parameter estimates beyond the single best-fitting set (**Figure S14**).

**A.3.2. Validation with NHANES Data**

Parameter sets from model calibration and uncertainty analyses were further fitted to sex-specific CT prevalence data from the U.S. CDC’s National Health and Nutrition Examination Survey (NHANES), collected biannually between 2009 and 2016.^56^ Due to lack of publicly available data for individuals aged 15 to 17 years, which requires additional data requests through the National Center for Health Statistics Research Data Center, only data for those aged 18 to 39 years were included in model validation. The model developed for this analysis includes individuals aged 15 to 17 years, who are at lower risk for CT acquisition compared to adults aged 18 years and older. As a result, model-based estimates of CT prevalence are expected to be lower than those observed in NHANES data that do not include 15 to 17-year-olds. To account for this discrepancy, model validation prioritized comparisons of temporal trends in CT prevalence rather than absolute prevalence estimates from 2009 to 2016.

**A.3.3. Results of Model Calibration**

The model fit was consistent with trends in adjusted CT incidence from CDC AtlasPlus between 2013 and 2019 across sex- and age-specific strata (**Figure S15**).^13^

The model also captured temporal trends in CT prevalence as reported by NHANES among 18 to 39-year-olds, although data for individuals aged 15 to 17 years were not available at the time of analysis due to removal of public-use data from executive orders (**Figure S16**).^56^ As expected, our estimates of prevalence were lower than those reported by NHANES, except among women captured in the 2015-2016 cycle. Together, these comparisons suggest that the model adequately reproduced key epidemiologic patterns of CT burden in the United States.

**Appendix B. Epidemiologic Outcomes**

**Appendix B.1. Referent and Intervention Scenarios (Base-Case)**

Referent and intervention scenarios used for to estimate epidemiologic outcomes are outlined in **Table S4**. All scenarios are compared to the referent scenario (no vaccination).

**Appendix B.2. Vaccination Scenarios**

All vaccination parameter values used for scenario analysis are outlined in **Table S5.**

**Appendix B.3. Epidemiologic Outcomes**

All formal definitions of primary epidemiologic outcomes are provided in **Table S6**.

**Appendix C. Costs, Health Utilities, and Cost-effectiveness Analyses**

**Appendix C.1. Health Outcomes and Cost Equations**

**C.1.1. Health Outcome Parameters, Equations, and Discounting**

Parameters associated with total quality-adjusted life years (QALYs) include:

- ${QALY}_{Symp}$ : QALY decrement associated with symptomatic CT infection
- ${QALY}_{Epid}$ : QALY decrement associated with a single case of epididymitis among males experiencing sequelae
- ${QALY}_{PID}$ : QALY decrement associated with a single case of PID among females experiencing sequelae, accounting for the likelihood of additional complications including chronic pelvic pain, tubal factor infertility, ectopic pregnancy, or no complications.
- *d* : Discount rate per year
- *y* : Year, between 2025 - 2050
- *ζ _j_* : Proportion of infections that present symptomatically, among individuals of sex *j*
- *ϕ* : Rate of transition among infected individuals from exposed, non-infectious to infectious
- *ι* : Rate of natural resolution among untreated symptomatic or asymptomatic infection
- *κ _j_* : Proportion of resolved symptomatic or asymptomatic infections that develop into sequelae, among individuals of sex *j*

Total QALYs were discounted at 3% over the 25-year time horizon using the following equation:

$$Total QALYs= \int_{y=1}^{25} e^{-d*y}\left( ({QALY}_{Symp}*\zeta_{j}\varphi*(E_{j,k}+E_{j,k}^{V}))+{(QALY}_{Epid}*\kappa_{j=1}\iota({Is}_{j=1,k}+{Ia}_{j=1,k}+{Is}_{j=1,k}^{V}+{Ia}_{j=1,k}^{V}))+ {QALY}_{PID}*\kappa_{j=2}\iota({Is}_{j=2,k}+{Ia}_{j=2,k}+{Is}_{j=2,k}^{V}+{Ia}_{j=2,k}^{V})) \right)$$

**C.1.2. Cost Outcome Parameters, Equations, and Discounting**

Parameters associated with total costs include:

- ${Cost}_{Screen}$ : Costs associated with 1.) screening among “true negative” individuals, 2.) combined screening/treatment among “false positive” individuals in the susceptible, exposed, and recovered compartments who receive unnecessary treatment, and 3.) screening among “false positive” individuals in the susceptible, exposed, and recovered compartments who do not receive unnecessary treatment. Importantly, these costs reflect screening in individuals who are truly negative only, calculated as:

${Cost}_{Screen}= {(r}_{j,k}*spec*screen\_cost *(S_{j,k}+E_{j,k}+R_{j,k}+S_{j,k}^{V}+E_{j,k}^{V}+R_{j,k}^{V}))+{(r}_{j,k}*(1-spec)*\upsilon* treat\_cost*(S_{j,k}+E_{j,k}+R_{j,k}+S_{j,k}^{V}+E_{j,k}^{V}+R_{j,k}^{V}))+ {(r}_{j,k}*(1-spec)*(1-\upsilon)* screen\_cost*(S_{j,k}+E_{j,k}+R_{j,k}+S_{j,k}^{V}+E_{j,k}^{V}+R_{j,k}^{V}))$, where:

- - *r _j,k_* : Screening rate for CT, among individuals of sex *j* and age group *k*
  - $spec$ : Specificity of diagnostic testing for CT detection via NAAT
  - $screen\_cost$ : Total direct and indirect costs associated with CT screening alone
  - $\upsilon$ : Probability of post-screening treatment among individuals following a positive NAAT result
  - $treat\_cost$ : Total direct and indirect costs associated with CT testing and treatment
  - $S_{j,k}+E_{j,k}+R_{j,k}+S_{j,k}^{V}+E_{j,k}^{V}+R_{j,k}^{V}$ : Uninfected population eligible for annual CT screening
- ${Cost}_{Asymp}$ : Costs associated with 1.) screening among asymptomatically infected “false negative” individuals, 2.) combined screening/treatment among asymptomatically infected “true positive” individuals, and 3.) screening among asymptomatically infected “true positive” individuals who do not receive treatment, calculated as:

${Cost}_{Asymp}= {(r}_{j,k}*(1- )*screen\_cost *({Ia}_{j,k}+{Ia}_{j,k}^{V}))+{(r}_{j,k}**\upsilon* treat\_cost*({Ia}_{j,k}+{Ia}_{j,k}^{V}))+ {(r}_{j,k}**(1-\upsilon)*screen\_cost*({Ia}_{j,k}+{Ia}_{j,k}^{V}))$, where:

- - *r _j,k_* : Screening rate for CT, among individuals of sex *j* and age group *k*
  - *ε* : Sensitivity of diagnostic testing for CT detection via NAAT
  - $screen\_cost$ : Total direct and indirect costs associated with CT screening alone
  - $\upsilon$ : Probability of post-screening treatment among individuals following a positive NAAT result
  - $treat\_cost$ : Total direct and indirect costs associated with CT testing and treatment
  - ${Ia}_{j,k}+{Ia}_{j,k}^{V}$ : Asymptomatically infected population eligible for annual CT screening
- ${Cost}_{Symp}$ : Costs associated with 1.) testing among symptomatically infected “false negative” individuals, 2.) combined testing/treatment among symptomatically infected “true positive” individuals, and 3.) testing among symptomatically infected “true positive” individuals who do not receive treatment, calculated as:

${Cost}_{Symp}=\left( {dur_{tx}}_{j}*\left( 1- \right)* screen_{cost}*\left( {Is}_{j,k}+{Is}_{j,k}^{V} \right) \right)+ \left( {dur_{tx}}_{j}* *p_{txt}*treat_{cost}*\left( {Is}_{j,k}+{Is}_{j,k}^{V} \right) \right)+\left( {dur_{tx}}_{j}* *(1-p_{txt})*screen_{cost}*\left( {Is}_{j,k}+{Is}_{j,k}^{V} \right) \right)$, where:

- - ${dur\_tx}_{j}$ : Duration of treated infection, among individuals of sex *j*
  - *ε* : Sensitivity of diagnostic testing for CT detection via NAAT
  - *p_txt* : Probability of treatment for symptomatic infection, given positive diagnosis
  - $treat\_cost$ : Total direct and indirect costs associated with CT testing and treatment
  - $screen\_cost$ : Total direct and indirect costs associated with CT screening alone
  - ${Is}_{j,k}+{Is}_{j,k}^{V}$ : Total symptomatically infected population
- ${Cost}_{Epid}$ : Costs associated with treatment of Epididymitis in males with untreated CT infection, calculated as:

${Cost}_{Epid}= epid\_costs *\varpi_{j=1}(Q_{j=1,k}+Q_{j=1,k}^{V})$, where:

- - $epid\_costs$: Total direct and indirect costs associated with treatment of epididymitis in males with untreated CT infection
  - *ϖ _j_*_=1_ : Rate of sequelae resolution, among males
  - ${Is}_{j=1,k}+{Ia}_{j=1,k}+{Is}_{j=1,k}^{V}+{Ia}_{j=1,k}^{V}$ : Total infected population, inclusive of symptomatic and asymptomatic infections among males
  - $Q_{j=1,k}+Q_{j=1,k}^{V}$ : Total population experiencing epididymitis
- ${Cost}_{PID}$ : Costs associated with treatment of PID and subsequent complications (chronic pelvic pain, ectopic pregnancy, tubal factor infertility, or no complications) among females with untreated CT infection, calculated as:

${Cost}_{PID}= pid\_costs *\varpi_{j=2}(Q_{j=2,k}+Q_{j=2,k}^{V})$, where:

- - $pid\_costs$ : Total direct and indirect costs associated with treatment of PID (and subsequent complications) in females with untreated CT infection
  - *ϖ _j_*_=2_ : Rate of sequelae resolution, among females
  - $Q_{j=2,k}+Q_{j=2,k}^{V}$ : Total population experiencing PID and associated complications
- ${Cost}_{Vx\_Dose}$: Costs of CT vaccination (only price per dose), calculated as:

${Cost}_{Vx\_Dose}=\left( dose\_cost*{Vx}_{j}{\eta N}_{j} \right)+\left( dose\_cost*\beta_{j,k}\left( S_{j,k}+E_{j,k}+{Ia}_{j,k}+R_{j,k} \right) \right)$, where:

- - $dose\_cost$ : Total cost of CT vaccination doses
  - *Vx _j_* : Proportion of individuals of sex *j* vaccinated upon entering the population at age 15 years
  - *η* : Relative size of the population entering the model at age 15, dependent on time *t*
  - *N _j_* : Total size of the simulated population, among individuals of sex *j* dependent on time *t*
  - *β _j,k_* : Rate of vaccination coverage via catch-up campaigns, among individuals of sex *j* and age group *k*
  - $S_{j,k}+E_{j,k}+{Ia}_{j,k}+R_{j,k}$ : Total population eligible for vaccination via catch-up campaigns
- ${Cost}_{Vx\_Imp}$ : Costs of CT vaccination implementation (excluding price per dose), calculated as:

${Cost}_{Vx\_Imp}=(vximp\_costs *{Vx}_{j}{\eta N}_{j})+(vximp\_costs *\beta_{j,k}(S_{j,k}+E_{j,k}+{Ia}_{j,k}+R_{j,k}))$, where:

- - $vximp\_costs$ : Total direct and indirect costs associated with CT vaccination implementation
  - *Vx _j_* : Proportion of individuals of sex *j* vaccinated upon entering the population at age 15 years
  - *η* : Relative size of the population entering the model at age 15, dependent on time *t*
  - *N _j_* : Total size of the simulated population, among individuals of sex *j* dependent on time *t*
  - *β _j,k_* : Rate of vaccination coverage via catch-up campaigns, among individuals of sex *j* and age group *k*
  - $S_{j,k}+E_{j,k}+{Ia}_{j,k}+R_{j,k}$ : Total population eligible for vaccination via catch-up campaigns

Total costs were discounted at 3% over the 25-year time horizon using the following equation:

$$Total Costs= \int_{y=1}^{25} e^{-d*y}\left( {Cost}_{Screen}+ {Cost}_{Asymp}+ {Cost}_{Symp}+ {Cost}_{Epid}+{Cost}_{PID}+{Cost}_{Vx\_Dose}+{Cost}_{Vx\_Imp} \right)$$

**C.1.3. Outline of Model-specific Parameters for Health and Cost Outcome Equations**

Details on model-specific parameters and sources are summarized in **Table S7**.

**Appendix C.2. QALY and Cost Estimates**

**C.2.1. Cost Estimates**

Costs and corresponding sources are available in **Table S8**.

**C.2.2. Health Utility (QALY) Estimates**

QALY utilities and corresponding sources are available in **Table S9**.

**Appendix C.3. Referent and Intervention Scenarios**

Referent and intervention scenarios used for the cost-effectiveness analysis are outlined in **Table S10**.

**Appendix C.4. ICER Estimation**

We calculated ICERs as the additional cost per additional QALY gained when moving from one vaccination strategy to a more extensive strategy:

$$ICER= \frac{{Cost}_{Intervention}-{Cost}_{Referent}}{{QALYs}_{Intervention}- {QALYs}_{Referent}}$$

Depending on the comparison, the ICER either reflects the incremental cost-effectiveness of introducing vaccination compared to the current standard of care (screen-and-treat and test-and-treat) or the incremental cost-effectiveness of expanding vaccination beyond a simpler program (see **Table S11**). We evaluated ICERs over a time horizon from 2025 to 2050 and interpreted ICERs to be very cost-effective if they fell below the willingness-to-pay threshold (WTP) of $50,000 to $100,000 USD per QALY gained ($/QALY).^70^ Although WTP values for the U.S. have been cited up to $150,000 USD, we have taken a conservative approach to reflect potential bias against sexual and reproductive health services.^71^ As our primary approach, we conducted a sequential analysis separately for the female-only and sex-neutral routine vaccination pathways.

**Appendix C.5. Scenario Analysis**

The base-case scenario for Groups A, B, C, and D assumed 50% vaccine coverage, attained linearly by 2035 from 0% in 2025 among 15-year-olds, 10-year duration of protection, and 70% VE. The base-case scenario for Groups C and D also assumed 10% of the unvaccinated 15-24-year-old female population was vaccinated via catch-up campaigns in 2035. We conducted an extensive scenario analysis, in which we simulated variations of Scenario Groups A and B to assess the impacts of varying relevant vaccination parameters on reported results as follows:

1. To evaluate how VE estimates influence ICERs across the time horizon, we applied the base-case scenario and varied VE at 30%, 50%, or 90%, respectively.
2. To evaluate how vaccination uptake influenced ICERs across the time horizon, we applied the base-case scenario and varied vaccination coverage among 15-year-olds at lower (30%) or higher (70%) coverage levels by 2035, respectively.
3. To evaluate how vaccine-conferred immunity influenced ICERs across the time horizon, we applied the base-case scenario and varied duration of protection at 5 or 20 years, respectively.

A similar approach was used for Scenario Groups C and D with coverage among 15-year-olds fixed at 50% by 2035 and focusing on varying coverage from catch-up campaigns (base-case = 10% vs. 5% or 15%). A descriptive summary of all scenarios can be found in **Table S12**.

**Appendix C.6. Sensitivity Analysis**

To further examine the robustness of our findings, we conducted one-way sensitivity analyses by individually varying the price per vaccine dose, treatment and prevention costs, QALY estimates, and discounting rates using feasible ranges informed by published studies. Model calibration incorporated key epidemiological uncertainties for several parameters, including the probability of symptomatic presentation and likelihood of sequalae development. As such, these parameters were not separately varied in one-way sensitivity analyses. All sensitivity analyses assume the base-case scenario (Scenarios A-D: 50% coverage by 2035, 10-year duration of protection, and 70% VE; Scenarios C/D: 10% catch-up campaign coverage in 2035).

**Supplementary Results**

**Appendix D. Epidemiologic Outcomes**

**Appendix D.1. Scenario Analysis - Absolute and Relative Reductions in CT Infections**

Results from scenario analysis are provided in **Figure S17** (Scenarios A and B) and **Figure S18** (Scenarios C and D). Additional detail for all scenarios is provided in **Tables S13-S16**.

**Appendix D.2. Scenario Analysis – Reductions in CT Incidence Rates**

Results from base-case assumptions are provided in **Figure S19** (Scenarios A) and **Figure S20** (Scenario B). Additional detail for all scenarios provided in **Tables S17-S20**. Additional results for sensitivity analysis comparing incidence rates under equal screening rate assumptions for males and females by age are provided in **Table S21.**

**Appendix D.3. Scenario Analysis – Vaccination Efficiency**

Results from scenario analysis are provided in **Figure S21** (Scenarios A and B) and **Figure S22** (Scenarios C and D). Additional detail for all scenarios are provided in **Table**s **S22-S25**.

**Appendix D.4. Secondary Analysis – Sequalae Prevented (Base-case)**

Under base-case assumptions, we estimated the cumulative and proportional reductions in sequelae events, including pelvic inflammatory disease, tubal factor infertility, ectopic pregnancy, chronic pelvic pain, and epididymitis, by 2075 comparing all vaccination strategies (Scenarios A-D) to no vaccination (Referent Scenario). Results are provided in **Table S26**.

**Appendix E. Costs, Health Utilities, and Cost-effectiveness Analyses**

**Appendix E.1. Scenario Analysis**

Results from scenario analysis are presented in **Tables S27-S30** and **Figures S23-25**.

**Appendix E.2. Sensitivity Analysis**

Tornado plots of one-way sensitivity analyses under base case-assumptions are presented in **Figures S26-29**.

**Appendix F. Consolidated Health Economic Evaluation**

**Reporting Standards (CHEERS) 2022 Checklist**

| **Section/Topic** | **Item No.** | **Guidance for Reporting** | **Reported in section** |
| --- | --- | --- | --- |
| **TITLE** | | | |
| Title | 1 | Identify the study as an economic evaluation and specify the interventions being compared. | Title, Introduction |
| **ABSTRACT** | | | |
| Abstract | 2 | Provide a structured summary that highlights context, key methods, results and alternative analyses. | Abstract |
| **INTRODUCTION** | | | |
| Background and objectives | 3 | Give the context for the study, the study question and its practical relevance for decision making in policy or practice. | Introduction |
| **METHODS** | | | |
| Health economic analysis plan | 4 | Indicate whether a health economic analysis plan was developed and where available. | No standalone health economic analysis plan was prepared; instead, the analytic approach was designed in parallel with the model. |
| Study population | 5 | Describe characteristics of the study population (such as age range, demographics, socioeconomic, or clinical characteristics). | Methods, Paragraphs 1 & 2 |
| Setting and location | 6 | Provide relevant contextual information that may influence findings. | Methods, Paragraph 1 |
| Comparators | 7 | Describe the interventions or strategies being compared and why chosen. | Methods, Paragraphs 3 & 4 |
| Perspective | 8 | State the perspective(s) adopted by the study and why chosen. | Methods, Paragraph 6 |
| Time horizon | 9 | State the time horizon for the study and why appropriate. | Methods, Paragraph 7 |
| Discount rate | 10 | Report the discount rate(s) and reason chosen. | Methods, Paragraph 7 |
| Selection of outcomes | 11 | Describe what outcomes were used as the measure(s) of benefit(s) and harm(s). | Methods, Paragraph 8 |
| Measurement of outcomes | 12 | Describe how outcomes used to capture benefit(s) and harm(s) were measured. | Methods, Paragraph 8; Supplementary Material |
| Valuation of outcomes | 13 | Describe the population and methods used to measure and value outcomes. | Methods, Paragraph 8; Supplementary Material |
| Measurement and valuation of resources and costs | 14 | Describe how costs were valued. | Methods, Paragraph 6; Supplementary Material |
| Currency, price date, and conversion | 15 | Report the dates of the estimated resource quantities and unit costs, plus the currency and year of conversion. | Methods, Paragraphs 6 & 7 |
| Rationale and description of model | 16 | If modelling is used, describe in detail and why used. Report if the model is publicly available and where it can be accessed. | Methods, Paragraphs 1 & 2; Supplementary Material |
| Analytics and assumptions | 17 | Describe any methods for analyzing or statistically transforming data, any extrapolation methods, and approaches for validating any model used. | Methods, Paragraphs 1 & 2; Supplementary Material |
| Characterizing heterogeneity | 18 | Describe any methods used for estimating how the results of the study vary for sub-groups. | Methods, Paragraph 9; Supplementary Material |
| Characterizing distributional effects | 19 | Describe how impacts are distributed across different individuals or adjustments made to reflect priority populations. | No specific adjustments made. |
| Characterizing uncertainty | 20 | Describe methods to characterize any sources of uncertainty in the analysis. | Methods, Paragraph 10, Supplementary Material |
| Approach to engagement with patients and others affected by the study | 21 | Describe any approaches to engage patients or service recipients, the general public, communities, or stakeholders (e.g., clinicians or payers) in the design of the study. | Not applicable. |
| **RESULTS** | | | |
| Study parameters | 22 | Report all analytic inputs (e.g., values, ranges, references) including uncertainty or distributional assumptions. | Table 2; Supplementary Material |
| Summary of main results | 23 | Report the mean values for the main categories of costs and outcomes of interest and summarize them in the most appropriate overall measure. | Results; Paragraphs 5 & 6 |
| Effect of uncertainty | 24 | Describe how uncertainty about analytic judgments, inputs, or projections affect findings. Report the effect of choice of discount rate and time horizon, if applicable. | Results, Paragraphs 7 & 8; Supplementary Material |
| Effect of engagement with patients and others affected by the study | 25 | Report on any difference patient/service recipient, general public, community, or stakeholder involvement made to the approach or findings of the study. | Not applicable. |
| **DISCUSSION** | | | |
| Study findings, limitations, generalizability, and current knowledge | 26 | Report key findings, limitations, ethical or equity considerations not captured, and how these could impact patients, policy, or practice. | Discussion |
| **OTHER RELEVANT INFORMATION** | | | |
| Source of funding | 27 | Describe how the study was funded and any role of the funder in the identification, design, conduct, and reporting of the analysis | Funding disclosure |
| Conflicts of interest | 28 | Report authors conflicts of interest according to journal or International Committee of Medical Journal Editors requirements. | Conflict of interest disclosure |

From: Husereau D, Drummond M, Augustovski F, de Bekker-Grob E, Briggs AH, Carswell C, Caulley L, Chaiyakunapruk N, Greenberg D, Loder E, Mauskopf J, Mullins CD, Petrou S, Pwu RF, Staniszewska S; CHEERS 2022 ISPOR Good Research Practices Task Force. Consolidated Health Economic Evaluation Reporting Standards 2022 (CHEERS 2022) Statement: Updated Reporting Guidance for Health Economic Evaluations. BMJ. 2022;376:e067975.

**Supplementary Figures**

**
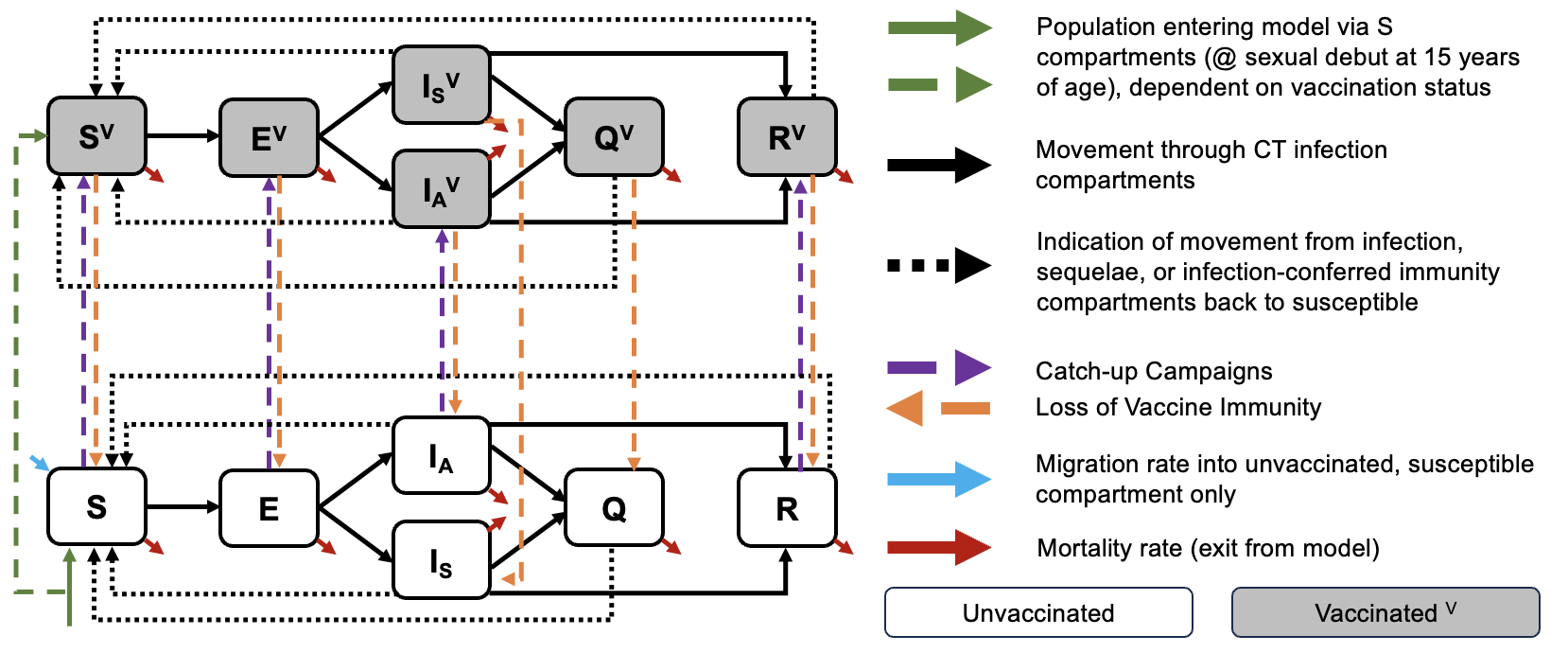
**

**Figure S1. *Chlamydia trachomatis* (CT) transmission model.** Simulated population is stratified by CT infection status and progression towards sequelae: susceptible (S), exposed non-infectious (E), infectious symptomatic infection (I_S_), infectious asymptomatic infection (I_A_), sequelae-experiencing (Q), or infection-conferred immunity (R). The population is further stratified by age (15-24, 25-39, 40-64 years), biological sex assigned at birth (males, females), and vaccination status (^V^).

**
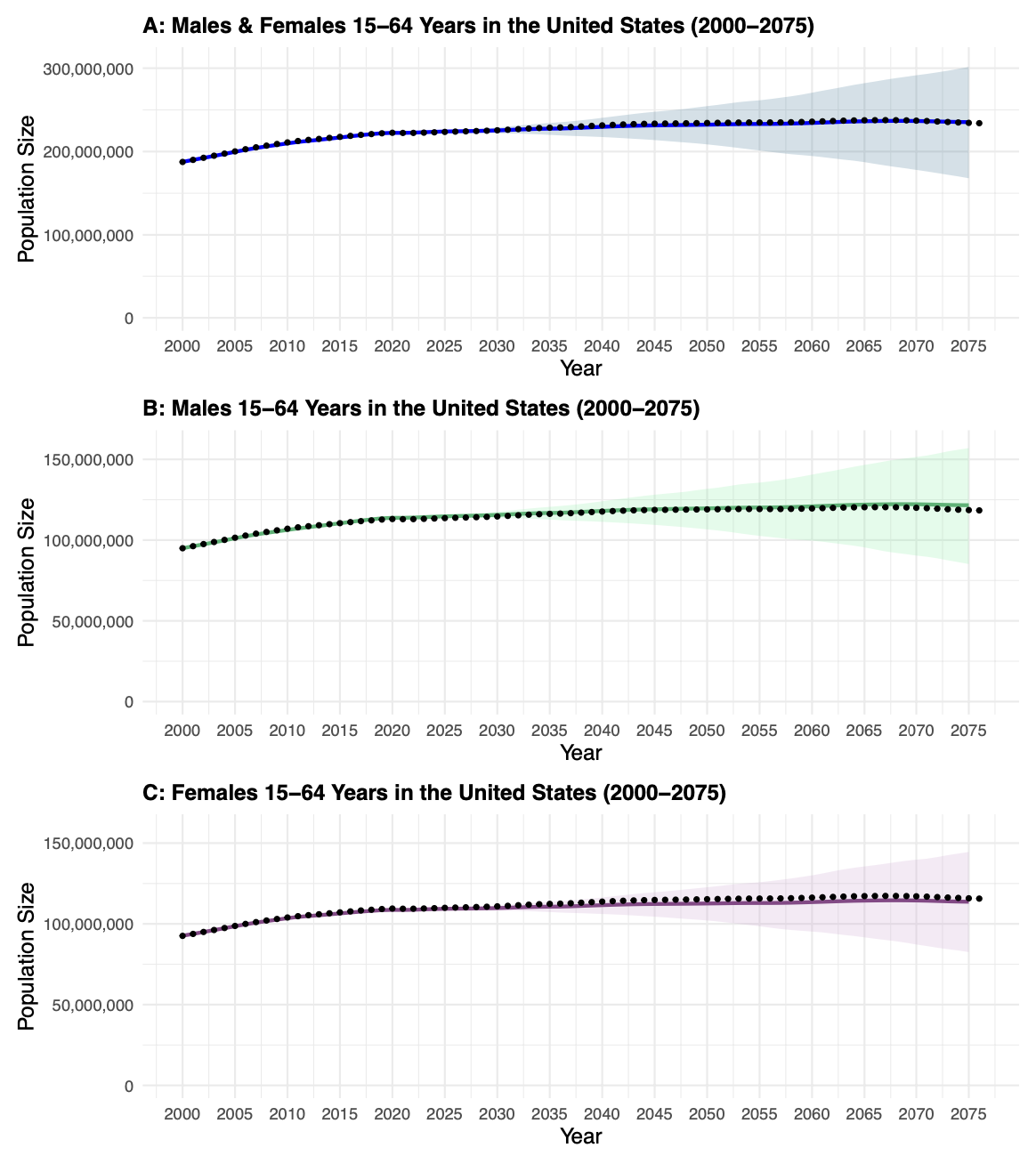
**

**Figure S2.** Comparison of United Nations population projections (solid lines; confidence intervals) from 2000 to 2075 versus model-generated annual demographic estimates (dotted lines)

**
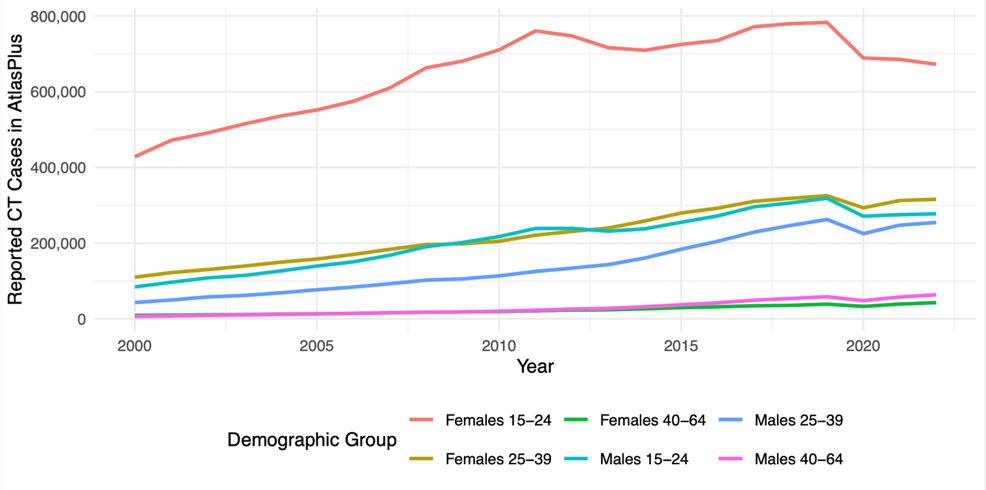
**

**Figure S3.** Annual AtlasPlus CT cases reported, by demographic group


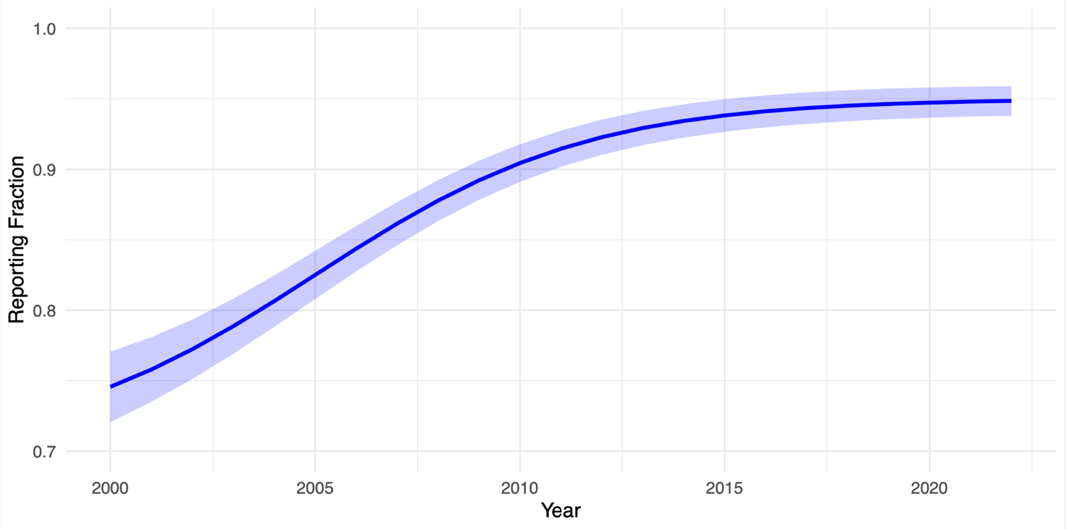


**Figure S4.** Estimated reporting fraction by year in the United States


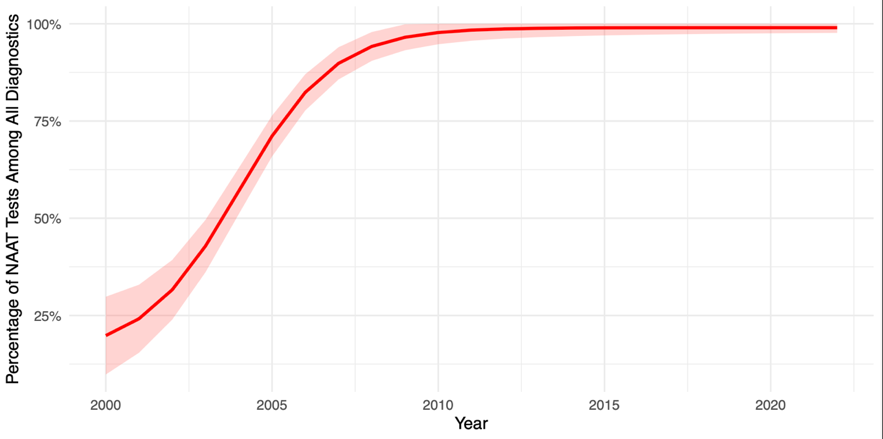


**Figure S5.** Proportion of NAAT tests used by year


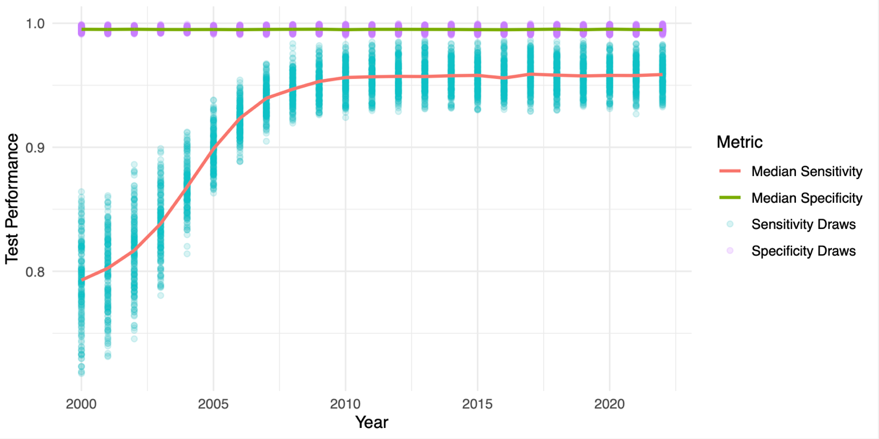


**Figure S6.** Weighted sensitivity and specificity of diagnostic tests by year


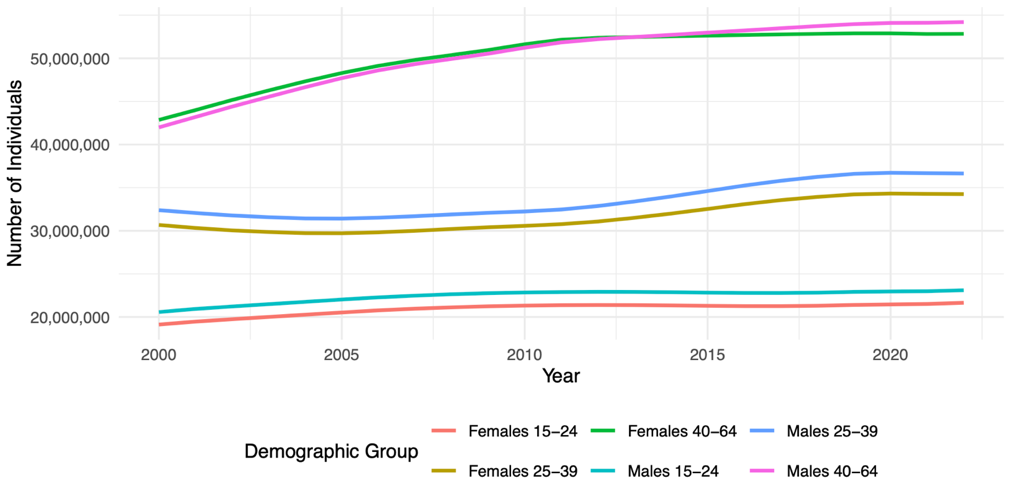


**Figure S7.** Annual U.S. population data from the U.N., by demographic group and year


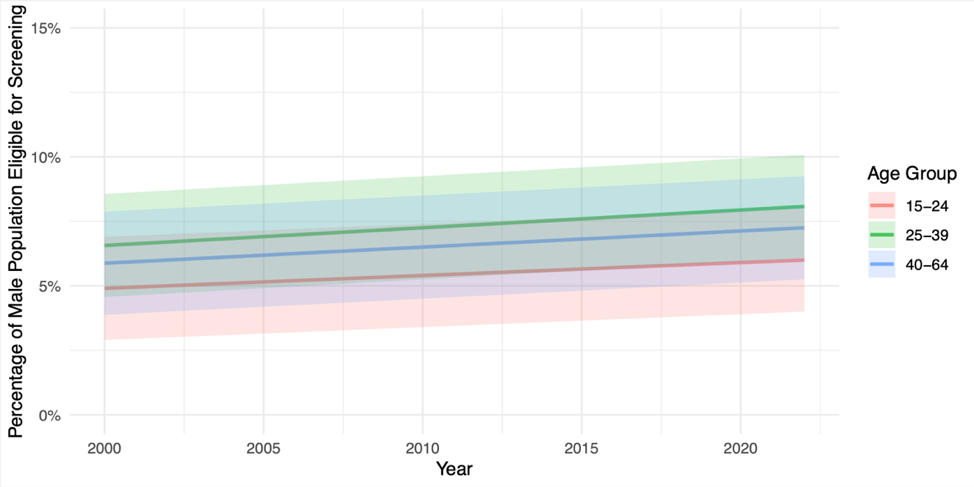


**Figure S8.** Proportion of male population eligible for screening, by age group and year


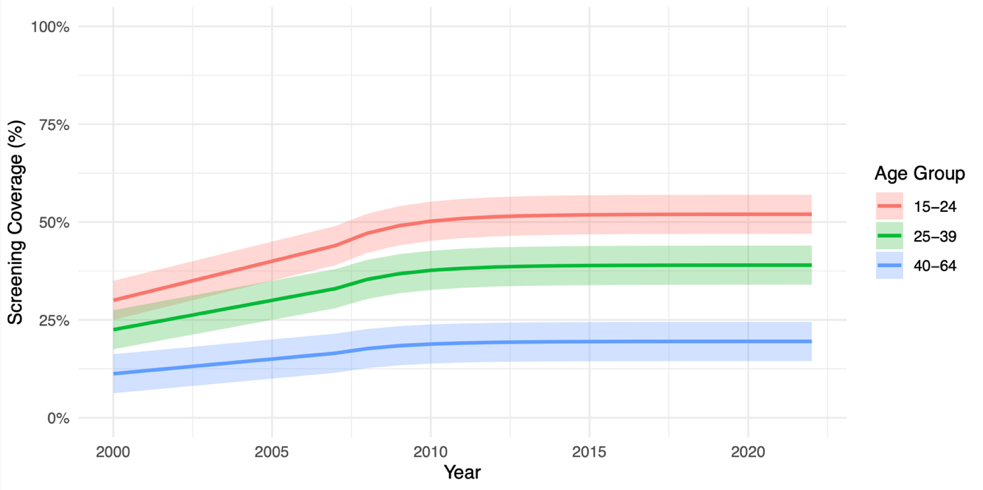


**Figure S9.** Screening coverage among eligible females, by age group and year


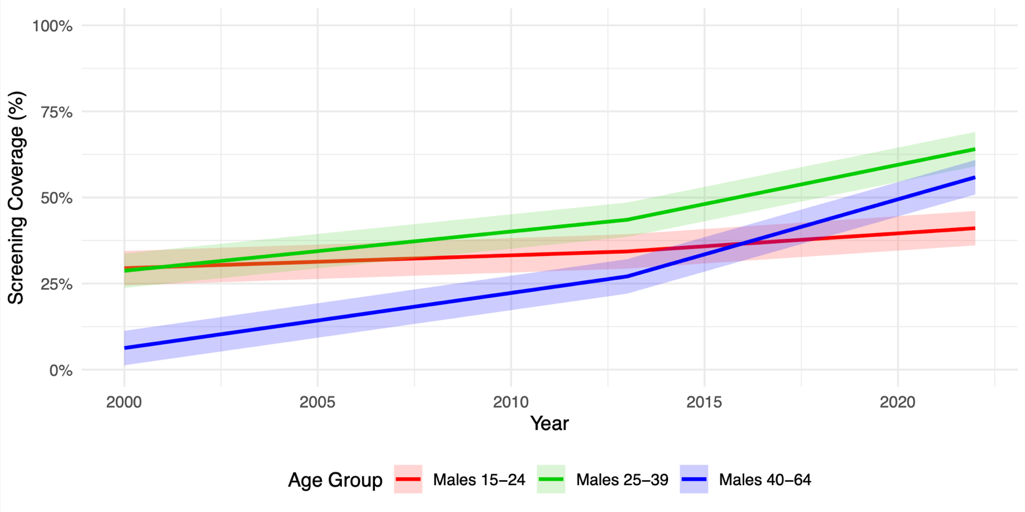


**Figure S10.** Screening coverage among eligible males, by age group and year


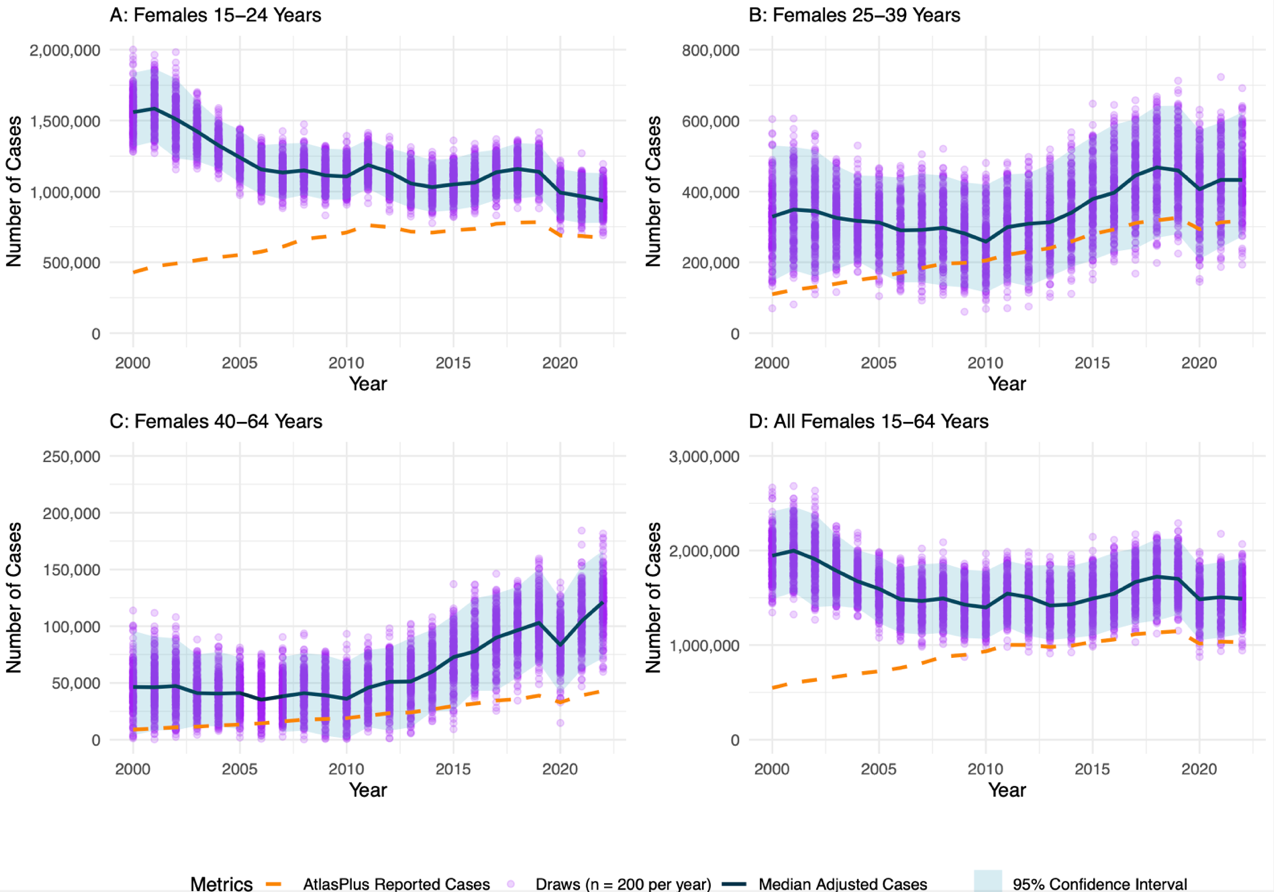


**Figure S11.** Adjusted versus reported AtlasPlus case by year in the United States, among females


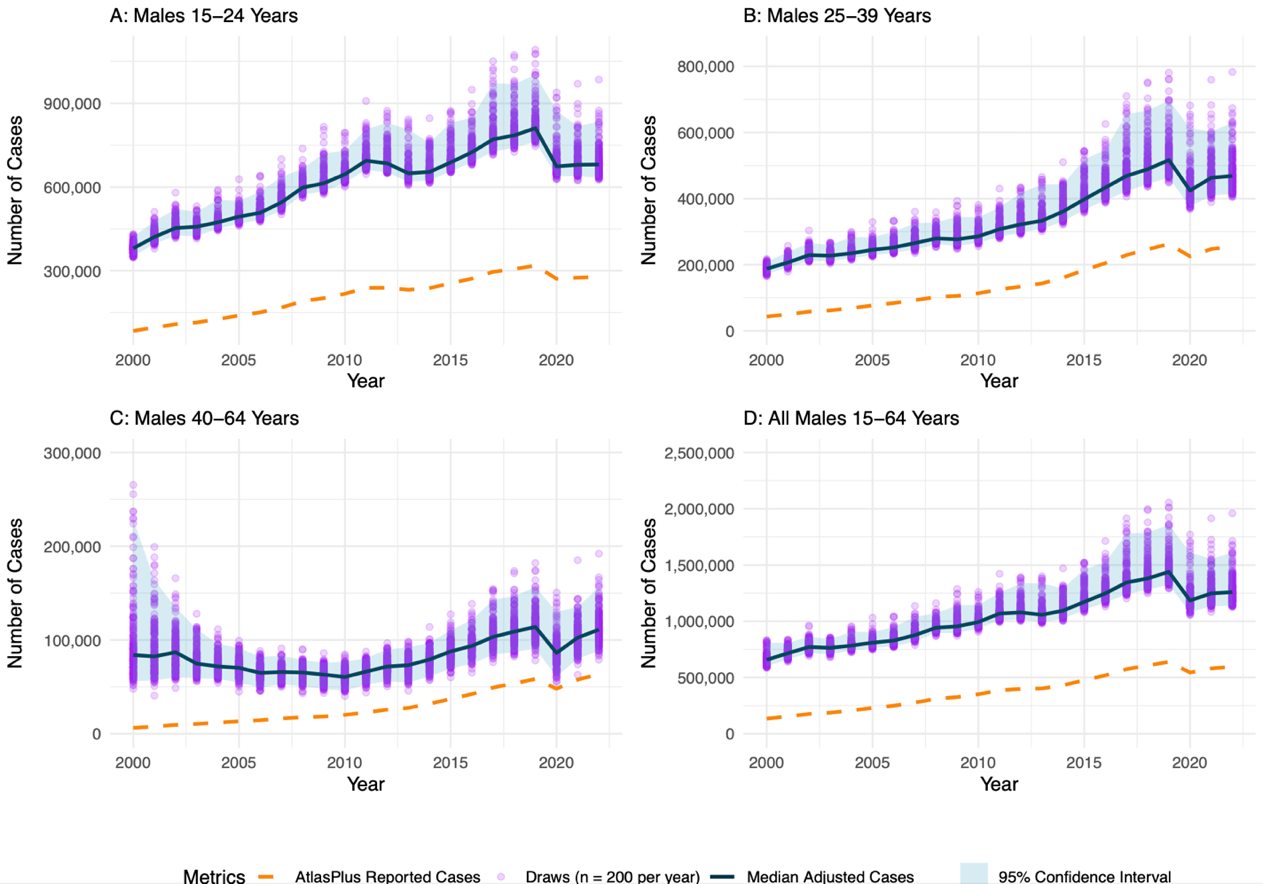


**Figure S12.** Adjusted versus reported AtlasPlus case by year in the United States, among males


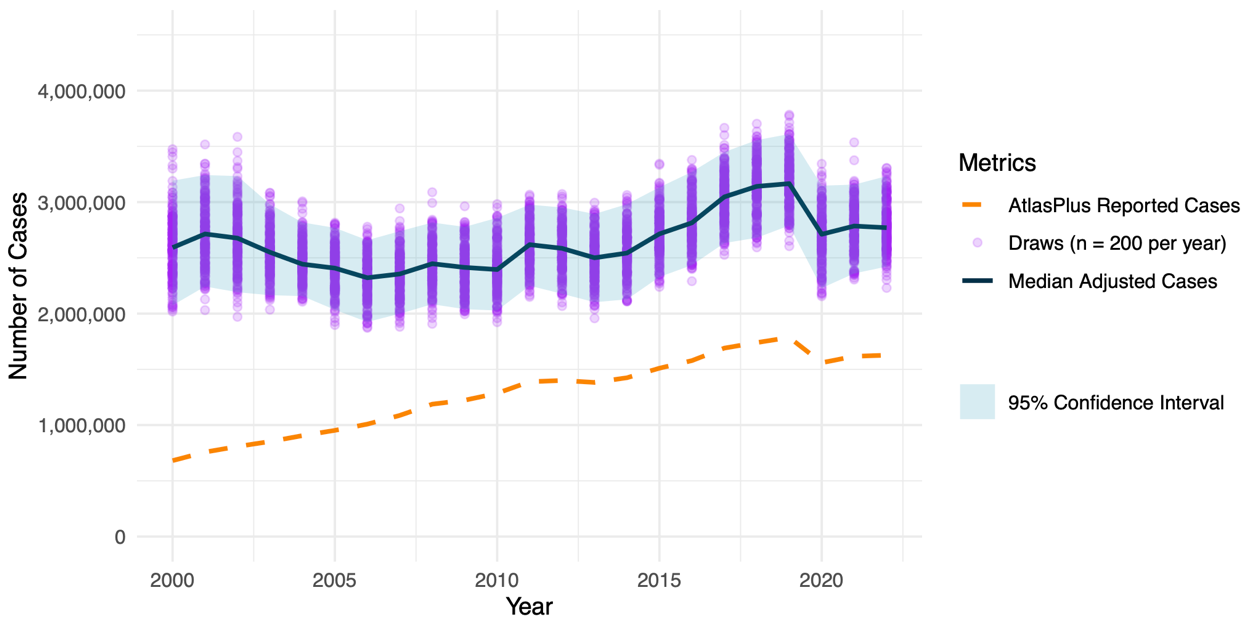


**Figure S13.** Adjusted versus reported AtlasPlus cases by year in the United States, among all males and females aged 15-64 years

**
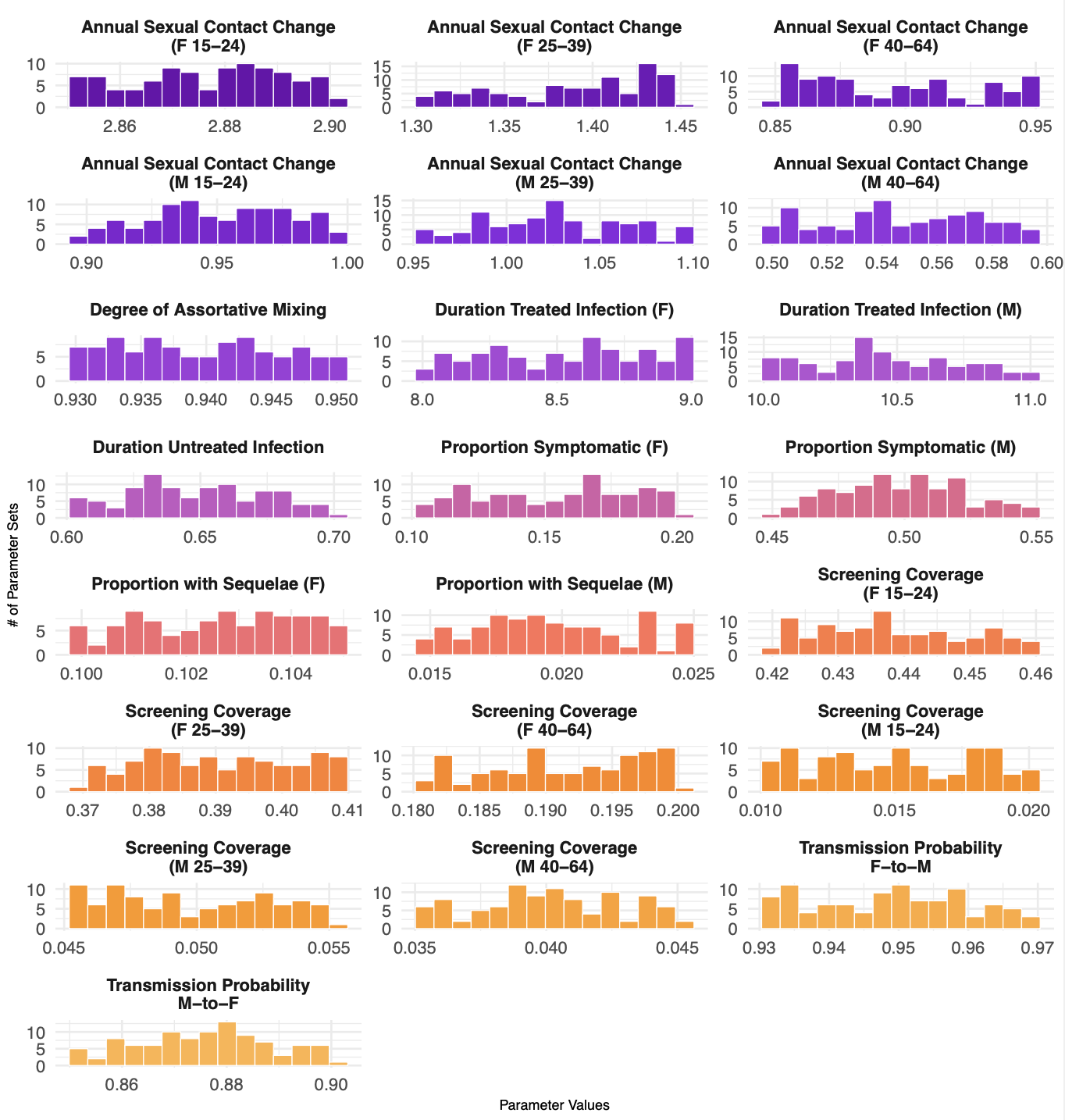
**

**Figure S14.** Distribution of uncertain parameter values based on parameter sets accepted from model calibration (N = 100)

**
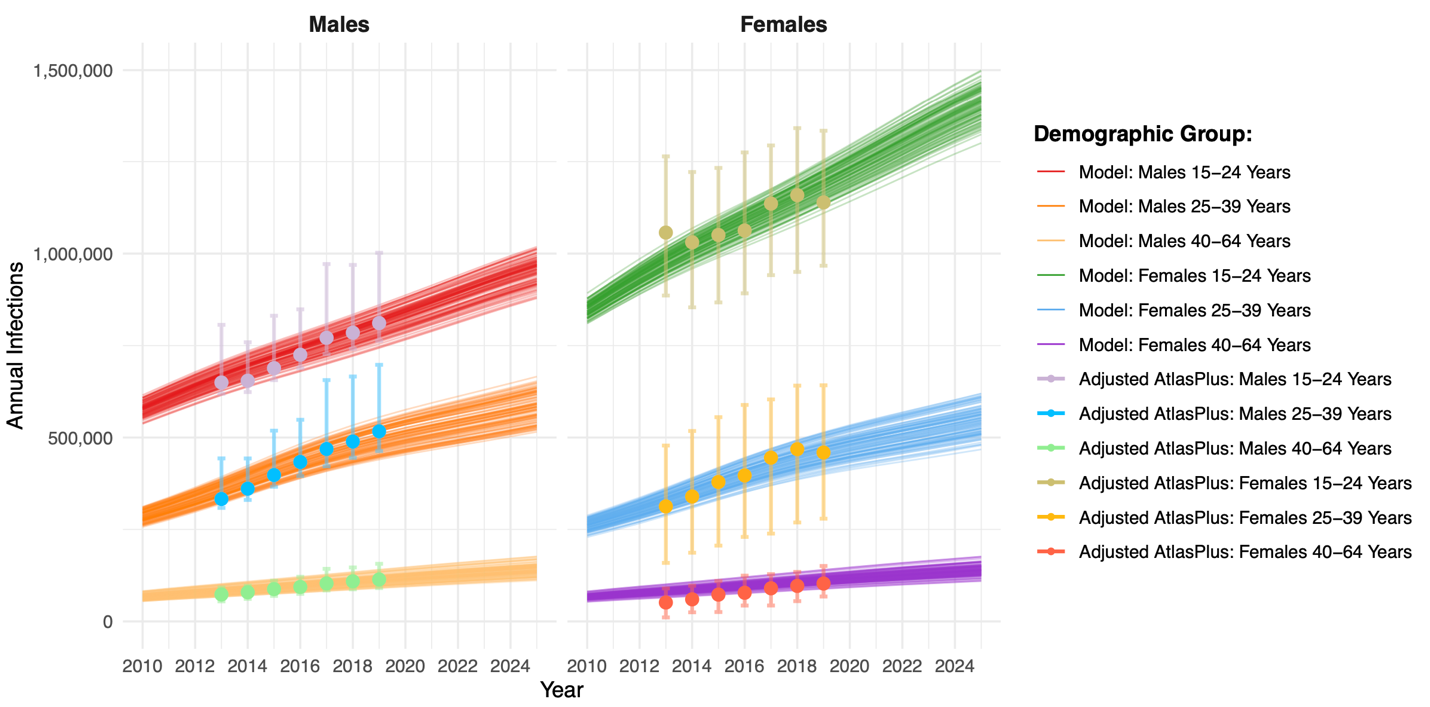
**

**Figure S15**. Assessment of model fit, comparing annual incident cases of CT from model simulations using 100 parameter sets accepted from model calibration (solid lines) vs. adjusted AtlasPlus incident cases of CT (dots and confidence intervals), by demographic group and year


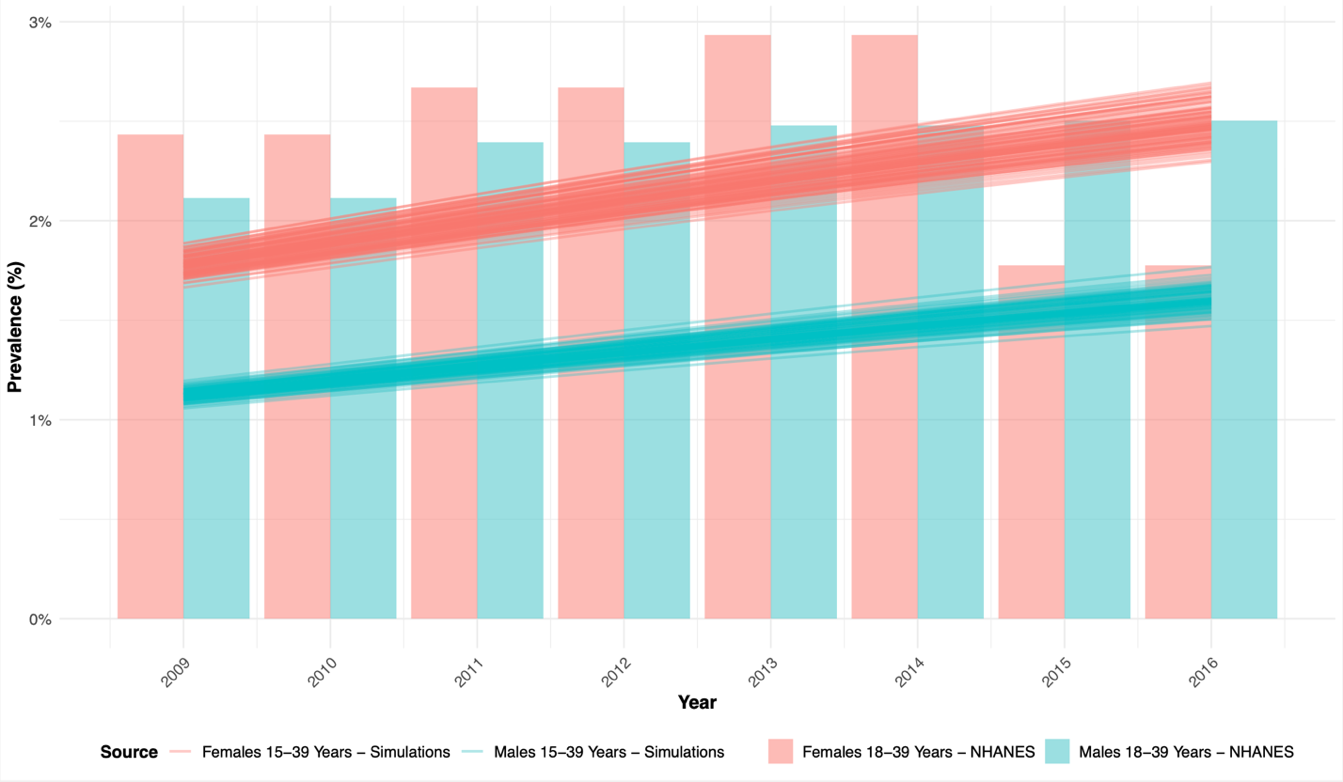


**Figure S16**. Assessment of model fit, comparing annual CT prevalence estimates from model simulations (solid lines; ages 15–39 years) vs. NHANES data (bars; ages 18–39 years), by biological sex and year


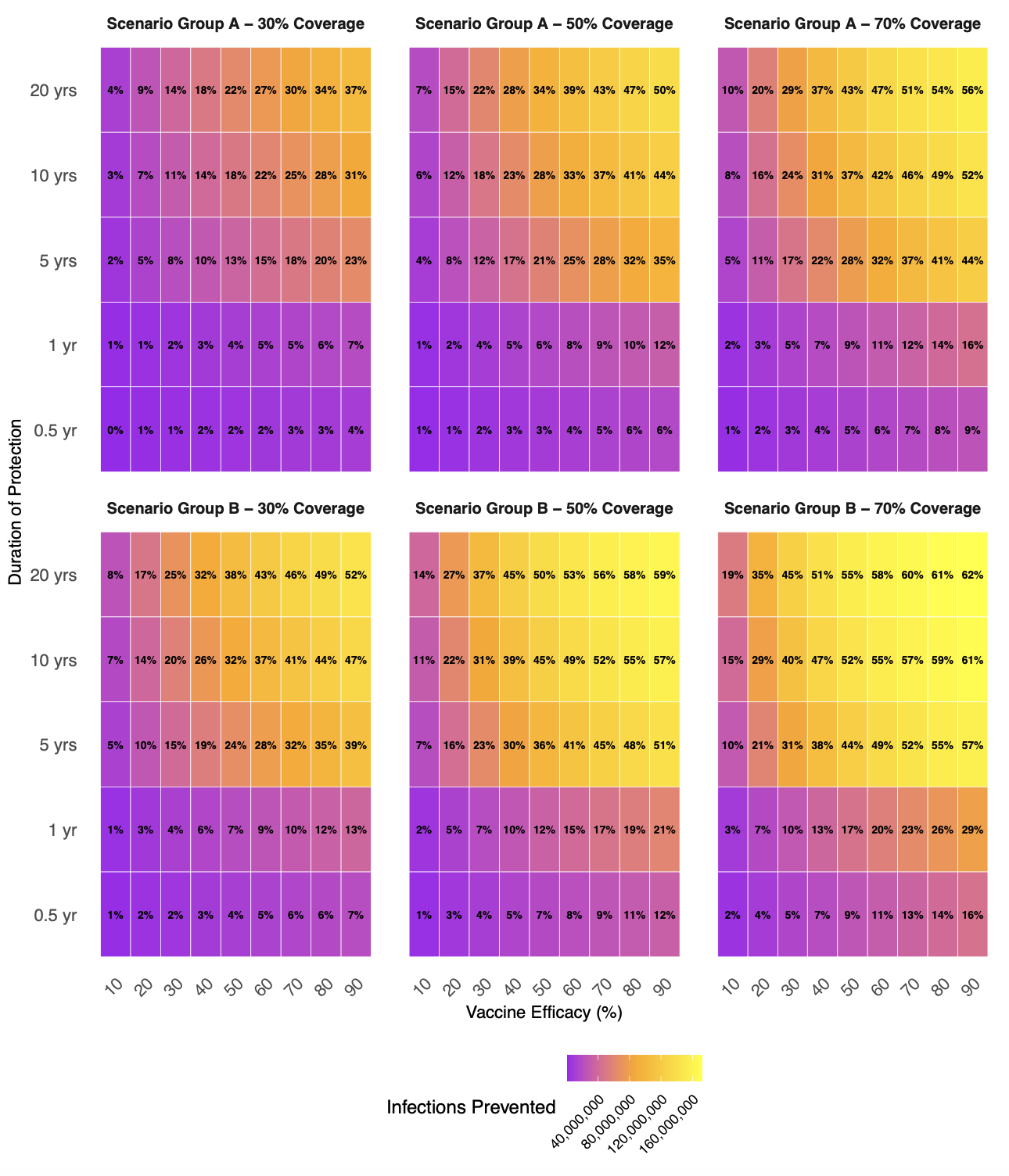


**Figure S17.** Cumulative CT infections prevented among males and females aged 15 to 64 years between 2025-2075, across combinations of vaccine coverage, efficacy, and duration of protection, for Scenario Groups A and B compared to no vaccination (referent scenario). Heatmap: Cumulative infections prevented represented by colors. Text Values: Relative reduction in CT infections. Scenario A: Vaccinating 15-year-old females entering the model between 2025-2075. Scenario B: Vaccinating 15-year-old females and males entering the model between 2025-2075.


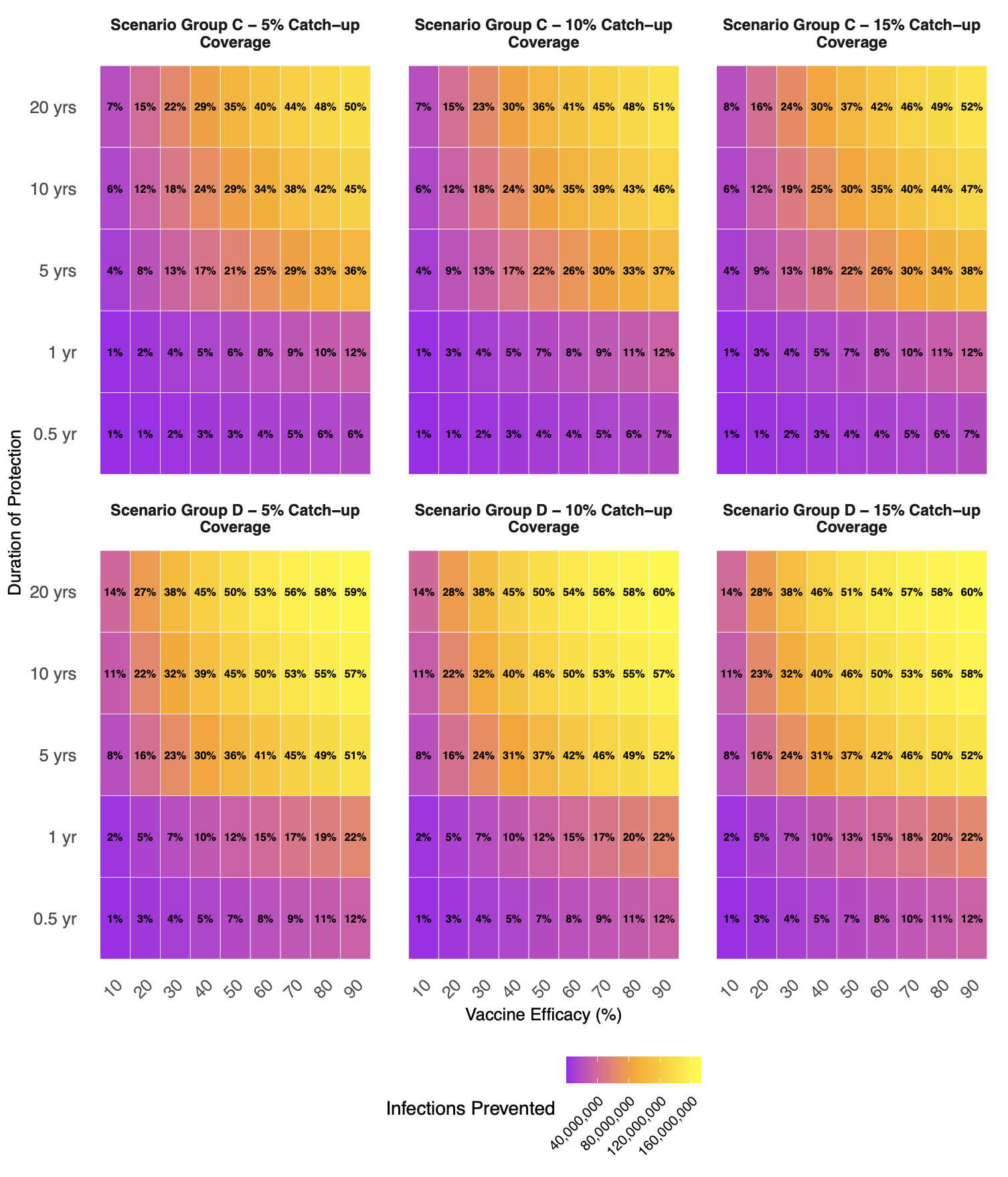


**Figure S18.** Cumulative CT infections prevented among males and females aged 15 to 64 years between 2025-2075, across combinations of catch-up vaccination coverage, efficacy, and duration of protection, for Scenario Groups C and D compared to no vaccination (referent scenario). Heatmap: Cumulative infections prevented represented by colors. Text Values: Relative reduction in CT infections. Scenario C: Scenario A at 50% coverage + catch-up campaign among unvaccinated 15-24-year-old females in 2035. Scenario D: Scenario B at 50% coverage + catch-up campaign among unvaccinated 15-24-year-old females in 2035.


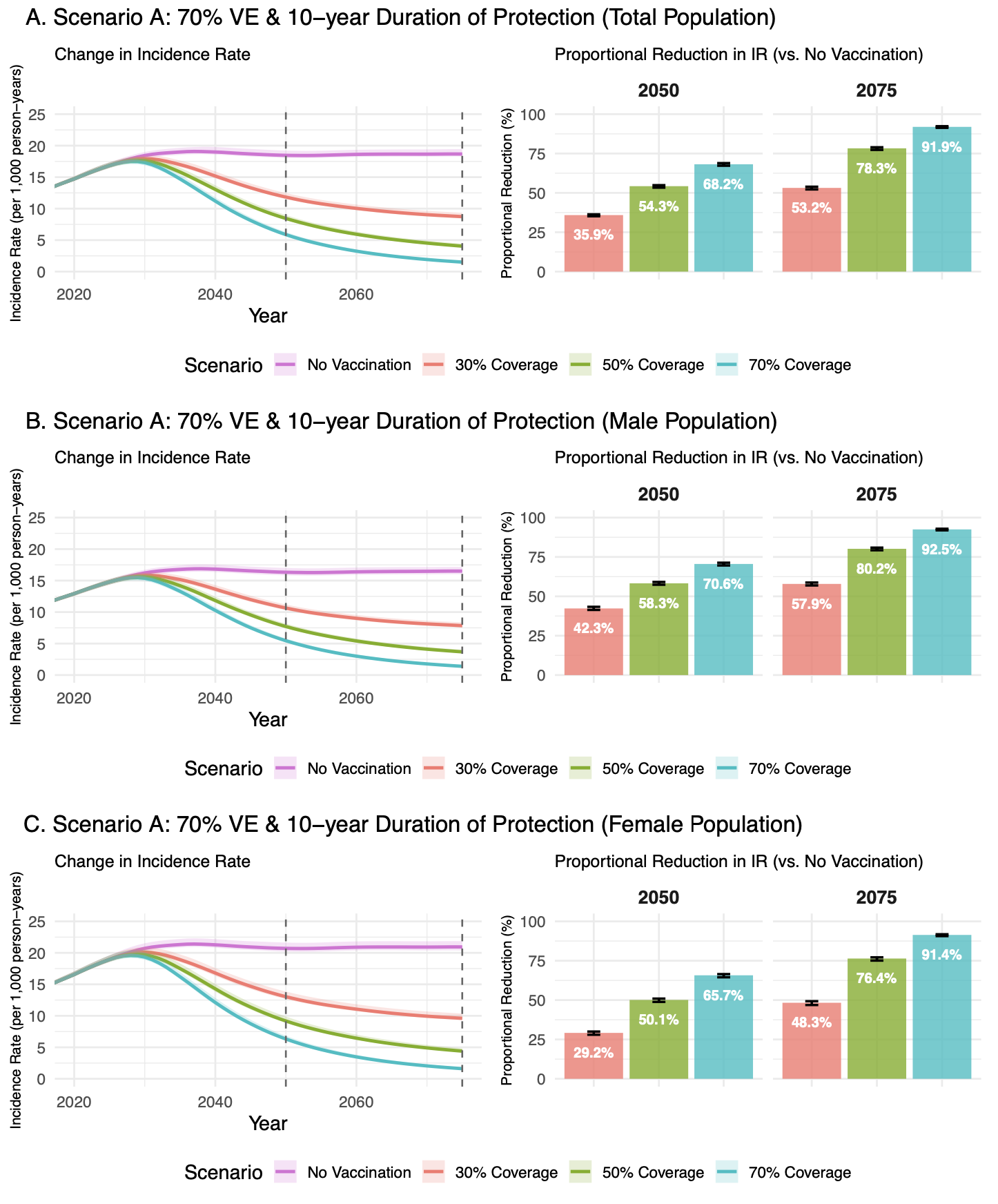


**Figure S19.** The absolute and proportional reduction in CT incidence rates for Scenario A in 2050 and 2075 when compared to the Referent Scenario (no vaccination), assuming 10-year duration of vaccine-conferred immunity and 70% VE (base-case assumption). Results are presented by vaccine coverage attained by 2035 and biological sex. CT incidence rates estimated among individuals aged 15 to 64 years.


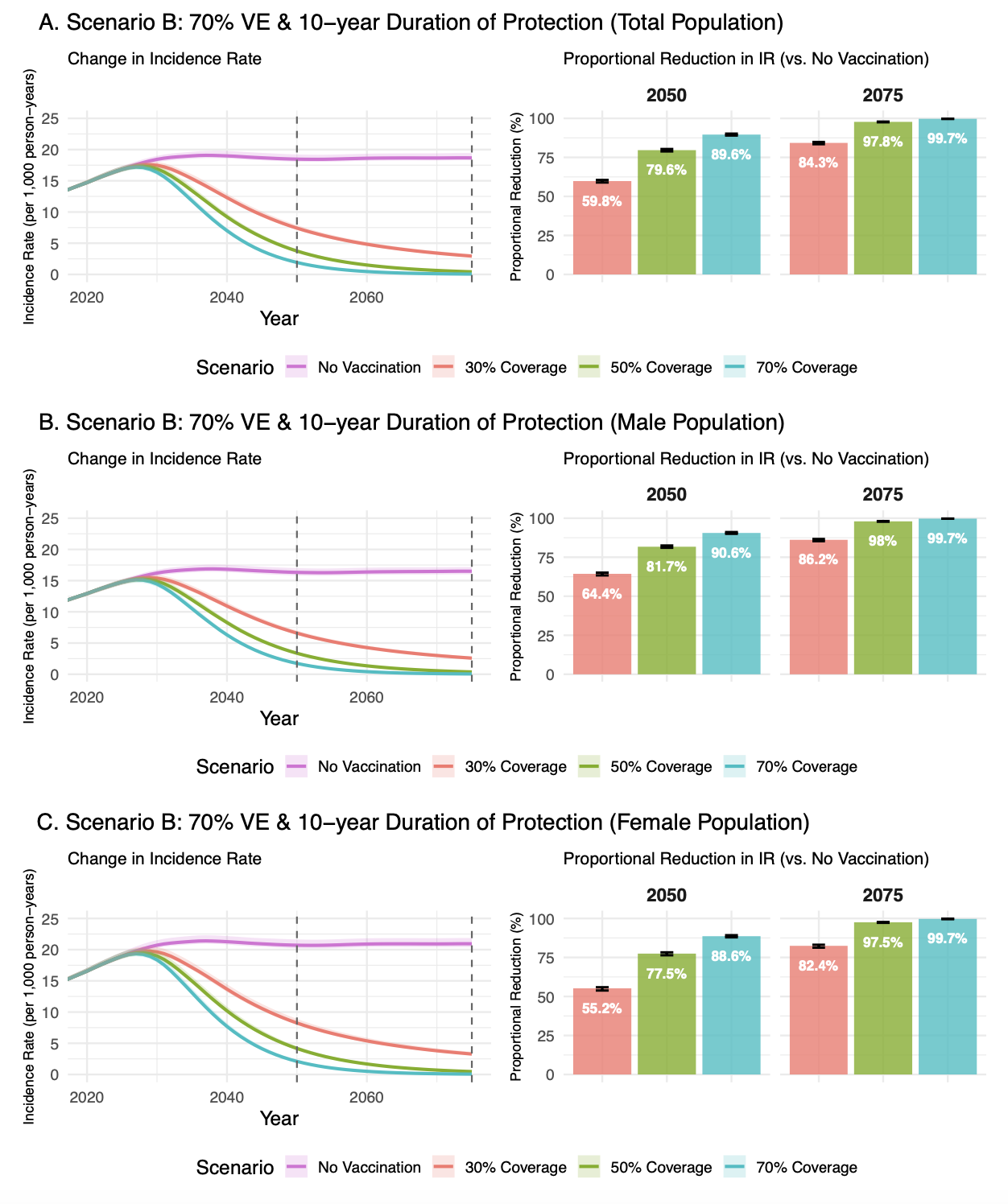


**Figure S20.** The absolute and proportional reduction in CT incidence rates for Scenario B in 2050 and 2075 when compared to the Referent Scenario (no vaccination), assuming 10-year duration of vaccine-conferred immunity and 70% VE (base-case assumption). Results are presented by vaccine coverage attained by 2035 and biological sex. CT incidence rates estimated among individuals aged 15 to 64 years.

**Figure S21.** Infections prevented per 1,000 doses of vaccine given, by vaccine efficacy and duration of protection for Scenario Group A and Scenario Group B (vs. No Vaccination).

**Figure S22.** Infections prevented per 1,000 doses of vaccine given, by vaccine efficacy and duration of protection for Scenario Group C and Scenario Group D (vs. No Vaccination).

**
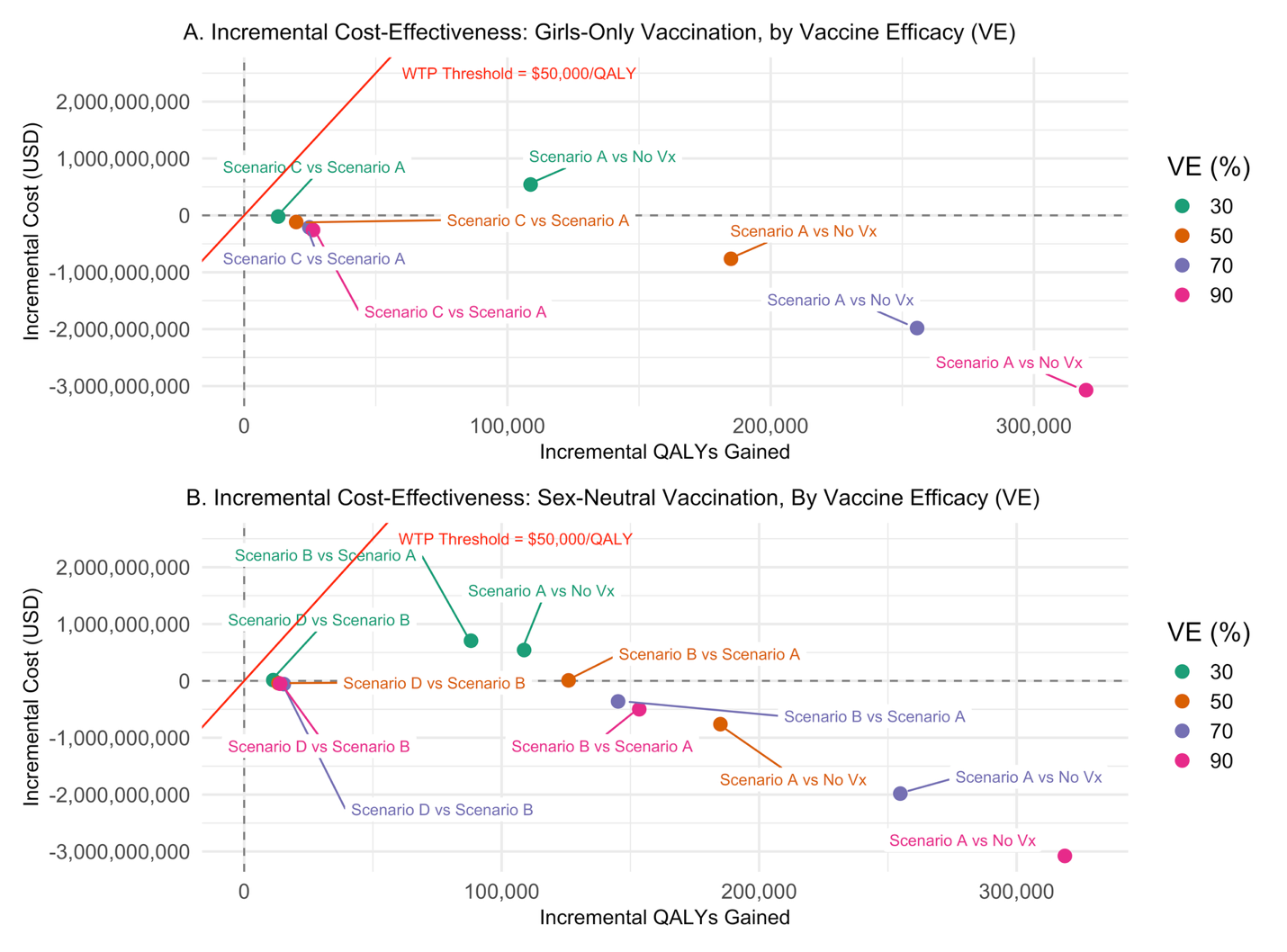
**

**Figure S23.** Cost-effectiveness planes for female-only, sex-neutral, and catch-up CT vaccination strategies under base-case assumptions, varied by vaccine efficacy (2025 - 2050)


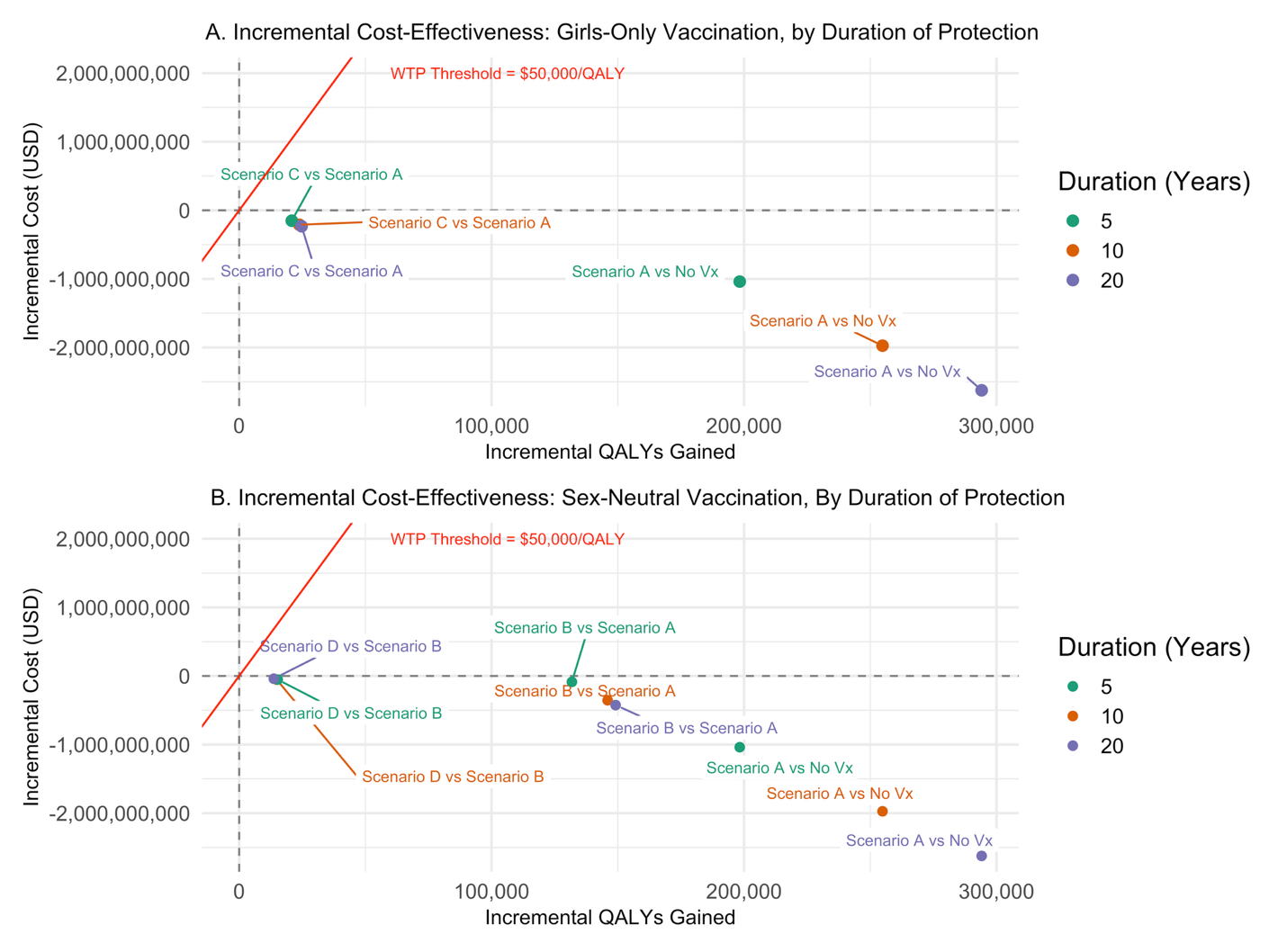


**Figure S24.** Cost-effectiveness planes for female-only, sex-neutral, and catch-up CT vaccination strategies under base-case assumptions, varied by duration of protection (2025 - 2050)


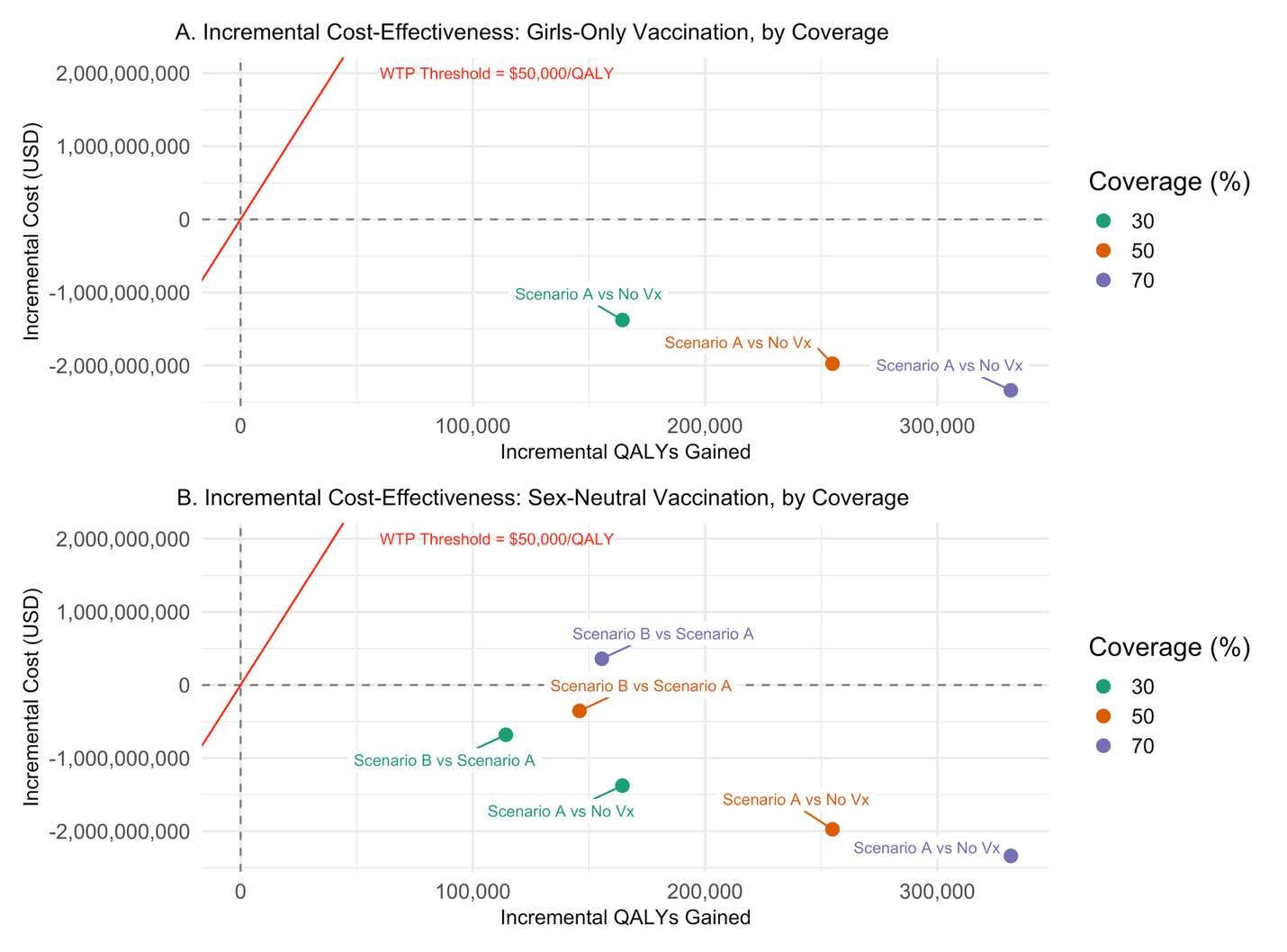


**Figure S25.** Cost-effectiveness planes for female-only, sex-neutral, and catch-up CT vaccination strategies under base-case assumptions, varied by vaccine coverage reached by 2035 (2025 - 2050)

| **Value** | | **Parameter** | **ICERs ($/QALY gained)** | **Results** | |
| --- | --- | --- | --- | --- | --- |
| **Low** | **High** |  |  | **Low** | **High** |
| $25 | $1000 | Price-per-dose |  | -$11,540 | $37,809 |
| $3,230·89 | $9,259·41 | PID txt costs |  | -$3,516 | -$15,747 |
| 0·2072 | 0·6216 | QALYs (PID) |  | -$13,530 | -$5,424 |
| $41·73 | $113·07 | Vaccination visit costs |  | -$9,192 | -$5,581 |
| $189·30 | $363·66 | CT infection txt cost (M) |  | -$6,738 | -$9,135 |
| $193·30 | $345·66 | CT infection txt cost (F) |  | -$6,817 | -$9,088 |
| $113·35 | $265·11 | Screening cost |  | -$8,546 | -$6,776 |
| 1·50% | 5% | Discounting |  | -$8,260 | -$6,973 |
| 0·003 | 0·009 | QALYs (CT Symp. – M) |  | -$8,078 | -$7,436 |
| 0·005 | 0·015 | QALYs (CT Symp. – F) |  | -$7,981 | -$7,520 |
| $567·69 | $2,176·22 | Epididymitis txt costs |  | -$7,590 | -$7,977 |
| 0·005 | 0·015 | QALYs (Epididymitis) |  | -$7,753 | -$7,734 |

**Figure S26.** Tornado plot of one-way sensitivity analyses examining the impact of parameter uncertainty on incremental cost-effectiveness ratios comparing Scenario A vs. No Vaccination, assuming base-case assumptions. Scenario A: Vaccinating 15-year-old females entering the model between 2025-2075.

| **Value** | | **Parameter** | **ICERs ($/QALY gained)** | **Results** | |
| --- | --- | --- | --- | --- | --- |
| **Low** | **High** |  |  | **Low** | **High** |
| $25 | $1000 | Price-per-dose |  | -$12,489 | $35,219 |
| $3,230·89 | $9,259·41 | PID txt costs |  | -$4,562 | -$16,877 |
| 0·2072 | 0·6216 | QALYs (PID) |  | -$15,462 | -$6,169 |
| $41·73 | $113·07 | Vaccination visit costs |  | -$10,219 | -$6,728 |
| $189·30 | $363·66 | CT infection txt cost (M) |  | -$7,837 | -$10,176 |
| 1·50% | 5% | Discounting |  | -$9,756 | -$7,466 |
| $193·30 | $345·66 | CT infection txt cost (F) |  | -$7,897 | -$10,155 |
| $113·35 | $265·11 | Screening cost |  | -$9,308 | -$8,229 |
| 0·003 | 0·009 | QALYs (CT Symp. – M) |  | -$9,189 | -$8,477 |
| 0·005 | 0·015 | QALYs (CT Symp. – F) |  | -$9,081 | -$8,571 |
| $567·69 | $2,176·22 | Epididymitis txt costs |  | -$8,667 | -$9,050 |
| 0·005 | 0·015 | QALYs (Epididymitis) |  | -$8,830 | -$8,808 |

**Figure S27.** Tornado plot of one-way sensitivity analyses examining the impact of parameter uncertainty on incremental cost-effectiveness ratios comparing Scenario C vs. Scenario A, assuming base-case assumptions. Scenario C: Scenario A at 50% coverage + catch-up campaign among unvaccinated 15-24-year-old females in 2035.

| **Value** | | **Parameter** | **ICERs ($/QALY gained)** | **Results** | |
| --- | --- | --- | --- | --- | --- |
| **Low** | **High** |  |  | **Low** | **High** |
| $25 | $1000 | Price-per-dose |  | -$9,286 | $79,876 |
| $3,230·89 | $9,259·41 | PID txt costs |  | $1,744 | -$10,323 |
| $41·73 | $113·07 | Vaccination visit costs |  | -$5,044 | $1,480 |
| 0·2072 | 0·6216 | QALYs (PID) |  | -$4,191 | -$1,708 |
| $189·30 | $363·66 | CT infection txt cost (M) |  | -$1,236 | -$4,075 |
| $193·30 | $345·66 | CT infection txt cost (F) |  | -$1,522 | -$3,741 |
| 1·50% | 5% | Discounting |  | -$3,104 | -$1,417 |
| $113·35 | $265·11 | Screening cost |  | -$2,797 | -$1,981 |
| $567·69 | $2,176·22 | Epididymitis txt costs |  | -$2,242 | -$2,709 |
| 0·003 | 0·009 | QALYs (CT Symp. – M) |  | -$2,552 | -$2,314 |
| 0·005 | 0·015 | QALYs (CT Symp. – F) |  | -$2,499 | -$2,360 |
| 0·005 | 0·015 | QALYs (Epididymitis) |  | -$2,431 | -$2,424 |

**Figure S28.** Tornado plot of one-way sensitivity analyses examining the impact of parameter uncertainty on incremental cost-effectiveness ratios comparing Scenario B vs. Scenario A, assuming base-case assumptions, Scenario B: Vaccinating 15-year-old females and males entering the model between 2025-2075.

| **Value** | | **Parameter** | **ICERs ($/QALY gained)** | **Results (ICER)** | |
| --- | --- | --- | --- | --- | --- |
| **Low** | **High** |  |  | **Low** | **High** |
| $25 | $1000 | Price-per-dose |  | -$9547 | $66,887 |
| $3,230·89 | $9,259·41 | PID txt costs |  | $624 | -$11,792 |
| $41·73 | $113·07 | Vaccination visit costs |  | -$5911 | -$318 |
| 0·2072 | 0·6216 | QALYs (PID) |  | -$6455 | -$2,562 |
| 1·50% | 5% | Discounting |  | -$5068 | -$1,670 |
| $193·30 | $345·66 | CT infection txt cost (F) |  | -$2738 | -$5,017 |
| $189·30 | $363·66 | CT infection txt cost (M) |  | -$2742 | -$4,947 |
| $113·35 | $265·11 | Screening cost |  | -$4173 | -$3,059 |
| $567·69 | $2,176·22 | Epididymitis txt costs |  | -$3523 | -$3,887 |
| 0·003 | 0·009 | QALYs (CT Symp. – M) |  | -$3813 | -$3,533 |
| 0·005 | 0·015 | QALYs (CT Symp. – F) |  | -$3777 | -$3,565 |
| 0·005 | 0·015 | QALYs (Epididymitis) |  | -$3672 | -$3,664 |

**Figure S29.** Tornado plot of one-way sensitivity analyses examining the impact of parameter uncertainty on incremental cost-effectiveness ratios comparing Scenario D vs. Scenario B, assuming base-case assumptions. Scenario D: Scenario B at 50% coverage + catch-up campaign among unvaccinated 15-24-year-old females in 2035.

**Supplementary Tables**

| **Table S1.** Median adjusted annual CT cases in the United States between 2000 and 2022, by demographic groups of interest | | | | | | | | | |
| --- | --- | --- | --- | --- | --- | --- | --- | --- | --- |
| Year | Males  15-24 Years | Males  25-39 Years | Males  40-64 Years | | Females  15-24 Years | | Females  25-39 Years | | Females  40-64 Years |
| 2000 | 380,151 | 187,744 | 83,980 | | 1,559,586 | | 328,589 | | 46,440 |
| 2001 | 420,885 | 206,661 | 82,434 | | 1,584,445 | | 348,353 | | 46,196 |
| 2002 | 452,935 | 228,998 | 86,934 | | 1,510,896 | | 344,204 | | 47,342 |
| 2003 | 457,597 | 227,305 | 74,742 | | 1,423,079 | | 324,969 | | 41,034 |
| 2004 | 473,374 | 234,451 | 71,845 | | 1,324,952 | | 316,437 | | 40,647 |
| 2005 | 493,698 | 245,082 | 70,279 | | 1,239,879 | | 312,141 | | 41,142 |
| 2006 | 507,419 | 252,190 | 64,910 | | 1,155,545 | | 289,895 | | 35,198 |
| 2007 | 544,926 | 265,378 | 65,805 | | 1,134,009 | | 290,872 | | 38,219 |
| 2008 | 598,268 | 279,480 | 65,324 | | 1,148,995 | | 297,165 | | 41,008 |
| 2009 | 613,650 | 276,795 | 63,052 | | 1,113,903 | | 281,009 | | 39,086 |
| 2010 | 645,876 | 285,707 | 60,516 | | 1,106,199 | | 258,167 | | 36,062 |
| 2011 | 694,581 | 307,293 | 66,250 | | 1,187,120 | | 298,810 | | 45,766 |
| 2012 | 685,068 | 321,862 | 71,721 | | 1,137,449 | | 308,665 | | 51,000 |
| 2013 | 649,444 | 332,785 | 73,143 | | 1,057,203 | | 312,692 | | 51,313 |
| 2014 | 654,333 | 360,813 | 79,194 | | 1,030,709 | | 339,562 | | 59,990 |
| 2015 | 688,573 | 397,907 | 87,811 | | 1,050,578 | | 378,997 | | 72,755 |
| 2016 | 724,930 | 433,514 | 93,709 | | 1,062,759 | | 396,700 | | 77,840 |
| 2017 | 771,028 | 468,768 | 103,243 | | 1,135,891 | | 445,125 | | 89,935 |
| 2018 | 785,042 | 489,070 | 108,966 | | 1,158,949 | | 468,244 | | 96,455 |
| 2019 | 811,102 | 516,815 | 113,980 | | 1,139,219 | | 459,155 | | 102,937 |
| 2020 | 674,298 | 424,585 | 86,236 | | 992,607 | | 406,504 | | 83,436 |
| 2021 | 680,036 | 463,212 | 102,504 | | 966,769 | | 432,912 | | 104,610 |
| 2022 | 680,924 | 468,673 | 111,117 | | 935,835 | | 432,687 | | 121,352 |
| Abbreviations: CT, *Chlamydia trachomatis* | | | | | | | | | |
| **Table S2.** Fixed model parameters | | | | | | | | | |
| Parameter Description | | | | Group | | Value | | Source | |
| Initial population size | | | | Females, 15-24 years | | 19,026,294 | | ^12^ | |
|  |  |  |  | Females, 25-39 years | | 30,676,316 | |  |  |
|  |  |  |  | Females, 40-64 years | | 42,859,021 | |  |  |
|  |  |  |  | Males, 15-24 years | | 20,572,554 | |  |  |
|  |  |  |  | Males, 25-39 years | | 32,388,974 | |  |  |
|  |  |  |  | Males, 40-64 years | | 41,988,110 | |  |  |
| “Birth” rate (*η)* | | | | Females/Males | | Year-dependent | | ^12^ | |
| Aging rate (*χ _j,k_*) | | | | By age group | | Year-dependent | | ^12^ | |
| Migration rate (*ξ*) | | | | All groups | | Year-dependent | | ^12^ | |
| Background mortality rate | | | | All groups | | Year-dependent | | ^12^ | |
| Incubation period (*ϕ*) | | | | All groups | | 14 days | | ^2,5^ | |
| Average duration of infection-conferred immunity (*ψ*) | | | | All groups | | 1 year | | ^2,32^ | |
| Probability of treatment, symptomatic (*p_txt)* | | | | All groups | | 0·89 | | ^2,33^ | |
| Treatment efficacy (*tx_eff)* | | | | All groups | | 0·95 | | ^2,34^ | |
| Sensitivity of CT diagnostic test (*ε*) | | | | All groups | | 0·96 | | ^35–37^ | |
| Post-screening treatment probability (*υ*) | | | | All groups | | 0·80 | | ^2,38^ | |
| Average duration of sequelae (epididymitis for males; PID and complications for females; *ϖ _j_*) | | | | Females | | 60 days | | ^2,39,40^ | |
|  |  |  |  | Males | | 21 days | |  |  |

| **Table S3. Uncertain parameters for model calibration** | | | |
| --- | --- | --- | --- |
| **Parameter Description** | **Group** | **Range** | **Source** |
| Probability of symptomatic infection  (*ζ _j_*) | Females | 0·1 – 0·3 | ^1,2,11,43,44^ |
|  | Males | 0·4 – 0·6 | ^1,2,11,43,44^ |
| Probability of sequelae, given untreated infection (*κ _j_*) | Females | 0·10 – 0·14 | ^2,45,46^ |
|  | Males | 0·01 – 0·03 | ^2,47,48^ |
| Duration of treated symptomatic infection (*dur_tx _j_*) | Females | 35 – 49 days | ^1,5,11^ |
|  | Males | 28 – 42 days | ^1,5,11^ |
| Duration of untreated infection (to natural resolution; *ι*) | All groups | 1·25 – 2·0 years | ^2,5,49,50^ |
| Probability of annual screening for CT (*r _j,k_*) | Females, 15-24 years | 0·35 – 0·48 | ^2,14,21,25,26,51^ |
|  | Females, 25-39 years | 0·35 – 0·41 |  |
|  | Females, 40-64 years | 0·18 – 0·25 |  |
|  | Males, 15-24 years | 0·01 – 0·02 | ^2,17,29,30^ |
|  | Males, 25-39 years | 0·04 – 0·06 |  |
|  | Males, 40-64 years | 0·03 – 0·05 |  |
| Sexual partner change (number of sexual partners in the past 12 months; *c _j,k_*) | Females, 15-24 years | 2·4 – 2·9 | ^2,48,52,53, Model assumption^ |
|  | Females, 25-39 years | 1·2 – 1·6 |  |
|  | Females, 40-64 years | 0·6 – 1·1 |  |
|  | Males, 15-24 years | 0·8 – 1·1 |  |
|  | Males, 25-39 years | 0·8 – 1·2 |  |
|  | Males, 40-64 years | 0·5 – 0·8 |  |
| Per partner transmission probability () | Female-to-Male | 0·8 – 0·99 | ^2,54, Model assumption^ |
|  | Male-to-Female | 0·8 – 0·99 |  |
| Assortative mixing by age group (*ρ*) | All groups | 0·85 – 0·95 | ^1,2,55, Model assumption^ |

| **Table S4.** Referent and Intervention Scenarios under Base-case Assumptions ^1^ | | | | | |
| --- | --- | --- | --- | --- | --- |
| **Scenario** | **Description** | **Target Population** | **Coverage** | **Start Year** | **Catch-up Campaign** |
| Referent | Calibrated model without vaccination; reflects U.S. standard of care (screen-and-treat for asymptomatic, test-and-treat for symptomatic) | N/A | N/A | N/A | No |
| A | Female-only routine vaccination | 15-year-old females | Linear scale-up to 50% by 2035 | 2025 | No |
| B | Sex-neutral routine vaccination | 15-year-old females and males | Linear scale-up to 50% by 2035 | 2025 | No |
| C | Female-only routine + catch-up | Scenario A + unvaccinated females aged 15–24 | 50% routine + 10% catch-up (2035) | 2025 (routine), 2035  (catch-up) | Yes (females 15–24 years) |
| D | Sex-neutral routine + catch-up | Scenario B + unvaccinated females aged 15–24 | 50% routine + 10% catch-up (2035) | 2025 (routine), 2035  (catch-up) | Yes (females 15–24 years) |

^1^ Base-case assumes 10 years of vaccine-conferred immunity, 70% vaccine efficacy, and 50% routine coverage attained by 2035, with 10% coverage for a catch-up campaign in 2035.

| **Table S5.** Vaccination parameters for scenario analysis | | |
| --- | --- | --- |
| **Parameter Description** | **Group** | **Range** |
| Vaccination coverage in 2035 (increasing linearly beginning in 2025), among individuals entering model @ 15 years (*Vx _j_*) | Females | 30%, 50%, 70% |
|  | Males | 0%, 30%, 50%, 70% |
| Vaccination coverage via catch-up campaigns in 2035, among 15-24-year-olds only (*β _j,k_*) | Females | 5%, 10%, 15% |
| Duration of vaccine-conferred immunity (*θ _k_*) | All groups | 0.5, 1, 5, 10, 20 years |
| Vaccine efficacy, defined as the proportional reduction in susceptibility to infection, comparing vaccinated to unvaccinated individuals (*VE_S_*) | All groups | 10%, 20%, 30%, 40%, 50%, 60%, 70%, 80%, 90% |

| **Table S6.** Epidemiologic Outcomes of Interest | |
| --- | --- |
| **Outcome** | **Definition** |
| 1. Cumulative CT infections prevented by 2075 | Absolute difference in total CT infections incurred between 2025 – 2075, comparing the vaccination scenario of interest to the referent scenario (no vaccination). |
| 1. Proportional reduction in CT infections prevented by 2075 | Proportional difference in total CT infections incurred between 2025 – 2075, comparing the vaccination scenario of interest to the referent scenario (no vaccination) and reported as a percentage. |
| 1. Reduction in annual CT incidence rates by 2050 and 2075 | CT incidence rates estimated annually between 2025 – 2075, measured as the number of incident infections per 1,000 person-years. Reduction in CT incidence rates compared the vaccination scenario of interest to the referent scenario (no vaccination). |
| 1. Infections prevented per 1,000 vaccination doses by 2075 | Quotient of the cumulative CT infections prevented and the total number of vaccine doses administered between 2025 – 2075 (used as a proxy for evaluating the efficiency of each vaccine implementation strategy). |

Abbreviations: CT, *Chlamydia trachomatis*

| **Table S7.** Outline of Vaccination Parameters & QALY- and Cost-Associated Parameters ^A^ | | | |
| --- | --- | --- | --- |
| **A. Vaccination Parameters** | | | |
| Parameter Description | Group | Value/Range | Sources |
| Vaccination coverage in 2035 (increasing linearly beginning in 2025), among individuals entering model @ 15 years (*Vx _j_*) | Females | 30%, 50%, 70% | Assumption |
|  | Males | 0%, 30%, 50%, 70% | Assumption |
| Vaccination coverage via catch-up campaigns in 2035, among 15-24-year-olds only (*β _j,k_*) | Females | 5%, 10%, 15% | Assumption |
| Duration of vaccine-conferred immunity (*θ _k_*) | All groups | 5, 10, or 20 years | Assumption |
| Vaccine efficacy, defined as the proportional reduction in susceptibility to infection, comparing vaccinated to unvaccinated individuals (*VE_S_*) | All groups | 30%, 50%, 70%, 90% | Assumption |
| **B. QALY- and Cost-associated Parameters** | | | |
| Parameter Description | Group | Value/Range | Sources |
| Discount rate per year (*d)* | All groups | 3% | Assumption |
| Probability of symptomatic infection  (*ζ _j_*) | Females | 0·1 – 0·3 | ^1,2,11,43,44^ |
|  | Males | 0·4 – 0·6 | ^1,2,11,43,44^ |
| Incubation period (*ϕ*) | All groups | 14 days | ^2,5^ |
| Duration of untreated infection (to natural resolution; *ι*) | All groups | 1·25 – 2·0 years | ^2,5,49,50^ |
| Probability of sequelae, given untreated infection (*κ _j_*) | Females | 0·10 – 0·14 | ^2,45,46^ |
|  | Males | 0·01 – 0·03 | ^2,47,48^ |
| Probability of annual screening for CT (*r _j,k_*) | Females,  15-24 years | 0·35 – 0·48 | ^2,14,21,25,26,51^ |
|  | Females,  25-39 years | 0·35 – 0·41 |  |
|  | Females,  40-64 years | 0·18 – 0·25 |  |
|  | Males,  15-24 years | 0·01 – 0·02 | ^2,17,29,30^ |
|  | Males,  25-39 years | 0·04 – 0·06 |  |
|  | Males,  40-64 years | 0·03 – 0·05 |  |
| Sensitivity of CT diagnostic test (*ε*) | All groups | 0·96 | ^35–37^ |
| Specificity of CT diagnostic test (*spec*) | All groups | 0·99 | ^35–37^ |
| Post-screening treatment probability (*υ*) | All groups | 0·80 | ^2,38^ |
| Probability of treatment, symptomatic (*p_txt)* | All groups | 0·89 | ^2,33^ |
| Duration of treated symptomatic infection (*dur_tx _j_*) | Females | 35 – 49 days | ^1,5,11^ |
|  | Males | 28 – 42 days | ^1,5,11^ |
| Sequelae resolution (*ϖ _j_*) | Females | 60 days | ^2,39^ |
|  | Males | 21 days | ^2^ |
| “Birth” rate (*η)* | Females/Males | Year-dependent | ^12^ |
| ^A^ Parameter values necessary for cost-effectiveness analyses are provided above, with additional detail on remaining model parameters available in Appendix A.  Abbreviations: CT, *Chlamydia trachomatis*. | | | |

| **Table S8.** Outline of Cost Estimates | | | | |
| --- | --- | --- | --- | --- |
| **A. Costs Applied to Both Sexes** | | | | |
| Category | Direct Costs ^A^  (USD 2025) | Indirect Costs ^B^  (USD 2025) | Total Costs ^C^  (2025 USD) | Sources |
| Vaccination  (Implementation) ^D^ | $35·17  ($26·38, $43·96) | $35·17  ($15·35, $69·11) | $70·34  ($41·73, $113·07) | ^2,60–65^ |
| Vaccination  (Price Per Dose) | $ per dose  ($25 - $1000) | N/A | $ per dose  ($25 - $1000) | ^65^ |
| Screening without Treatment | $147·00  ($98·00, $196·00) | $35·17  ($15·35, $69·11) | $182·17  ($113·35, $265·11) | ^2,58,60,66–68^ |
| **B. Female-Specific Costs** | | | | |
| Category | Direct Costs ^A^  (USD 2025) | Indirect Costs ^B^  (USD 2025) | Total Costs ^C^  (2025 USD) | Sources |
| Treatment of Acute Infection | $187·00  ($164·00, $210·00) | $68·48  ($29·30, $135·66) | $255·48  ($193·30, $345·66) | ^60,66,69^ |
| Treatment of PID ^E^ | $3,049·26  ($2,377·03, $4,568·92) | $2,265·49  ($853·86, $4,690·49) | $5,314·75  ($3230·89, $9259·41) | ^60,69^ |
| **C. Male-Specific Costs** | | | | |
| Category | Direct Costs ^A^  (USD 2025) | Indirect Costs ^B^  (USD 2025) | Total Costs ^C^  (2025 USD) | Sources |
| Treatment of Acute Infection | $194·00  ($160·00, $228·00) | $68·48  ($29·30, $135·66) | $262·48  ($189·30, $363·66) | ^60,66,69^ |
| Treatment of Epididymitis | $465·96  ($288·28, $643·65) | $740·22  ($279·41, $1,532·57) | $1,206·18  ($567·69, $2,176·22) | ^60,69^ |
| ^A^ Direct costs include all medical costs (supplies, diagnostics, provider salaries, etc.) for one unit of the corresponding category.  ^B^ Indirect costs represent productivity losses for one unit of the corresponding category, capturing the value of time lost due to travel, waiting, and receipt of CT-related services.  ^C^ Total costs include the total direct and indirect costs for one unit of the corresponding category.  ^D^ Implementation costs represent per-dose vaccine administration costs (e.g., provider time, clinical visit costs, and supplies) based on published estimates of vaccine administration in U.S. clinical settings. These costs reflect recurrent delivery expenses applied per dose and do not include one-time vaccine introduction costs such as training, program start-up activities, or social mobilization.  ^E^ Costs for PID were estimated by combining the probability and discounted cost of each PID-related outcome (outpatient and inpatient treatment, ectopic pregnancy, tubal factor infertility, chronic pelvic pain), using a 3% annual discount rate and assuming the time from infection to PID onset was under one year.  Costs are presented as main unit cost (lower bound, upper bound). Abbreviations: PID, pelvic inflammatory disease; CT, *Chlamydia trachomatis*. | | | | |

| **Table S9.** Outline of Health Utilities (QALYs) ^A^ | | | |
| --- | --- | --- | --- |
| Category | Females | Males | Sources |
| Acute Symptomatic  CT Infection | 0·010  (0·005, 0·015) | 0·006  (0·003, 0·009) | ^2,48,57^ |
| Acute Asymptomatic  CT Infection ^B^ | 0 | 0 | ^2,58,59^ |
| Epididymitis | -- | 0.010  (0·005, 0·015) | ^2,48,57^ |
| PID & Complications ^C^ | 0·4144  (0·2072, 0·6216) | -- | ^2,48,59^ |
| ^A^ Health utilities represent average QALYs lost per incident event.  ^B^ Asymptomatic CT infection assumed to have no impact on QALYs  ^C^ QALYs lost for PID accounts for the ‘average’ case of PID, based on probability of further development of chronic pelvic pain, ectopic pregnancy, infertility, or no other complications  Abbreviations: QALYs, quality-adjusted life-years; CT, *Chlamydia trachomatis;* PID, pelvic inflammatory disease | | | |

| **Table S10.** Referent and Intervention Scenarios | | | | | |
| --- | --- | --- | --- | --- | --- |
| **Scenario** | **Description** | **Target Population** | **Coverage** | **Start Year** | **Catch-up Campaign** |
| Referent | Calibrated model without vaccination; reflects U.S. standard of care (screen-and-treat for asymptomatic, test-and-treat for symptomatic) | N/A | N/A | N/A | No |
| A | Female-only routine vaccination | 15-year-old females | Linear scale-up to 50% by 2035 | 2025 | No |
| B | Sex-neutral routine vaccination | 15-year-old females and males | Linear scale-up to 50% by 2035 | 2025 | No |
| C | Female-only routine + catch-up | Scenario A + unvaccinated females aged 15–24 | 50% routine + 10% catch-up (2035) | 2025 (routine), 2035  (catch-up) | Yes (females 15–24 years) |
| D | Sex-neutral routine + catch-up | Scenario B + unvaccinated females aged 15–24 | 50% routine + 10% catch-up (2035) | 2025 (routine), 2035  (catch-up) | Yes (females 15–24 years) |

| **Table S11.** Outline of Incremental Cost-Effectiveness Ratios (ICERs) | | | | |
| --- | --- | --- | --- | --- |
| ICER |  | Comparison Groups |  | ICER Equation |
| 1 |  | Scenario A vs  Referent Scenario ^A^ |  | $ICER= \frac{{Cost}_{Scenario A}-{Cost}_{Referent}}{{QALYs}_{Scenario A}- {QALYs}_{Referent}}$ |
| 2 |  | Scenario B vs Scenario A |  | $ICER= \frac{{Cost}_{Scenario B}-{Cost}_{Scenario A}}{{QALYs}_{Scenario B}- {QALYs}_{Scenario A}}$ |
| 3 |  | Scenario C vs Scenario A |  | $ICER= \frac{{Cost}_{Scenario C}-{Cost}_{Scenario A}}{{QALYs}_{Scenario C}- {QALYs}_{Scenario A}}$ |
| 4 |  | Scenario D vs Scenario B |  | $ICER= \frac{{Cost}_{Scenario D}-{Cost}_{Scenario B}}{{QALYs}_{Scenario D}- {QALYs}_{Scenario B}}$ |
| ^A^ Referent Scenario: standard of care in the United States (screen-and-treat for asymptomatic infections and test-and-treat for symptomatic infections) without vaccination. | | | | |

| **Table S12.** Outline of Scenario Analysis | | | | | | |
| --- | --- | --- | --- | --- | --- | --- |
| Scenario # | Scenario Group | Coverage by 2035 ^A^ | Duration of Protection | VE | Catch-up Coverage ^B^ | Difference to Base-case |
| 1 | A | 50% | 10 Years | 70% | -- | -- * |
| 2 | A | 50% | 10 Years | 30% | -- | VE |
| 3 | A | 50% | 10 Years | 50% | -- | VE |
| 4 | A | 50% | 10 Years | 90% | -- | VE |
| 5 | A | 50% | 5 Years | 70% | -- | Duration of Protection |
| 6 | A | 50% | 20 Years | 70% | -- | Duration of Protection |
| 7 | A | 30% | 10 Years | 70% | -- | Coverage |
| 8 | A | 70% | 10 Years | 70% | -- | Coverage |
| 9 | B | 50% | 10 Years | 70% | -- | -- * |
| 10 | B | 50% | 10 Years | 30% | -- | VE |
| 11 | B | 50% | 10 Years | 50% | -- | VE |
| 12 | B | 50% | 10 Years | 90% | -- | VE |
| 13 | B | 50% | 5 Years | 70% | -- | Duration of Protection |
| 14 | B | 50% | 20 Years | 70% | -- | Duration of Protection |
| 15 | B | 30% | 10 Years | 70% | -- | Coverage |
| 16 | B | 70% | 10 Years | 70% | -- | Coverage |
| 17 | C | 50% | 10 Years | 70% | 10% | -- * |
| 18 | C | 50% | 10 Years | 30% | 10% | VE |
| 19 | C | 50% | 10 Years | 50% | 10% | VE |
| 20 | C | 50% | 10 Years | 90% | 10% | VE |
| 21 | C | 50% | 5 Years | 70% | 10% | Duration of Protection |
| 22 | C | 50% | 20 Years | 70% | 10% | Duration of Protection |
| 23 | C | 50% | 10 Years | 70% | 5% | Coverage (Catch-up) |
| 24 | C | 50% | 10 Years | 70% | 15% | Coverage (Catch-up) |
| 25 | D | 50% | 10 Years | 70% | 10% | -- * |
| 26 | D | 50% | 10 Years | 30% | 10% | VE |
| 27 | D | 50% | 10 Years | 50% | 10% | VE |
| 28 | D | 50% | 10 Years | 90% | 10% | VE |
| 29 | D | 50% | 5 Years | 70% | 10% | Duration of Protection |
| 30 | D | 50% | 20 Years | 70% | 10% | Duration of Protection |
| 31 | D | 50% | 10 Years | 70% | 5% | Coverage (Catch-up) |
| 32 | D | 50% | 10 Years | 70% | 15% | Coverage (Catch-up) |
| ^A^ Vaccination coverage among 15-year-olds (females and/or males, dependent on Scenario Group) entering the simulated population, beginning in 2025. Value represents coverage reached linearly by 2035.  ^B^ Vaccination coverage among unvaccinated 15-24-year-old females for catch-up campaign occurring in 2035, only.  * Represents base-case scenario within Scenario Group  Abbreviation: VE, vaccine efficacy | | | | | | |

| **Table S13**. Absolute and relative (%) reduction in CT infections between 2025-2075, comparing Scenario A^1^ to No Vaccination by vaccine coverage, efficacy, and duration of protection. | | | | | | | | | |
| --- | --- | --- | --- | --- | --- | --- | --- | --- | --- |
| **Duration of Protection** | **VE** | **30% Coverage** | |  | **50% Coverage** | |  | **70% Coverage** | |
|  |  | Absolute Reduction^2^ | Relative Reduction^3^ |  | Absolute Reduction | Relative Reduction |  | Absolute Reduction | Relative Reduction |
| 0.5 Years | 10% | 971,733 | 0·3 |  | 1,618,900 | 0·6 |  | 2,263,009 | 0·8 |
|  | 20% | 2,138,003 | 0·8 |  | 3,557,957 | 1·3 |  | 4,971,480 | 1·8 |
|  | 30% | 3,305,872 | 1·2 |  | 5,497,563 | 2·0 |  | 7,677,590 | 2·8 |
|  | 40% | 4,479,832 | 1·6 |  | 7,444,171 | 2·7 |  | 10,388,818 | 3·7 |
|  | 50% | 5,659,396 | 2·0 |  | 9,396,576 | 3·4 |  | 13,103,960 | 4·7 |
|  | 60% | 6,843,523 | 2·5 |  | 11,353,840 | 4·1 |  | 15,820,635 | 5·7 |
|  | 70% | 8,032,443 | 2·9 |  | 13,315,772 | 4·8 |  | 18,540,964 | 6·7 |
|  | 80% | 9,225,987 | 3·3 |  | 15,281,749 | 5·5 |  | 21,262,681 | 7·6 |
|  | 90% | 10,424,471 | 3·7 |  | 17,252,717 | 6·2 |  | 23,976,204 | 8·6 |
| 1 Year | 10% | 1,805,529 | 0·6 |  | 3,005,324 | 1·1 |  | 4,197,158 | 1·5 |
|  | 20% | 3,985,157 | 1·4 |  | 6,622,505 | 2·4 |  | 9,242,282 | 3·3 |
|  | 30% | 6,176,991 | 2·2 |  | 10,249,769 | 3·7 |  | 14,282,902 | 5·1 |
|  | 40% | 8,379,850 | 3·0 |  | 13,886,583 | 5·0 |  | 19,324,025 | 6·9 |
|  | 50% | 10,596,058 | 3·8 |  | 17,531,973 | 6·3 |  | 24,363,009 | 8·7 |
|  | 60% | 12,821,979 | 4·6 |  | 21,185,863 | 7·6 |  | 29,376,915 | 10·6 |
|  | 70% | 15,062,835 | 5·4 |  | 24,839,163 | 8·9 |  | 34,364,535 | 12·3 |
|  | 80% | 17,313,389 | 6·2 |  | 28,483,631 | 10·2 |  | 39,339,163 | 14·1 |
|  | 90% | 19,572,325 | 7·0 |  | 32,131,588 | 11·5 |  | 44,281,527 | 15·9 |
| 5 Years | 10% | 6,308,878 | 2·3 |  | 10,452,201 | 3·8 |  | 14,537,831 | 5·2 |
|  | 20% | 13,658,059 | 4·9 |  | 22,513,663 | 8·1 |  | 31,153,821 | 11·2 |
|  | 30% | 21,011,347 | 7·5 |  | 34,439,190 | 12·4 |  | 47,305,999 | 17·0 |
|  | 40% | 28,372,156 | 10·2 |  | 46,148,580 | 16·6 |  | 62,749,927 | 22·5 |
|  | 50% | 35,695,348 | 12·8 |  | 57,507,191 | 20·7 |  | 77,281,207 | 27·8 |
|  | 60% | 42,965,976 | 15·4 |  | 68,497,806 | 24·6 |  | 90,550,674 | 32·5 |
|  | 70% | 50,143,618 | 18·0 |  | 78,843,032 | 28·3 |  | 102,591,441 | 36·9 |
|  | 80% | 57,226,022 | 20·5 |  | 88,597,608 | 31·8 |  | 113,186,108 | 40·7 |
|  | 90% | 64,110,079 | 23·0 |  | 97,686,136 | 35·1 |  | 122,399,714 | 44·0 |
| 10 Years | 10% | 9,231,207 | 3·3 |  | 15,252,598 | 5·5 |  | 21,168,745 | 7·6 |
|  | 20% | 19,690,317 | 7·1 |  | 32,255,098 | 11·6 |  | 44,306,628 | 15·9 |
|  | 30% | 30,067,676 | 10·8 |  | 48,684,532 | 17·5 |  | 65,876,794 | 23·7 |
|  | 40% | 40,292,302 | 14·5 |  | 64,322,993 | 23·1 |  | 85,405,873 | 30·7 |
|  | 50% | 50,352,929 | 18·1 |  | 78,933,473 | 28·3 |  | 102,284,372 | 36·8 |
|  | 60% | 60,100,963 | 21·6 |  | 92,216,103 | 33·1 |  | 116,387,709 | 41·9 |
|  | 70% | 69,515,281 | 25·0 |  | 104,104,432 | 37·4 |  | 127,740,899 | 46·0 |
|  | 80% | 78,457,331 | 28·2 |  | 114,437,796 | 41·2 |  | 136,976,713 | 49·3 |
|  | 90% | 86,949,690 | 31·2 |  | 123,295,768 | 44·4 |  | 144,299,940 | 51·9 |
| 20 Years | 10% | 11,948,562 | 4·3 |  | 19,684,815 | 7·1 |  | 27,222,529 | 9·8 |
|  | 20% | 25,150,618 | 9·0 |  | 40,893,190 | 14·7 |  | 55,621,532 | 20·0 |
|  | 30% | 38,108,044 | 13·7 |  | 60,831,627 | 21·9 |  | 80,841,382 | 29·1 |
|  | 40% | 50,671,977 | 18·2 |  | 79,037,535 | 28·4 |  | 102,131,847 | 36·7 |
|  | 50% | 62,724,183 | 22·5 |  | 95,288,463 | 34·2 |  | 118,855,840 | 42·7 |
|  | 60% | 74,126,442 | 26·7 |  | 109,058,318 | 39·2 |  | 131,583,541 | 47·3 |
|  | 70% | 84,871,956 | 30·5 |  | 120,536,021 | 43·4 |  | 141,313,388 | 50·8 |
|  | 80% | 94,713,900 | 34·0 |  | 129,919,938 | 46·8 |  | 148,638,860 | 53·5 |
|  | 90% | 103,687,242 | 37·3 |  | 137,703,227 | 49·5 |  | 154,292,082 | 55·5 |

^1^ Scenario A: Vaccinating only 15-year-old females entering the model between 2025-2075.

^2^ Refers to difference in total CT infections comparing Scenario A to the referent scenario (no vaccination) from 2025-2075.

^3^ Refers to percent reduction in total CT infections comparing Scenario A to the referent scenario (no vaccination) from 2025-2075.

Abbreviations: CT, *Chlamydia trachomatis*; VE, vaccine efficacy.

| **Table S14**. Absolute and relative reduction in CT infections between 2025-2075, comparing Scenario B^1^ to No Vaccination by vaccine coverage, efficacy, and duration of protection. | | | | | | | | | |
| --- | --- | --- | --- | --- | --- | --- | --- | --- | --- |
| **Duration of Protection** | **VE** | **30% Coverage** | |  | **50% Coverage** | |  | **70% Coverage** | |
|  |  | Absolute Reduction^2^ | Relative Reduction^3^ |  | Absolute Reduction | Relative Reduction |  | Absolute Reduction | Relative Reduction |
| 0.5 Years | 10% | 1,947,395 | 0·7 |  | 3,242,243 | 1·2 |  | 4,529,475 | 1·6 |
|  | 20% | 4,224,743 | 1·5 |  | 7,019,001 | 2·5 |  | 9,793,234 | 3·5 |
|  | 30% | 6,500,300 | 2·3 |  | 10,781,429 | 3·9 |  | 15,013,875 | 5·4 |
|  | 40% | 8,775,877 | 3·1 |  | 14,527,360 | 5·2 |  | 20,191,510 | 7·2 |
|  | 50% | 11,050,682 | 4·0 |  | 18,255,590 | 6·6 |  | 25,325,789 | 9·1 |
|  | 60% | 13,322,646 | 4·8 |  | 21,967,352 | 7·9 |  | 30,409,607 | 10·9 |
|  | 70% | 15,591,952 | 5·6 |  | 25,659,655 | 9·2 |  | 35,440,381 | 12·7 |
|  | 80% | 17,858,333 | 6·4 |  | 29,326,727 | 10·5 |  | 40,423,790 | 14·5 |
|  | 90% | 20,125,677 | 7·2 |  | 32,970,627 | 11·8 |  | 45,300,306 | 16·3 |
| 1 Year | 10% | 3,620,123 | 1·3 |  | 6,017,375 | 2·2 |  | 8,395,106 | 3·0 |
|  | 20% | 7,880,784 | 2·8 |  | 13,057,758 | 4·7 |  | 18,156,412 | 6·5 |
|  | 30% | 12,136,292 | 4·4 |  | 20,025,457 | 7·2 |  | 27,736,121 | 10·0 |
|  | 40% | 16,372,735 | 5·9 |  | 26,910,566 | 9·7 |  | 37,123,897 | 13·3 |
|  | 50% | 20,595,563 | 7·4 |  | 33,708,676 | 12·1 |  | 46,294,282 | 16·6 |
|  | 60% | 24,799,097 | 8·9 |  | 40,422,588 | 14·5 |  | 55,206,595 | 19·8 |
|  | 70% | 28,974,220 | 10·4 |  | 46,996,610 | 16·9 |  | 63,842,560 | 22·9 |
|  | 80% | 33,139,659 | 11·9 |  | 53,465,701 | 19·2 |  | 72,096,564 | 25·9 |
|  | 90% | 37,264,705 | 13·4 |  | 59,793,299 | 21·4 |  | 79,942,212 | 28·7 |
| 5 Years | 10% | 12,560,323 | 4·5 |  | 20,687,703 | 7·4 |  | 28,599,107 | 10·3 |
|  | 20% | 26,714,866 | 9·6 |  | 43,375,544 | 15·6 |  | 58,856,764 | 21·1 |
|  | 30% | 40,470,845 | 14·5 |  | 64,356,053 | 23·1 |  | 85,227,316 | 30·6 |
|  | 40% | 53,666,062 | 19·3 |  | 83,292,385 | 29·9 |  | 106,749,230 | 38·4 |
|  | 50% | 66,227,376 | 23·8 |  | 99,699,777 | 35·8 |  | 123,488,397 | 44·4 |
|  | 60% | 77,980,043 | 28·0 |  | 113,565,158 | 40·8 |  | 136,137,167 | 49·0 |
|  | 70% | 88,787,585 | 31·9 |  | 124,930,685 | 44·9 |  | 145,550,591 | 52·4 |
|  | 80% | 98,727,601 | 35·5 |  | 134,258,437 | 48·3 |  | 152,649,002 | 54·9 |
|  | 90% | 107,699,645 | 38·7 |  | 141,769,166 | 51·0 |  | 158,106,721 | 56·9 |
| 10 Years | 10% | 18,278,014 | 6·6 |  | 29,924,555 | 10·7 |  | 41,011,396 | 14·7 |
|  | 20% | 38,064,137 | 13·7 |  | 60,580,168 | 21·8 |  | 80,286,972 | 28·9 |
|  | 30% | 56,670,200 | 20·4 |  | 86,925,767 | 31·3 |  | 110,167,154 | 39·6 |
|  | 40% | 73,749,547 | 26·5 |  | 108,205,467 | 38·9 |  | 130,472,434 | 47·0 |
|  | 50% | 89,064,430 | 32·0 |  | 124,291,034 | 44·7 |  | 144,176,307 | 51·8 |
|  | 60% | 102,457,719 | 36·8 |  | 136,394,100 | 49·1 |  | 153,346,587 | 55·2 |
|  | 70% | 113,920,458 | 41·0 |  | 145,407,355 | 52·3 |  | 159,787,984 | 57·5 |
|  | 80% | 123,489,031 | 44·4 |  | 152,168,291 | 54·7 |  | 164,538,431 | 59·2 |
|  | 90% | 131,520,263 | 47·3 |  | 157,383,524 | 56·6 |  | 168,211,152 | 60·6 |
| 20 Years | 10% | 23,528,367 | 8·4 |  | 38,162,802 | 13·7 |  | 51,807,501 | 18·6 |
|  | 20% | 47,982,765 | 17·2 |  | 74,630,038 | 26·8 |  | 96,403,946 | 34·6 |
|  | 30% | 70,023,429 | 25·2 |  | 103,269,392 | 37·1 |  | 125,420,551 | 45·1 |
|  | 40% | 89,280,634 | 32·1 |  | 123,716,601 | 44·5 |  | 142,860,512 | 51·4 |
|  | 50% | 105,466,508 | 37·9 |  | 137,820,818 | 49·6 |  | 153,604,518 | 55·2 |
|  | 60% | 118,510,909 | 42·6 |  | 147,686,470 | 53·1 |  | 160,633,793 | 57·8 |
|  | 70% | 128,876,958 | 46·4 |  | 154,666,652 | 55·6 |  | 165,645,391 | 59·6 |
|  | 80% | 137,264,608 | 49·4 |  | 159,831,312 | 57·5 |  | 169,429,158 | 61·0 |
|  | 90% | 143,958,455 | 51·8 |  | 163,834,883 | 59·0 |  | 172,409,745 | 62·1 |

^1^ Scenario B: Vaccinating 15-year-old females and males entering the model between 2025-2075.

^2^ Refers to difference in total CT infections comparing Scenario B to the referent scenario (no vaccination) from 2025-2075.

^3^ Refers to percent reduction in total CT infections comparing Scenario B to the referent scenario (no vaccination) from 2025-2075.

Abbreviations: CT, *Chlamydia trachomatis*; VE, vaccine efficacy.

| **Table S15**. Absolute and relative reduction in CT infections between 2025-2075, comparing Scenario C^1^ at 50% routine coverage to No Vaccination by catch-up coverage, efficacy, and duration of protection. | | | | | | | | | |
| --- | --- | --- | --- | --- | --- | --- | --- | --- | --- |
| **Duration of Protection** | **VE** | **5% Catch-up Coverage** | |  | **10% Catch-up Coverage** | |  | **15% Catch-up Coverage** | |
|  |  | Absolute Reduction^2^ | Relative Reduction^3^ |  | Absolute Reduction | Relative Reduction |  | Absolute Reduction | Relative Reduction |
| 0.5 Years | 10% | 1,668,248 | 0·6 |  | 1,715,621 | 0·6 |  | 1,761,486 | 0·6 |
|  | 20% | 3,656,528 | 1·3 |  | 3,752,270 | 1·3 |  | 3,845,131 | 1·4 |
|  | 30% | 5,644,965 | 2·0 |  | 5,790,027 | 2·1 |  | 5,931,697 | 2·1 |
|  | 40% | 7,641,905 | 2·7 |  | 7,833,866 | 2·8 |  | 8,022,650 | 2·9 |
|  | 50% | 9,645,759 | 3·5 |  | 9,887,379 | 3·5 |  | 10,122,036 | 3·6 |
|  | 60% | 11,653,841 | 4·2 |  | 11,945,728 | 4·3 |  | 12,230,057 | 4·4 |
|  | 70% | 13,666,455 | 4·9 |  | 14,007,670 | 5·0 |  | 14,340,640 | 5·1 |
|  | 80% | 15,682,634 | 5·6 |  | 16,073,981 | 5·8 |  | 16,455,666 | 5·9 |
|  | 90% | 17,703,512 | 6·4 |  | 18,143,971 | 6·5 |  | 18,574,550 | 6·7 |
| 1 Year | 10% | 3,096,973 | 1·1 |  | 3,185,093 | 1·1 |  | 3,270,276 | 1·2 |
|  | 20% | 6,807,030 | 2·4 |  | 6,985,603 | 2·5 |  | 7,157,272 | 2·6 |
|  | 30% | 10,528,518 | 3·8 |  | 10,798,584 | 3·9 |  | 11,058,064 | 4·0 |
|  | 40% | 14,259,960 | 5·1 |  | 14,621,337 | 5·2 |  | 14,969,286 | 5·4 |
|  | 50% | 17,998,348 | 6·5 |  | 18,450,817 | 6·6 |  | 18,889,929 | 6·8 |
|  | 60% | 21,748,071 | 7·8 |  | 22,293,120 | 8·0 |  | 22,822,425 | 8·2 |
|  | 70% | 25,501,937 | 9·2 |  | 26,143,096 | 9·4 |  | 26,759,293 | 9·6 |
|  | 80% | 29,242,609 | 10·5 |  | 29,977,813 | 10·8 |  | 30,690,116 | 11·0 |
|  | 90% | 32,977,968 | 11·8 |  | 33,804,006 | 12·1 |  | 34,607,031 | 12·4 |
| 5 Years | 10% | 10,762,285 | 3·9 |  | 11,059,851 | 4·0 |  | 11,344,444 | 4·1 |
|  | 20% | 23,133,037 | 8·3 |  | 23,725,921 | 8·5 |  | 24,294,005 | 8·7 |
|  | 30% | 35,370,729 | 12·7 |  | 36,261,281 | 13·0 |  | 37,112,019 | 13·3 |
|  | 40% | 47,379,547 | 17·0 |  | 48,556,540 | 17·4 |  | 49,679,759 | 17·9 |
|  | 50% | 59,024,084 | 21·2 |  | 60,474,255 | 21·7 |  | 61,860,942 | 22·2 |
|  | 60% | 70,288,310 | 25·2 |  | 71,997,887 | 25·8 |  | 73,630,318 | 26·4 |
|  | 70% | 80,869,251 | 29·1 |  | 82,800,397 | 29·7 |  | 84,640,781 | 30·4 |
|  | 80% | 90,821,768 | 32·6 |  | 92,937,586 | 33·4 |  | 94,950,126 | 34·1 |
|  | 90% | 100,072,340 | 36·0 |  | 102,347,168 | 36·8 |  | 104,507,402 | 37·6 |
| 10 Years | 10% | 15,695,733 | 5·6 |  | 16,118,918 | 5·8 |  | 16,523,069 | 5·9 |
|  | 20% | 33,131,312 | 11·9 |  | 33,968,006 | 12·2 |  | 34,767,007 | 12·5 |
|  | 30% | 49,980,931 | 18·0 |  | 51,218,106 | 18·4 |  | 52,398,979 | 18·8 |
|  | 40% | 66,005,708 | 23·7 |  | 67,608,737 | 24·3 |  | 69,135,819 | 24·8 |
|  | 50% | 80,939,554 | 29·1 |  | 82,845,065 | 29·7 |  | 84,655,424 | 30·4 |
|  | 60% | 94,488,865 | 33·9 |  | 96,639,426 | 34·7 |  | 98,668,848 | 35·4 |
|  | 70% | 106,543,186 | 38·3 |  | 108,837,005 | 39·1 |  | 110,994,293 | 39·9 |
|  | 80% | 116,970,848 | 42·1 |  | 119,331,079 | 42·9 |  | 121,535,059 | 43·7 |
|  | 90% | 125,844,834 | 45·3 |  | 128,215,507 | 46·1 |  | 130,422,757 | 46·9 |
| 20 Years | 10% | 20,249,613 | 7·3 |  | 20,790,316 | 7·5 |  | 21,306,073 | 7·6 |
|  | 20% | 42,004,201 | 15·1 |  | 43,063,132 | 15·5 |  | 44,072,489 | 15·8 |
|  | 30% | 62,430,046 | 22·4 |  | 63,950,062 | 23·0 |  | 65,395,583 | 23·5 |
|  | 40% | 81,039,627 | 29·2 |  | 82,937,049 | 29·8 |  | 84,735,608 | 30·5 |
|  | 50% | 97,578,494 | 35·1 |  | 99,735,848 | 35·8 |  | 101,768,353 | 36·6 |
|  | 60% | 111,499,819 | 40·1 |  | 113,783,115 | 40·9 |  | 115,917,859 | 41·7 |
|  | 70% | 123,017,009 | 44·3 |  | 125,321,340 | 45·1 |  | 127,457,649 | 45·9 |
|  | 80% | 132,389,826 | 47·6 |  | 134,659,264 | 48·5 |  | 136,745,183 | 49·2 |
|  | 90% | 140,072,026 | 50·4 |  | 142,226,134 | 51·1 |  | 144,184,292 | 51·9 |

^1^ Scenario C: Scenario A at 50% routine coverage + catch-up campaign among unvaccinated 15-24-year-old females in 2035 at 5/10/15% coverage

^2^ Refers to difference in total CT infections comparing Scenario C to the referent scenario (no vaccination) from 2025-2075.

^3^ Refers to percent reduction in total CT infections comparing Scenario C to the referent scenario (no vaccination) from 2025-2075.

Abbreviations: CT, *Chlamydia trachomatis*; VE, vaccine efficacy.

| **Table S16**. Absolute and relative reduction in CT infections between 2025-2075, comparing Scenario D^1^ at 50% routine coverage to No Vaccination by catch-up coverage, efficacy, and duration of protection. | | | | | | | | | |
| --- | --- | --- | --- | --- | --- | --- | --- | --- | --- |
| **Duration of Protection** | **VE** | **5% Catch-up Coverage** | |  | **10% Catch-up Coverage** | |  | **15% Catch-up Coverage** | |
|  |  | Absolute Reduction^2^ | Relative Reduction^3^ |  | Absolute Reduction | Relative Reduction |  | Absolute Reduction | Relative Reduction |
| 0.5 Years | 10% | 3,290,959 | 1·2 |  | 3,338,288 | 1·2 |  | 3,384,281 | 1·2 |
|  | 20% | 7,116,963 | 2·6 |  | 7,212,561 | 2·6 |  | 7,305,841 | 2·6 |
|  | 30% | 10,928,765 | 3·9 |  | 11,072,224 | 4·0 |  | 11,211,862 | 4·0 |
|  | 40% | 14,725,062 | 5·3 |  | 14,917,519 | 5·4 |  | 15,104,957 | 5·4 |
|  | 50% | 18,502,814 | 6·6 |  | 18,743,647 | 6·7 |  | 18,978,274 | 6·8 |
|  | 60% | 22,261,910 | 8·0 |  | 22,549,307 | 8·1 |  | 22,831,127 | 8·2 |
|  | 70% | 26,002,407 | 9·3 |  | 26,338,182 | 9·5 |  | 26,667,682 | 9·6 |
|  | 80% | 29,719,856 | 10·7 |  | 30,103,816 | 10·8 |  | 30,480,024 | 10·9 |
|  | 90% | 33,411,496 | 12·0 |  | 33,842,758 | 12·1 |  | 34,263,699 | 12·3 |
| 1 Year | 10% | 6,108,618 | 2·2 |  | 6,197,139 | 2·2 |  | 6,282,925 | 2·3 |
|  | 20% | 13,240,908 | 4·8 |  | 13,417,659 | 4·8 |  | 13,588,220 | 4·9 |
|  | 30% | 20,302,563 | 7·3 |  | 20,570,683 | 7·4 |  | 20,829,559 | 7·5 |
|  | 40% | 27,279,767 | 9·8 |  | 27,635,466 | 9·9 |  | 27,979,351 | 10·1 |
|  | 50% | 34,165,445 | 12·3 |  | 34,608,965 | 12·4 |  | 35,039,602 | 12·6 |
|  | 60% | 40,968,899 | 14·7 |  | 41,497,500 | 14·9 |  | 42,009,003 | 15·1 |
|  | 70% | 47,627,520 | 17·1 |  | 48,238,234 | 17·3 |  | 48,829,426 | 17·5 |
|  | 80% | 54,181,482 | 19·4 |  | 54,873,150 | 19·7 |  | 55,543,147 | 19·9 |
|  | 90% | 60,591,699 | 21·7 |  | 61,363,872 | 22·0 |  | 62,111,711 | 22·3 |
| 5 Years | 10% | 20,993,837 | 7·5 |  | 21,286,539 | 7·6 |  | 21,566,260 | 7·7 |
|  | 20% | 43,976,438 | 15·8 |  | 44,549,552 | 16·0 |  | 45,096,780 | 16·2 |
|  | 30% | 65,211,381 | 23·4 |  | 66,030,741 | 23·7 |  | 66,815,532 | 24·0 |
|  | 40% | 84,367,092 | 30·3 |  | 85,392,574 | 30·7 |  | 86,371,240 | 31·0 |
|  | 50% | 100,922,868 | 36·3 |  | 102,085,063 | 36·7 |  | 103,191,195 | 37·1 |
|  | 60% | 114,870,495 | 41·3 |  | 116,108,123 | 41·7 |  | 117,281,740 | 42·1 |
|  | 70% | 126,255,594 | 45·4 |  | 127,506,288 | 45·9 |  | 128,692,362 | 46·3 |
|  | 80% | 135,568,585 | 48·8 |  | 136,799,617 | 49·2 |  | 137,956,774 | 49·6 |
|  | 90% | 143,031,245 | 51·5 |  | 144,211,352 | 51·9 |  | 145,315,399 | 52·3 |
| 10 Years | 10% | 30,357,511 | 10·9 |  | 30,769,178 | 11·0 |  | 31,162,233 | 11·2 |
|  | 20% | 61,396,734 | 22·1 |  | 62,175,516 | 22·4 |  | 62,918,449 | 22·6 |
|  | 30% | 88,022,053 | 31·6 |  | 89,064,818 | 32·0 |  | 90,056,859 | 32·4 |
|  | 40% | 109,454,698 | 39·3 |  | 110,637,325 | 39·8 |  | 111,757,243 | 40·2 |
|  | 50% | 125,566,658 | 45·2 |  | 126,766,725 | 45·6 |  | 127,899,329 | 46·0 |
|  | 60% | 137,641,383 | 49·5 |  | 138,808,388 | 49·9 |  | 139,900,887 | 50·3 |
|  | 70% | 146,564,401 | 52·7 |  | 147,639,675 | 53·1 |  | 148,639,794 | 53·5 |
|  | 80% | 153,228,906 | 55·1 |  | 154,208,949 | 55·5 |  | 155,115,525 | 55·8 |
|  | 90% | 158,340,455 | 57·0 |  | 159,219,740 | 57·3 |  | 160,028,794 | 57·6 |
| 20 Years | 10% | 38,709,811 | 13·9 |  | 39,230,441 | 14·1 |  | 39,726,718 | 14·3 |
|  | 20% | 75,603,461 | 27·2 |  | 76,529,605 | 27·5 |  | 77,410,821 | 27·8 |
|  | 30% | 104,473,641 | 37·5 |  | 105,613,311 | 37·9 |  | 106,696,575 | 38·3 |
|  | 40% | 124,959,816 | 45·0 |  | 126,127,419 | 45·4 |  | 127,224,527 | 45·8 |
|  | 50% | 139,004,987 | 50·0 |  | 140,109,650 | 50·4 |  | 141,140,876 | 50·8 |
|  | 60% | 148,754,512 | 53·5 |  | 149,743,701 | 53·8 |  | 150,659,511 | 54·2 |
|  | 70% | 155,611,426 | 56·0 |  | 156,480,648 | 56·3 |  | 157,281,487 | 56·6 |
|  | 80% | 160,669,384 | 57·8 |  | 161,436,606 | 58·1 |  | 162,140,193 | 58·3 |
|  | 90% | 164,578,875 | 59·2 |  | 165,257,229 | 59·5 |  | 165,877,002 | 59·7 |

^1^ Scenario D: Scenario B @ 50% routine coverage + catch-up campaign among unvaccinated 15-24-year-old females in 2035 at 5/10/15% coverage

^2^ Refers to difference in total CT infections comparing Scenario D to the referent scenario (no vaccination) from 2025-2075.

^3^ Refers to percent reduction in total CT infections comparing Scenario D to the referent scenario (no vaccination) from 2025-2075. Abbreviations: CT, *Chlamydia trachomatis*; VE, vaccine efficacy.

|  | **Table S17.** CT incidence rates among all individuals aged 15-64 years between 2025-2075 for Scenario A ^1,2,3,4^ | | | | | | | | | | | | | | | | | | |
| --- | --- | --- | --- | --- | --- | --- | --- | --- | --- | --- | --- | --- | --- | --- | --- | --- | --- | --- | --- |
| **Duration of Protection** | | **VE** |  | **30% Coverage** | | | |  | | **50% Coverage** | | |  | |  | | **70% Coverage** | | |
|  |  |  |  | 2025 | 2050 | 2075 |  | | 2025 | | 2050 | 2075 | |  | | 2025 | | 2050 | 2075 |
| 0·5 Years | | 10% |  | 16·9 | 18·4 | 18·5 |  | | 16·9 | | 18·3 | 18·4 | |  | | 16·9 | | 18·2 | 18·3 |
|  |  | 20% |  | 16·9 | 18·3 | 18·4 |  | | 16·9 | | 18·1 | 18·2 | |  | | 16·9 | | 18·0 | 18·0 |
|  |  | 30% |  | 16·9 | 18·1 | 18·2 |  | | 16·9 | | 17·9 | 18·0 | |  | | 16·9 | | 17·7 | 17·7 |
|  |  | 40% |  | 16·9 | 18·0 | 18·1 |  | | 16·9 | | 17·7 | 17·7 | |  | | 16·8 | | 17·5 | 17·3 |
|  |  | 50% |  | 16·9 | 17·9 | 17·9 |  | | 16·8 | | 17·6 | 17·5 | |  | | 16·8 | | 17·2 | 17·0 |
|  |  | 60% |  | 16·9 | 17·8 | 17·8 |  | | 16·8 | | 17·4 | 17·2 | |  | | 16·8 | | 16·9 | 16·6 |
|  |  | 70% |  | 16·9 | 17·7 | 17·7 |  | | 16·8 | | 17·2 | 17·0 | |  | | 16·8 | | 16·7 | 16·3 |
|  |  | 80% |  | 16·8 | 17·6 | 17·5 |  | | 16·8 | | 17·0 | 16·7 | |  | | 16·8 | | 16·4 | 15·9 |
|  |  | 90% |  | 16·8 | 17·4 | 17·3 |  | | 16·8 | | 16·8 | 16·5 | |  | | 16·8 | | 16·1 | 15·6 |
| 1 Year | | 10% |  | 16·9 | 18·3 | 18·4 |  | | 16·9 | | 18·2 | 18·2 | |  | | 16·9 | | 18·1 | 18·1 |
|  |  | 20% |  | 16·9 | 18·1 | 18·1 |  | | 16·9 | | 17·8 | 17·8 | |  | | 16·9 | | 17·6 | 17·4 |
|  |  | 30% |  | 16·9 | 17·9 | 17·9 |  | | 16·9 | | 17·5 | 17·3 | |  | | 16·8 | | 17·1 | 16·8 |
|  |  | 40% |  | 16·9 | 17·7 | 17·6 |  | | 16·8 | | 17·1 | 16·9 | |  | | 16·8 | | 16·6 | 16·1 |
|  |  | 50% |  | 16·9 | 17·4 | 17·3 |  | | 16·8 | | 16·8 | 16·4 | |  | | 16·8 | | 16·1 | 15·5 |
|  |  | 60% |  | 16·8 | 17·2 | 17·0 |  | | 16·8 | | 16·4 | 15·9 | |  | | 16·8 | | 15·6 | 14·8 |
|  |  | 70% |  | 16·8 | 17·0 | 16·7 |  | | 16·8 | | 16·0 | 15·4 | |  | | 16·8 | | 15·1 | 14·2 |
|  |  | 80% |  | 16·8 | 16·8 | 16·4 |  | | 16·8 | | 15·7 | 15·0 | |  | | 16·8 | | 14·6 | 13·5 |
|  |  | 90% |  | 16·8 | 16·6 | 16·2 |  | | 16·8 | | 15·3 | 14·5 | |  | | 16·8 | | 14·2 | 12·9 |
| 5 Years | | 10% |  | 16·9 | 17·9 | 17·7 |  | | 16·9 | | 17·5 | 17·1 | |  | | 16·9 | | 17·1 | 16·5 |
|  |  | 20% |  | 16·9 | 17·2 | 16·8 |  | | 16·9 | | 16·3 | 15·5 | |  | | 16·9 | | 15·5 | 14·2 |
|  |  | 30% |  | 16·9 | 16·4 | 15·8 |  | | 16·8 | | 15·1 | 13·8 | |  | | 16·8 | | 13·9 | 12·0 |
|  |  | 40% |  | 16·9 | 15·7 | 14·7 |  | | 16·8 | | 14·0 | 12·2 | |  | | 16·8 | | 12·5 | 9·8 |
|  |  | 50% |  | 16·8 | 15·0 | 13·7 |  | | 16·8 | | 12·9 | 10·7 | |  | | 16·8 | | 11·1 | 7·8 |
|  |  | 60% |  | 16·8 | 14·3 | 12·8 |  | | 16·8 | | 11·9 | 9·1 | |  | | 16·8 | | 9·8 | 6·1 |
|  |  | 70% |  | 16·8 | 13·6 | 11·8 |  | | 16·8 | | 10·9 | 7·7 | |  | | 16·8 | | 8·6 | 4·6 |
|  |  | 80% |  | 16·8 | 13·0 | 10·8 |  | | 16·8 | | 9·9 | 6·4 | |  | | 16·8 | | 7·5 | 3·3 |
|  |  | 90% |  | 16·8 | 12·3 | 9·9 |  | | 16·8 | | 9·0 | 5·3 | |  | | 16·8 | | 6·5 | 2·4 |
| 10 Year | | 10% |  | 16·9 | 17·6 | 17·3 |  | | 16·9 | | 17·0 | 16·3 | |  | | 16·9 | | 16·5 | 15·4 |
|  |  | 20% |  | 16·9 | 16·6 | 15·8 |  | | 16·9 | | 15·4 | 13·9 | |  | | 16·9 | | 14·3 | 12·1 |
|  |  | 30% |  | 16·9 | 15·6 | 14·3 |  | | 16·8 | | 13·8 | 11·6 | |  | | 16·8 | | 12·2 | 9·0 |
|  |  | 40% |  | 16·9 | 14·6 | 12·9 |  | | 16·8 | | 12·4 | 9·3 | |  | | 16·8 | | 10·3 | 6·3 |
|  |  | 50% |  | 16·8 | 13·7 | 11·5 |  | | 16·8 | | 10·9 | 7·3 | |  | | 16·8 | | 8·6 | 4·1 |
|  |  | 60% |  | 16·8 | 12·7 | 10·1 |  | | 16·8 | | 9·6 | 5·5 | |  | | 16·8 | | 7·2 | 2·6 |
|  |  | 70% |  | 16·8 | 11·8 | 8·7 |  | | 16·8 | | 8·4 | 4·0 | |  | | 16·8 | | 5·9 | 1·5 |
|  |  | 80% |  | 16·8 | 11·0 | 7·5 |  | | 16·8 | | 7·4 | 2·9 | |  | | 16·8 | | 4·8 | 0·8 |
|  |  | 90% |  | 16·8 | 10·1 | 6·3 |  | | 16·8 | | 6·4 | 2·0 | |  | | 16·8 | | 3·9 | 0·5 |
| 20 Years | | 10% |  | 16·9 | 17·4 | 16·8 |  | | 16·9 | | 16·6 | 15·5 | |  | | 16·9 | | 16·0 | 14·3 |
|  |  | 20% |  | 16·9 | 16·1 | 14·9 |  | | 16·9 | | 14·6 | 12·4 | |  | | 16·9 | | 13·3 | 10·1 |
|  |  | 30% |  | 16·9 | 14·9 | 13·0 |  | | 16·8 | | 12·8 | 9·5 | |  | | 16·8 | | 10·9 | 6·5 |
|  |  | 40% |  | 16·9 | 13·7 | 11·1 |  | | 16·8 | | 11·0 | 6·9 | |  | | 16·8 | | 8·7 | 3·8 |
|  |  | 50% |  | 16·8 | 12·6 | 9·3 |  | | 16·8 | | 9·4 | 4·7 | |  | | 16·8 | | 6·9 | 2·0 |
|  |  | 60% |  | 16·8 | 11·5 | 7·7 |  | | 16·8 | | 8·0 | 3·1 | |  | | 16·8 | | 5·4 | 1·0 |
|  |  | 70% |  | 16·8 | 10·4 | 6·2 |  | | 16·8 | | 6·7 | 1·9 | |  | | 16·8 | | 4·2 | 0·4 |
|  |  | 80% |  | 16·8 | 9·4 | 4·9 |  | | 16·8 | | 5·6 | 1·1 | |  | | 16·8 | | 3·2 | 0·2 |
|  |  | 90% |  | 16·8 | 8·5 | 3·8 |  | | 16·8 | | 4·6 | 0·6 | |  | | 16·8 | | 2·4 | 0·1 |

^1^ IR estimated as # of incident infections per 1,000 person-years (PY)

^2^ IR estimates are for the total population (males and females)

^3^ Under no vaccination, IRs in 2025, 2050, and 2075 were 16·9, 18·5, and 18·7/1000 PY, respectively

^4^ Scenario A: Vaccinating only 15-year-old females entering the model between 2025-2075.

Abbreviations: CT, *Chlamydia trachomatis*, IR, incidence rate; PY, person-years

|  | **Table S18.** CT incidence rates among all individuals aged 15-64 years between 2025-2075 for Scenario B ^1,2,3,4^ | | | | | | | | | | | | | | | | | | |
| --- | --- | --- | --- | --- | --- | --- | --- | --- | --- | --- | --- | --- | --- | --- | --- | --- | --- | --- | --- |
| **Duration of Protection** | | **VE** |  | **30% Coverage** | | | |  | | **50% Coverage** | | |  | |  | | **70% Coverage** | | |
|  |  |  |  | 2025 | 2050 | 2075 |  | | 2025 | | 2050 | 2075 | |  | | 2025 | | 2050 | 2075 |
| 0·5 Years | | 10% |  | 16·9 | 18·3 | 18·4 |  | | 16·9 | | 18·2 | 18·2 | |  | | 16·9 | | 18·0 | 18·0 |
|  |  | 20% |  | 16·9 | 18·1 | 18·1 |  | | 16·9 | | 17·8 | 17·7 | |  | | 16·9 | | 17·5 | 17·4 |
|  |  | 30% |  | 16·9 | 17·8 | 17·8 |  | | 16·8 | | 17·4 | 17·3 | |  | | 16·8 | | 17·0 | 16·7 |
|  |  | 40% |  | 16·9 | 17·6 | 17·5 |  | | 16·8 | | 17·1 | 16·8 | |  | | 16·8 | | 16·5 | 16·0 |
|  |  | 50% |  | 16·8 | 17·4 | 17·3 |  | | 16·8 | | 16·7 | 16·3 | |  | | 16·8 | | 16·0 | 15·4 |
|  |  | 60% |  | 16·8 | 17·2 | 17·0 |  | | 16·8 | | 16·3 | 15·8 | |  | | 16·8 | | 15·5 | 14·7 |
|  |  | 70% |  | 16·8 | 16·9 | 16·7 |  | | 16·8 | | 16·0 | 15·4 | |  | | 16·8 | | 15·0 | 14·0 |
|  |  | 80% |  | 16·8 | 16·7 | 16·4 |  | | 16·8 | | 15·6 | 14·9 | |  | | 16·8 | | 14·6 | 13·4 |
|  |  | 90% |  | 16·8 | 16·5 | 16·1 |  | | 16·8 | | 15·3 | 14·4 | |  | | 16·8 | | 14·1 | 12·8 |
| 1 Year | | 10% |  | 16·9 | 18·1 | 18·2 |  | | 16·9 | | 17·9 | 17·8 | |  | | 16·9 | | 17·7 | 17·5 |
|  |  | 20% |  | 16·9 | 17·7 | 17·6 |  | | 16·8 | | 17·2 | 16·9 | |  | | 16·8 | | 16·7 | 16·2 |
|  |  | 30% |  | 16·9 | 17·3 | 17·1 |  | | 16·8 | | 16·5 | 16·0 | |  | | 16·8 | | 15·8 | 15·0 |
|  |  | 40% |  | 16·8 | 16·9 | 16·5 |  | | 16·8 | | 15·9 | 15·1 | |  | | 16·8 | | 14·9 | 13·7 |
|  |  | 50% |  | 16·8 | 16·5 | 16·0 |  | | 16·8 | | 15·2 | 14·2 | |  | | 16·8 | | 14·0 | 12·5 |
|  |  | 60% |  | 16·8 | 16·1 | 15·4 |  | | 16·8 | | 14·6 | 13·3 | |  | | 16·8 | | 13·2 | 11·3 |
|  |  | 70% |  | 16·8 | 15·7 | 14·9 |  | | 16·8 | | 13·9 | 12·5 | |  | | 16·8 | | 12·4 | 10·2 |
|  |  | 80% |  | 16·8 | 15·2 | 14·3 |  | | 16·8 | | 13·3 | 11·6 | |  | | 16·8 | | 11·6 | 9·1 |
|  |  | 90% |  | 16·8 | 14·8 | 13·8 |  | | 16·8 | | 12·7 | 10·8 | |  | | 16·8 | | 10·8 | 8·1 |
| 5 Years | | 10% |  | 16·9 | 17·3 | 16·8 |  | | 16·9 | | 16·5 | 15·6 | |  | | 16·9 | | 15·8 | 14·4 |
|  |  | 20% |  | 16·9 | 15·9 | 14·9 |  | | 16·8 | | 14·3 | 12·5 | |  | | 16·8 | | 12·9 | 10·2 |
|  |  | 30% |  | 16·8 | 14·6 | 13·0 |  | | 16·8 | | 12·3 | 9·6 | |  | | 16·8 | | 10·3 | 6·6 |
|  |  | 40% |  | 16·8 | 13·3 | 11·2 |  | | 16·8 | | 10·5 | 7·0 | |  | | 16·8 | | 8·2 | 3·9 |
|  |  | 50% |  | 16·8 | 12·1 | 9·4 |  | | 16·8 | | 8·9 | 4·9 | |  | | 16·8 | | 6·4 | 2·2 |
|  |  | 60% |  | 16·8 | 11·0 | 7·8 |  | | 16·8 | | 7·5 | 3·3 | |  | | 16·8 | | 5·0 | 1·1 |
|  |  | 70% |  | 16·8 | 9·9 | 6·4 |  | | 16·8 | | 6·3 | 2·1 | |  | | 16·8 | | 3·9 | 0·6 |
|  |  | 80% |  | 16·8 | 9·0 | 5·1 |  | | 16·8 | | 5·2 | 1·3 | |  | | 16·8 | | 3·0 | 0·3 |
|  |  | 90% |  | 16·8 | 8·0 | 4·0 |  | | 16·8 | | 4·3 | 0·8 | |  | | 16·8 | | 2·3 | 0·1 |
| 10 Year | | 10% |  | 16·9 | 16·8 | 15·9 |  | | 16·9 | | 15·7 | 14·1 | |  | | 16·9 | | 14·6 | 12.3 |
|  |  | 20% |  | 16·9 | 14·9 | 13·1 |  | | 16·8 | | 12·7 | 9·7 | |  | | 16·8 | | 10·9 | 6.8 |
|  |  | 30% |  | 16·8 | 13·1 | 10·4 |  | | 16·8 | | 10·2 | 6·1 | |  | | 16·8 | | 7·9 | 3.1 |
|  |  | 40% |  | 16·8 | 11·5 | 8·0 |  | | 16·8 | | 8·1 | 3·4 | |  | | 16·8 | | 5·6 | 1.2 |
|  |  | 50% |  | 16·8 | 10·0 | 5·9 |  | | 16·8 | | 6·3 | 1·8 | |  | | 16·8 | | 3·9 | 0.4 |
|  |  | 60% |  | 16·8 | 8·6 | 4·2 |  | | 16·8 | | 4·9 | 0·9 | |  | | 16·8 | | 2·7 | 0.2 |
|  |  | 70% |  | 16·8 | 7·4 | 2·9 |  | | 16·8 | | 3·7 | 0·4 | |  | | 16·8 | | 1·9 | 0.1 |
|  |  | 80% |  | 16·8 | 6·4 | 2·0 |  | | 16·8 | | 2·9 | 0·2 | |  | | 16·8 | | 1·4 | 0.0 |
|  |  | 90% |  | 16·8 | 5·4 | 1·3 |  | | 16·8 | | 2·2 | 0·1 | |  | | 16·8 | | 1·0 | 0.0 |
| 20 Years | | 10% |  | 16·9 | 16·3 | 15·0 |  | | 16·9 | | 15·0 | 12·6 | |  | | 16·9 | | 13·7 | 10·4 |
|  |  | 20% |  | 16·9 | 14·0 | 11·4 |  | | 16·8 | | 11·5 | 7·3 | |  | | 16·8 | | 9·4 | 4·3 |
|  |  | 30% |  | 16·8 | 11·9 | 8·2 |  | | 16·8 | | 8·6 | 3·6 | |  | | 16·8 | | 6·2 | 1·3 |
|  |  | 40% |  | 16·8 | 10·0 | 5·5 |  | | 16·8 | | 6·4 | 1·6 | |  | | 16·8 | | 3·9 | 0·4 |
|  |  | 50% |  | 16·8 | 8·4 | 3·5 |  | | 16·8 | | 4·6 | 0·6 | |  | | 16·8 | | 2·5 | 0·1 |
|  |  | 60% |  | 16·8 | 7·0 | 2·1 |  | | 16·8 | | 3·3 | 0·2 | |  | | 16·8 | | 1·6 | 0·0 |
|  |  | 70% |  | 16·8 | 5·7 | 1·2 |  | | 16·8 | | 2·4 | 0·1 | |  | | 16·8 | | 1·1 | 0·0 |
|  |  | 80% |  | 16·8 | 4·7 | 0·7 |  | | 16·8 | | 1·8 | 0·0 | |  | | 16·8 | | 0·7 | 0·0 |
|  |  | 90% |  | 16·8 | 3·9 | 0·4 |  | | 16·8 | | 1·3 | 0·0 | |  | | 16·8 | | 0·5 | 0·0 |

^1^ IR estimated as # of incident infections per 1,000 person-years (PY)

^2^ IR estimates are for the total population (males and females)

^3^ Under no vaccination, IRs in 2025, 2050, and 2075 were 16·9, 18·5, and 18·7/1,000 PY, respectively

^4^ Scenario B: Vaccinating 15-year-old females and males entering the model between 2025-2075.

Abbreviations: CT, *Chlamydia trachomatis*, IR, incidence rate; PY, person-years

|  | **Table S19.** CT incidence rates among all individuals aged 15-64 years between 2025-2075 for Scenario C ^1,2,3,4^ | | | | | | | | | | | | | | | | | | |
| --- | --- | --- | --- | --- | --- | --- | --- | --- | --- | --- | --- | --- | --- | --- | --- | --- | --- | --- | --- |
| **Duration of Protection** | | **VE** |  | **5% Coverage** | | | |  | | **10% Coverage** | | |  | |  | | **15% Coverage** | | |
|  |  |  |  | 2025 | 2050 | 2075 |  | | 2025 | | 2050 | 2075 | |  | | 2025 | | 2050 | 2075 |
| 0·5 Years | | 10% |  | 16·9 | 18·3 | 18·4 |  | | 16·9 | | 18·3 | 18·4 | |  | | 16·9 | | 18·3 | 18·4 |
|  |  | 20% |  | 16·9 | 18·1 | 18·2 |  | | 16·9 | | 18·1 | 18·2 | |  | | 16·9 | | 18·1 | 18·2 |
|  |  | 30% |  | 16·9 | 17·9 | 18·0 |  | | 16·9 | | 17·9 | 18·0 | |  | | 16·9 | | 17·9 | 17·9 |
|  |  | 40% |  | 16·9 | 17·7 | 17·7 |  | | 16·9 | | 17·7 | 17·7 | |  | | 16·9 | | 17·7 | 17·7 |
|  |  | 50% |  | 16·8 | 17·5 | 17·5 |  | | 16·8 | | 17·5 | 17·5 | |  | | 16·8 | | 17·5 | 17·5 |
|  |  | 60% |  | 16·8 | 17·3 | 17·2 |  | | 16·8 | | 17·3 | 17·2 | |  | | 16·8 | | 17·3 | 17·2 |
|  |  | 70% |  | 16·8 | 17·1 | 17·0 |  | | 16·8 | | 17·1 | 17·0 | |  | | 16·8 | | 17·1 | 17·0 |
|  |  | 80% |  | 16·8 | 16·9 | 16·7 |  | | 16·8 | | 16·9 | 16·7 | |  | | 16·8 | | 16·8 | 16·7 |
|  |  | 90% |  | 16·8 | 16·7 | 16·5 |  | | 16·8 | | 16·7 | 16·5 | |  | | 16·8 | | 16·6 | 16·4 |
| 1 Year | | 10% |  | 16·9 | 18·2 | 18·2 |  | | 16·9 | | 18·2 | 18·2 | |  | | 16·9 | | 18·1 | 18·2 |
|  |  | 20% |  | 16·9 | 17·8 | 17·8 |  | | 16·9 | | 17·8 | 17·8 | |  | | 16·9 | | 17·8 | 17·8 |
|  |  | 30% |  | 16·9 | 17·4 | 17·3 |  | | 16·9 | | 17·4 | 17·3 | |  | | 16·9 | | 17·4 | 17·3 |
|  |  | 40% |  | 16·8 | 17·1 | 16·9 |  | | 16·8 | | 17·0 | 16·8 | |  | | 16·8 | | 17·0 | 16·8 |
|  |  | 50% |  | 16·8 | 16·7 | 16·4 |  | | 16·8 | | 16·7 | 16·4 | |  | | 16·8 | | 16·6 | 16·4 |
|  |  | 60% |  | 16·8 | 16·3 | 15·9 |  | | 16·8 | | 16·3 | 15·9 | |  | | 16·8 | | 16·2 | 15·9 |
|  |  | 70% |  | 16·8 | 16·0 | 15·4 |  | | 16·8 | | 15·9 | 15·4 | |  | | 16·8 | | 15·8 | 15·4 |
|  |  | 80% |  | 16·8 | 15·6 | 14·9 |  | | 16·8 | | 15·5 | 14·9 | |  | | 16·8 | | 15·4 | 14·9 |
|  |  | 90% |  | 16·8 | 15·2 | 14·5 |  | | 16·8 | | 15·1 | 14·4 | |  | | 16·8 | | 15·0 | 14·4 |
| 5 Years | | 10% |  | 16·9 | 17·4 | 17·1 |  | | 16·9 | | 17·4 | 17·1 | |  | | 16·9 | | 17·3 | 17·1 |
|  |  | 20% |  | 16·9 | 16·2 | 15·5 |  | | 16·9 | | 16·1 | 15·4 | |  | | 16·9 | | 16·1 | 15·4 |
|  |  | 30% |  | 16·8 | 15·0 | 13·8 |  | | 16·8 | | 14·9 | 13·8 | |  | | 16·8 | | 14·8 | 13·8 |
|  |  | 40% |  | 16·8 | 13·9 | 12·2 |  | | 16·8 | | 13·7 | 12·2 | |  | | 16·8 | | 13·5 | 12·1 |
|  |  | 50% |  | 16·8 | 12·7 | 10·6 |  | | 16·8 | | 12·5 | 10·5 | |  | | 16·8 | | 12·4 | 10·5 |
|  |  | 60% |  | 16·8 | 11·7 | 9·1 |  | | 16·8 | | 11·4 | 9·0 | |  | | 16·8 | | 11·2 | 8·9 |
|  |  | 70% |  | 16·8 | 10·6 | 7·6 |  | | 16·8 | | 10·4 | 7·6 | |  | | 16·8 | | 10·1 | 7·5 |
|  |  | 80% |  | 16·8 | 9·6 | 6·3 |  | | 16·8 | | 9·4 | 6·2 | |  | | 16·8 | | 9·1 | 6·1 |
|  |  | 90% |  | 16·8 | 8·7 | 5·2 |  | | 16·8 | | 8·4 | 5·1 | |  | | 16·8 | | 8·2 | 5·0 |
| 10 Year | | 10% |  | 16·9 | 17·0 | 16·3 |  | | 16·9 | | 16·9 | 16·3 | |  | | 16·9 | | 16·8 | 16·3 |
|  |  | 20% |  | 16·9 | 15·3 | 13·9 |  | | 16·9 | | 15·1 | 13·9 | |  | | 16·9 | | 15·0 | 13·8 |
|  |  | 30% |  | 16·8 | 13·6 | 11·5 |  | | 16·8 | | 13·5 | 11·5 | |  | | 16·8 | | 13·3 | 11·4 |
|  |  | 40% |  | 16·8 | 12·1 | 9·3 |  | | 16·8 | | 11·9 | 9·2 | |  | | 16·8 | | 11·7 | 9·1 |
|  |  | 50% |  | 16·8 | 10·7 | 7·2 |  | | 16·8 | | 10·4 | 7·1 | |  | | 16·8 | | 10·2 | 7·0 |
|  |  | 60% |  | 16·8 | 9·3 | 5·4 |  | | 16·8 | | 9·0 | 5·3 | |  | | 16·8 | | 8·8 | 5·2 |
|  |  | 70% |  | 16·8 | 8·1 | 3·9 |  | | 16·8 | | 7·8 | 3·8 | |  | | 16·8 | | 7·5 | 3·7 |
|  |  | 80% |  | 16·8 | 7·0 | 2·8 |  | | 16·8 | | 6·7 | 2·7 | |  | | 16·8 | | 6·4 | 2·6 |
|  |  | 90% |  | 16·8 | 6·0 | 1·9 |  | | 16·8 | | 5·7 | 1·8 | |  | | 16·8 | | 5·4 | 1·7 |
| 20 Years | | 10% |  | 16·9 | 16·6 | 15·5 |  | | 16·9 | | 16·5 | 15·5 | |  | | 16·9 | | 16·4 | 15·5 |
|  |  | 20% |  | 16·9 | 14·5 | 12·4 |  | | 16·9 | | 14·3 | 12·3 | |  | | 16·9 | | 14·2 | 12·3 |
|  |  | 30% |  | 16·8 | 12·5 | 9·4 |  | | 16·8 | | 12·3 | 9·3 | |  | | 16·8 | | 12·1 | 9·3 |
|  |  | 40% |  | 16·8 | 10·7 | 6·8 |  | | 16·8 | | 10·5 | 6·7 | |  | | 16·8 | | 10·2 | 6·6 |
|  |  | 50% |  | 16·8 | 9·1 | 4·6 |  | | 16·8 | | 8·8 | 4·5 | |  | | 16·8 | | 8·5 | 4·4 |
|  |  | 60% |  | 16·8 | 7·6 | 3·0 |  | | 16·8 | | 7·3 | 2·9 | |  | | 16·8 | | 7·0 | 2·8 |
|  |  | 70% |  | 16·8 | 6·4 | 1·8 |  | | 16·8 | | 6·0 | 1·7 | |  | | 16·8 | | 5·7 | 1·7 |
|  |  | 80% |  | 16·8 | 5·2 | 1·1 |  | | 16·8 | | 4·9 | 1·0 | |  | | 16·8 | | 4·6 | 0·9 |
|  |  | 90% |  | 16·8 | 4·3 | 0·6 |  | | 16·8 | | 4·0 | 0·6 | |  | | 16·8 | | 3·7 | 0·5 |

^1^ IR estimated as # of incident infections per 1,000 person-years (PY)

^2^ IR estimates are for the total population (males and females)

^3^ Under no vaccination, IRs in 2025, 2050, and 2075 were 16·9, 18·5, and 18·7/1,000 PY, respectively

^4^ Scenario C: Scenario A at 50% coverage + catch-up campaign among unvaccinated 15-24-year-old females in 2035 at 5/10/15% coverage

Abbreviations: CT, *Chlamydia trachomatis*, IR, incidence rate; PY, person-years

|  | **Table S20.** CT incidence rates among all individuals aged 15-64 years between 2025-2075 for Scenario D ^1,2,3,4^ | | | | | | | | | | | | | | | | | | |
| --- | --- | --- | --- | --- | --- | --- | --- | --- | --- | --- | --- | --- | --- | --- | --- | --- | --- | --- | --- |
| **Duration of Protection** | | **VE** |  | **5% Catch-up Coverage** | | | |  | | **10% Catch-up Coverage** | | |  | |  | | **15% Catch-up Coverage** | | |
|  |  |  |  | 2025 | 2050 | 2075 |  | | 2025 | | 2050 | 2075 | |  | | 2025 | | 2050 | 2075 |
| 0·5 Years | | 10% |  | 16·9 | 18·2 | 18·2 |  | | 16·9 | | 18·1 | 18·2 | |  | | 16·9 | | 18·1 | 18·2 |
|  |  | 20% |  | 16·9 | 17·8 | 17·7 |  | | 16·9 | | 17·8 | 17·7 | |  | | 16·9 | | 17·8 | 17·7 |
|  |  | 30% |  | 16·8 | 17·4 | 17·3 |  | | 16·8 | | 17·4 | 17·3 | |  | | 16·8 | | 17·4 | 17·3 |
|  |  | 40% |  | 16·8 | 17·0 | 16·8 |  | | 16·8 | | 17·0 | 16·8 | |  | | 16·8 | | 17·0 | 16·8 |
|  |  | 50% |  | 16·8 | 16·7 | 16·3 |  | | 16·8 | | 16·6 | 16·3 | |  | | 16·8 | | 16·6 | 16·3 |
|  |  | 60% |  | 16·8 | 16·3 | 15·8 |  | | 16·8 | | 16·3 | 15·8 | |  | | 16·8 | | 16·2 | 15·8 |
|  |  | 70% |  | 16·8 | 15·9 | 15·3 |  | | 16·8 | | 15·9 | 15·3 | |  | | 16·8 | | 15·9 | 15·3 |
|  |  | 80% |  | 16·8 | 15·6 | 14·9 |  | | 16·8 | | 15·5 | 14·9 | |  | | 16·8 | | 15·5 | 14·8 |
|  |  | 90% |  | 16·8 | 15·2 | 14·4 |  | | 16·8 | | 15·2 | 14·4 | |  | | 16·8 | | 15·1 | 14·4 |
| 1 Year | | 10% |  | 16·9 | 17·9 | 17·8 |  | | 16·9 | | 17·9 | 17·8 | |  | | 16·9 | | 17·9 | 17·8 |
|  |  | 20% |  | 16·8 | 17·2 | 16·9 |  | | 16·8 | | 17·2 | 16·9 | |  | | 16·8 | | 17·1 | 16·9 |
|  |  | 30% |  | 16·8 | 16·5 | 16·0 |  | | 16·8 | | 16·5 | 16·0 | |  | | 16·8 | | 16·4 | 16·0 |
|  |  | 40% |  | 16·8 | 15·8 | 15·1 |  | | 16·8 | | 15·8 | 15·1 | |  | | 16·8 | | 15·7 | 15·1 |
|  |  | 50% |  | 16·8 | 15·1 | 14·2 |  | | 16·8 | | 15·1 | 14·2 | |  | | 16·8 | | 15·0 | 14·2 |
|  |  | 60% |  | 16·8 | 14·5 | 13·3 |  | | 16·8 | | 14·4 | 13·3 | |  | | 16·8 | | 14·4 | 13·3 |
|  |  | 70% |  | 16·8 | 13·9 | 12·4 |  | | 16·8 | | 13·8 | 12·4 | |  | | 16·8 | | 13·7 | 12·4 |
|  |  | 80% |  | 16·8 | 13·2 | 11·6 |  | | 16·8 | | 13·2 | 11·6 | |  | | 16·8 | | 13·1 | 11·6 |
|  |  | 90% |  | 16·8 | 12·6 | 10·8 |  | | 16·8 | | 12·5 | 10·7 | |  | | 16·8 | | 12·5 | 10·7 |
| 5 Years | | 10% |  | 16·9 | 16·5 | 15·6 |  | | 16·9 | | 16·4 | 15·6 | |  | | 16·9 | | 16·4 | 15·5 |
|  |  | 20% |  | 16·8 | 14·2 | 12·5 |  | | 16·8 | | 14·2 | 12·4 | |  | | 16·8 | | 14·1 | 12·4 |
|  |  | 30% |  | 16·8 | 12·2 | 9·5 |  | | 16·8 | | 12·1 | 9·5 | |  | | 16·8 | | 12·0 | 9·5 |
|  |  | 40% |  | 16·8 | 10·4 | 6·9 |  | | 16·8 | | 10·2 | 6·9 | |  | | 16·8 | | 10·1 | 6·8 |
|  |  | 50% |  | 16·8 | 8·7 | 4·8 |  | | 16·8 | | 8·6 | 4·8 | |  | | 16·8 | | 8·4 | 4·7 |
|  |  | 60% |  | 16·8 | 7·3 | 3·2 |  | | 16·8 | | 7·1 | 3·2 | |  | | 16·8 | | 7·0 | 3·1 |
|  |  | 70% |  | 16·8 | 6·1 | 2·0 |  | | 16·8 | | 5·9 | 2·0 | |  | | 16·8 | | 5·8 | 2·0 |
|  |  | 80% |  | 16·8 | 5·0 | 1·3 |  | | 16·8 | | 4·9 | 1·2 | |  | | 16·8 | | 4·7 | 1·2 |
|  |  | 90% |  | 16·8 | 4·2 | 0·8 |  | | 16·8 | | 4·0 | 0·7 | |  | | 16·8 | | 3·9 | 0·7 |
| 10 Year | | 10% |  | 16·9 | 15·6 | 14·0 |  | | 16·9 | | 15·5 | 14·0 | |  | | 16·9 | | 15·5 | 14·0 |
|  |  | 20% |  | 16·8 | 12·6 | 9·7 |  | | 16·8 | | 12·5 | 9·6 | |  | | 16·8 | | 12·4 | 9·6 |
|  |  | 30% |  | 16·8 | 10·1 | 6·0 |  | | 16·8 | | 9·9 | 6·0 | |  | | 16·8 | | 9·8 | 5·9 |
|  |  | 40% |  | 16·8 | 7·9 | 3·4 |  | | 16·8 | | 7·7 | 3·3 | |  | | 16·8 | | 7·6 | 3·3 |
|  |  | 50% |  | 16·8 | 6·1 | 1·8 |  | | 16·8 | | 5·9 | 1·7 | |  | | 16·8 | | 5·8 | 1·7 |
|  |  | 60% |  | 16·8 | 4·7 | 0·9 |  | | 16·8 | | 4·5 | 0·8 | |  | | 16·8 | | 4·4 | 0·8 |
|  |  | 70% |  | 16·8 | 3·6 | 0·4 |  | | 16·8 | | 3·4 | 0·4 | |  | | 16·8 | | 3·3 | 0·4 |
|  |  | 80% |  | 16·8 | 2·7 | 0·2 |  | | 16·8 | | 2·6 | 0·2 | |  | | 16·8 | | 2·5 | 0·2 |
|  |  | 90% |  | 16·8 | 2·1 | 0·1 |  | | 16·8 | | 2·0 | 0·1 | |  | | 16·8 | | 1·9 | 0·1 |
| 20 Years | | 10% |  | 16·9 | 14·9 | 12·6 |  | | 16·9 | | 14·8 | 12·6 | |  | | 16·9 | | 14·7 | 12·6 |
|  |  | 20% |  | 16·8 | 11·3 | 7·3 |  | | 16·8 | | 11·2 | 7·2 | |  | | 16·8 | | 11·1 | 7·2 |
|  |  | 30% |  | 16·8 | 8·5 | 3·6 |  | | 16·8 | | 8·3 | 3·5 | |  | | 16·8 | | 8·1 | 3·5 |
|  |  | 40% |  | 16·8 | 6·2 | 1·5 |  | | 16·8 | | 6·0 | 1·5 | |  | | 16·8 | | 5·9 | 1·4 |
|  |  | 50% |  | 16·8 | 4·4 | 0·6 |  | | 16·8 | | 4·3 | 0·6 | |  | | 16·8 | | 4·1 | 0·5 |
|  |  | 60% |  | 16·8 | 3·2 | 0·2 |  | | 16·8 | | 3·0 | 0·2 | |  | | 16·8 | | 2·9 | 0·2 |
|  |  | 70% |  | 16·8 | 2·3 | 0·1 |  | | 16·8 | | 2·2 | 0·1 | |  | | 16·8 | | 2·1 | 0·1 |
|  |  | 80% |  | 16·8 | 1·7 | 0·0 |  | | 16·8 | | 1·6 | 0·0 | |  | | 16·8 | | 1·5 | 0·0 |
|  |  | 90% |  | 16·8 | 1·2 | 0·0 |  | | 16·8 | | 1·1 | 0·0 | |  | | 16·8 | | 1·1 | 0·0 |

^1^ IR estimated as # of incident infections per 1,000 person-years (PY)

^2^ IR estimates are for the total population (males and females)

^3^ Under no vaccination, IRs in 2025, 2050, and 2075 were 16·9, 18·5, and 18·7/1,000 PY, respectively

^4^ Scenario D: Scenario B @ 50% coverage + catch-up campaign among unvaccinated 15-24-year-old females in 2035 at 5/10/15% coverage

Abbreviations: CT, *Chlamydia trachomatis*, IR, incidence rate; PY, person-years

| **Table S21.** Scenario Analysis – Assessment of sex-specific screening rates on differences in reduction of incidence rates by 2050 and 2075 comparing Scenario A^1^ or Scenario B^2^ vs. Referent Scenario (No Vaccination) | | | | | | | |  |
| --- | --- | --- | --- | --- | --- | --- | --- | --- |
| **Scenario A** | | | | | | | |  |
| **Age- & Sex-specific Screening Rates** | | | | **Age-specific Screening Rates Only** | | | |  |
| **Year** | **Sex** | **Median Proportional Reduction in Incidence Rate** | **Relative Difference (Males vs. Females) ^3^** | **Year** | **Sex** | **Median Proportional Reduction in Incidence Rate** | **Relative Difference (Males vs. Females) ^3^** | |
| 2050 | Males | 58·3% | 0·836 | 2050 | Males | 73·6% | 0·970 | |
|  | Females | 50·1% |  |  | Females | 72·8% |  |  |
| 2075 | Males | 80·2% | 0·839 | 2075 | Males | 97·0% | 0·983 | |
|  | Females | 76·4% |  |  | Females | 97·0% |  |  |
| **Scenario B** | | | | | | | |  |
| **Age- & Sex-specific Screening Rates** | | | | **Age-specific Screening Rates Only** | | | |  |
| **Year** | **Sex** | **Median Proportional Reduction in Incidence Rate** | **Relative Difference (Males vs. Females) ^3^** | **Year** | **Sex** | **Median Proportional Reduction in Incidence Rate** | **Relative Difference (Males vs. Females) ^3^** | |
| 2050 | Males | 81·7% | 0·813 | 2050 | Males | 91·2% | 0·958 | |
|  | Females | 77·5% |  |  | Females | 90·9% |  |  |
| 2075 | Males | 98·0% | 0·800 | 2075 | Males | 99·8% | 0·980 | |
|  | Females | 97·5% |  |  | Females | 99·8% |  |  |

^1^ Scenario A: Vaccinating only 15-year-old females entering the model between 2025-2075.

^2^ Scenario B: Vaccinating 15-year-old females and males entering the model between 2025-2075.

^3^ Relative difference in proportional incidence rate reduction was calculated as (100 – male reduction) / (100 – female reduction), representing the ratio of residual incidence under vaccination scenarios A or B compared to no vaccination. Values closer to 1 indicate smaller sex differences in vaccine impact. For example, removing sex-specific screening in Scenario A increased this ratio from 0·836 to 0·970 in 2050, suggesting that most of the male-female difference was due to screening differences by sex.

| **Table S22.** Infections prevented per 1,000 doses of vaccine given, by vaccine efficacy and duration of protection for Scenario Group A ^1^ | | | | | | |
| --- | --- | --- | --- | --- | --- | --- |
| **Duration of Protection** | **VE** | **30% Coverage** |  | **50% Coverage** |  | **70% Coverage** |
|  |  | Median (IQR) ^2^ |  | Median (IQR) |  | Median (IQR) |
| 0·5 Years | 10% | 37·6 (37·2, 37·9) |  | 37·6 (37·2, 37·9) |  | 37·5 (37·1, 37·8) |
|  | 20% | 82·7 (81·7, 83·2) |  | 82·6 (81·7, 83·2) |  | 82·5 (81·5, 83) |
|  | 30% | 127·9 (126·5, 128·9) |  | 127·7 (126·2, 128·7) |  | 127·4 (125·9, 128·4) |
|  | 40% | 173·4 (171·5, 174·7) |  | 172·9 (171, 174·3) |  | 172·3 (170·5, 173·8) |
|  | 50% | 219 (216·7, 220·8) |  | 218·2 (216, 220·1) |  | 217·4 (215·1, 219·3) |
|  | 60% | 264·8 (262, 267·1) |  | 263·7 (260·9, 266) |  | 262·5 (259·7, 264·8) |
|  | 70% | 310·9 (307·5, 313·5) |  | 309·3 (306, 312) |  | 307·6 (304·3, 310·4) |
|  | 80% | 357 (353·2, 360·2) |  | 354·9 (351·1, 358·1) |  | 352·7 (348·8, 356·2) |
|  | 90% | 403·4 (399·1, 407) |  | 400·7 (396·3, 404·5) |  | 397·8 (393·4, 401·9) |
| 1 Year | 10% | 69·9 (69·1, 70·4) |  | 69·8 (69, 70·3) |  | 69·6 (68·9, 70·1) |
|  | 20% | 154·2 (152·5, 155·3) |  | 153·8 (152·1, 154·9) |  | 153·3 (151·6, 154·4) |
|  | 30% | 239 (236·3, 240·8) |  | 238 (235·3, 239·9) |  | 236·9 (234·3, 238·8) |
|  | 40% | 324·3 (320·6, 326·9) |  | 322·4 (319, 325·3) |  | 320·5 (317·3, 323·4) |
|  | 50% | 410 (405·7, 413·5) |  | 407·1 (402·9, 410·8) |  | 404·1 (399·8, 408) |
|  | 60% | 496·1 (490·9, 500·6) |  | 491·9 (486·7, 496·7) |  | 487·2 (482·1, 492·5) |
|  | 70% | 582·9 (576·5, 588·2) |  | 576·8 (570·7, 582·8) |  | 569·9 (563·9, 576·8) |
|  | 80% | 669·9 (662·8, 676·3) |  | 661·4 (654·3, 669) |  | 652·4 (645·5, 660·7) |
|  | 90% | 757.3 (749, 765) |  | 746·1 (738·1, 755·2) |  | 734·4 (726·5, 744) |
| 5 Years | 10% | 244 (241·1, 246·4) |  | 242·5 (239·7, 245·1) |  | 240·9 (238·2, 243·6) |
|  | 20% | 528·3 (522·6, 533·7) |  | 522·4 (517·3, 528·2) |  | 516·2 (511, 522·3) |
|  | 30% | 812·7 (804·6, 821·5) |  | 799·1 (790·5, 809·2) |  | 783·8 (775·7, 795·2) |
|  | 40% | 1097·4 (1085·9, 1110·7) |  | 1070·8 (1059·2, 1086·6) |  | 1039·7 (1027·2, 1054·8) |
|  | 50% | 1380·6 (1366·3, 1399·7) |  | 1334·4 (1319·5, 1353·7) |  | 1280·4 (1264·5, 1300·6) |
|  | 60% | 1661·9 (1644·2, 1686·3) |  | 1589·4 (1568·9, 1612·5) |  | 1500·3 (1481·5, 1528·8) |
|  | 70% | 1939·5 (1918·5, 1967·1) |  | 1829·5 (1807·1, 1860·3) |  | 1699·8 (1677·3, 1735·5) |
|  | 80% | 2213·4 (2186·8, 2244·4) |  | 2055·8 (2030·4, 2094·8) |  | 1875·3 (1850·4, 1919) |
|  | 90% | 2479·7 (2450·9, 2517) |  | 2266·7 (2237·5, 2313·6) |  | 2028 (1999·4, 2078·9) |
| 10 Years | 10% | 357 (353·2, 361·1) |  | 353·8 (350·2, 358·1) |  | 350·6 (347, 355) |
|  | 20% | 761·5 (753·7, 771·6) |  | 748·3 (740·9, 758·8) |  | 733·9 (726·5, 744·4) |
|  | 30% | 1162·8 (1151·7, 1179·7) |  | 1129·4 (1118·1, 1146·6) |  | 1091·1 (1078·7, 1108·6) |
|  | 40% | 1558·2 (1543·9, 1582·6) |  | 1492·2 (1474·7, 1515·9) |  | 1414·6 (1395·7, 1441·2) |
|  | 50% | 1947·3 (1924·7, 1976·7) |  | 1831·1 (1806·7, 1862·8) |  | 1694·1 (1671·2, 1731·7) |
|  | 60% | 2324·3 (2296·1, 2360·5) |  | 2139·2 (2109·8, 2183·2) |  | 1927·7 (1900·3, 1976·1) |
|  | 70% | 2688·4 (2654·6, 2732·3) |  | 2415 (2380·5, 2468·6) |  | 2115·8 (2083·3, 2175·5) |
|  | 80% | 3034·2 (2995·9, 3091) |  | 2654·7 (2617·1, 2720·6) |  | 2268·8 (2229·8, 2337·8) |
|  | 90% | 3362·6 (3318, 3432·1) |  | 2860·2 (2817·5, 2938·1) |  | 2390·1 (2347, 2466·5) |
| 20 Years | 10% | 462 (457·1, 467·8) |  | 456·6 (451·6, 463·1) |  | 450·8 (446, 457·5) |
|  | 20% | 972·6 (963·3, 987·4) |  | 948·5 (939, 963·2) |  | 921·1 (911·3, 936·3) |
|  | 30% | 1473·6 (1458·3, 1496·3) |  | 1411 (1394·7, 1434·3) |  | 1338·7 (1320·8, 1366·4) |
|  | 40% | 1959·5 (1937·1, 1990·6) |  | 1833·2 (1809, 1870·6) |  | 1691·3 (1666·6, 1730·3) |
|  | 50% | 2425·5 (2397, 2467) |  | 2210·2 (2177·2, 2258·6) |  | 1968·2 (1937·5, 2020·6) |
|  | 60% | 2866·4 (2829·3, 2923·4) |  | 2529·6 (2491·9, 2592·6) |  | 2179 (2142·9, 2244) |
|  | 70% | 3281·9 (3237·3, 3351·1) |  | 2795·8 (2751·8, 2872·7) |  | 2340·1 (2295·7, 2413·9) |
|  | 80% | 3662·5 (3610·9, 3745·1) |  | 3013·4 (2964·5, 3103·1) |  | 2461·4 (2411, 2542·9) |
|  | 90% | 4009·5 (3950·3, 4106·1) |  | 3194 (3135·4, 3293·3) |  | 2555 (2499·9, 2642·5) |

^1^ Scenario A: Vaccinating only 15-year-old females entering the model between 2025-2075.

^2^ Refers to median (IQR) CT infections prevented per 1,000 doses of vaccine given comparing Scenario A to the Referent Scenario (no vaccination) from 2025-2075.

Abbreviations: CT, *Chlamydia trachomatis*; VE, vaccine efficacy; IQR, interquartile range

| **Table S23.** Infections prevented per 1,000 doses of vaccine given, by vaccine efficacy and duration of protection for Scenario Group B ^1^ | | | | | | |
| --- | --- | --- | --- | --- | --- | --- |
| **Duration of Protection** | **VE** | **30% Coverage** |  | **50% Coverage** |  | **70% Coverage** |
|  |  | Median (IQR) ^2^ |  | Median (IQR) |  | Median (IQR) |
| 0·5 Years | 10% | 37 (36·6, 37·3) |  | 37 (36·6, 37·3) |  | 36·9 (36·5, 37·2) |
|  | 20% | 80·3 (79·4, 80·9) |  | 80·1 (79·2, 80·6) |  | 79·8 (79, 80·4) |
|  | 30% | 123·6 (122·3, 124·5) |  | 123 (121·7, 124) |  | 122·4 (121, 123·4) |
|  | 40% | 166·9 (165, 168·2) |  | 165·8 (164, 167·2) |  | 164·6 (162·9, 166·1) |
|  | 50% | 210·1 (207·8, 211·9) |  | 208·3 (206·2, 210·2) |  | 206·4 (204·5, 208·3) |
|  | 60% | 253·3 (250·7, 255·5) |  | 250·7 (248·2, 253) |  | 247·9 (245·5, 250·3) |
|  | 70% | 296·5 (293·5, 299·1) |  | 292·8 (289·9, 295·5) |  | 288·9 (286, 291·9) |
|  | 80% | 339·6 (336·2, 342·6) |  | 334·7 (331·3, 337·9) |  | 329·5 (325·8, 333·1) |
|  | 90% | 382·7 (378·8, 386·1) |  | 376·3 (372·4, 380·1) |  | 369·3 (365·1, 373·7) |
| 1 Year | 10% | 68·8 (68·1, 69·3) |  | 68·7 (67·9, 69·2) |  | 68·4 (67·7, 68·9) |
|  | 20% | 149·8 (148·2, 150·9) |  | 149 (147·4, 150·1) |  | 148 (146·4, 149·2) |
|  | 30% | 230·8 (228·1, 232·5) |  | 228·5 (226, 230·4) |  | 226 (223·8, 228·2) |
|  | 40% | 311·3 (307·9, 313·9) |  | 307 (304·1, 310) |  | 302·5 (299·7, 305·9) |
|  | 50% | 391·6 (387·5, 395·1) |  | 384·6 (380·9, 388·8) |  | 377·3 (373·3, 381·9) |
|  | 60% | 471·5 (466·8, 476) |  | 461·2 (456·4, 466·5) |  | 449·9 (444·6, 455·8) |
|  | 70% | 550·9 (545·4, 556·6) |  | 536·2 (530·4, 543) |  | 520·3 (514, 527·2) |
|  | 80% | 630·1 (623·6, 636·9) |  | 610 (602·9, 618) |  | 587·5 (580·3, 596) |
|  | 90% | 708·6 (700·9, 716·8) |  | 682·2 (673·9, 691·2) |  | 651·5 (643·3, 661·7) |
| 5 Years | 10% | 238·7 (236·1, 241·3) |  | 235·9 (233·5, 238·6) |  | 232·8 (230·7, 235·7) |
|  | 20% | 507·7 (502·7, 513·6) |  | 494·6 (489·6, 501·1) |  | 479·2 (473·8, 486·4) |
|  | 30% | 769·2 (761·3, 779·2) |  | 733·8 (725·1, 744·9) |  | 693·9 (684·8, 705·1) |
|  | 40% | 1020 (1008·6, 1035·3) |  | 949·7 (937·3, 965) |  | 869·1 (857·6, 887·7) |
|  | 50% | 1258·7 (1242·1, 1277) |  | 1136·8 (1122·1, 1159·3) |  | 1005·4 (991·5, 1030·7) |
|  | 60% | 1482·1 (1462·9, 1505·3) |  | 1294·9 (1277, 1324·5) |  | 1108·4 (1090·3, 1140) |
|  | 70% | 1687·5 (1666·1, 1718·3) |  | 1424·5 (1404·5, 1460·5) |  | 1185 (1164·1, 1221·7) |
|  | 80% | 1876·4 (1852·5, 1913·9) |  | 1530·8 (1506·5, 1573·4) |  | 1242·8 (1220·3, 1283·3) |
|  | 90% | 2046·9 (2018·6, 2091·4) |  | 1616·4 (1588·4, 1665) |  | 1287·2 (1262·2, 1330·7) |
| 10 Years | 10% | 347·3 (343·9, 352) |  | 341·1 (337·7, 345·5) |  | 333·8 (330·5, 338·6) |
|  | 20% | 723·3 (716·6, 733·6) |  | 690·6 (682·2, 701·6) |  | 653·4 (644·6, 665·5) |
|  | 30% | 1076·9 (1063·6, 1093·5) |  | 990·9 (977·6, 1009·8) |  | 896·6 (883·5, 917·9) |
|  | 40% | 1401·5 (1383·3, 1424·4) |  | 1233·5 (1215·6, 1261·8) |  | 1061·9 (1045·3, 1092·2) |
|  | 50% | 1692·5 (1670·5, 1726·4) |  | 1416·8 (1395·8, 1455·3) |  | 1173·4 (1151·4, 1210·2) |
|  | 60% | 1947 (1920·4, 1989·9) |  | 1554·8 (1528·7, 1601·8) |  | 1248·1 (1222·5, 1289·4) |
|  | 70% | 2164·9 (2133·4, 2217·8) |  | 1657·5 (1626·7, 1710·1) |  | 1300·5 (1272·5, 1345·6) |
|  | 80% | 2346·7 (2312, 2409·9) |  | 1734·6 (1700·1, 1791·7) |  | 1339·1 (1310·1, 1386·9) |
|  | 90% | 2499·3 (2459·7, 2571·8) |  | 1794 (1756·1, 1855·3) |  | 1369 (1339·2, 1419·4) |
| 20 Years | 10% | 447·1 (442·3, 453·4) |  | 435 (430·2, 441·7) |  | 421·6 (416·6, 428·6) |
|  | 20% | 911·7 (901·4, 925·5) |  | 850·6 (839·1, 867) |  | 784·4 (773·3, 802·3) |
|  | 30% | 1330·5 (1313·1, 1354·6) |  | 1177 (1159·6, 1203·9) |  | 1020·6 (1003·7, 1048·9) |
|  | 40% | 1696·5 (1672·9, 1732·7) |  | 1410 (1387·2, 1448·8) |  | 1162·5 (1140, 1199·2) |
|  | 50% | 2004 (1974·1, 2051·5) |  | 1570·8 (1542·7, 1619·7) |  | 1249·9 (1222·8, 1292) |
|  | 60% | 2251·9 (2217, 2311·1) |  | 1683·2 (1649·1, 1737·5) |  | 1307·1 (1278·3, 1353·3) |
|  | 70% | 2448·8 (2409·6, 2519·7) |  | 1762·8 (1724·6, 1822·7) |  | 1347·9 (1318·2, 1396·7) |
|  | 80% | 2608·2 (2561·9, 2689·3) |  | 1821·7 (1781·6, 1885·8) |  | 1378·7 (1348·4, 1429·9) |
|  | 90% | 2735·4 (2682·5, 2822·3) |  | 1867·3 (1826·3, 1934·5) |  | 1402·9 (1372·3, 1455·6) |

^1^ Scenario B: Vaccinating 15-year-old females and males entering the model between 2025-2075.

^2^ Refers to median (IQR) CT infections prevented per 1,000 doses of vaccine given comparing Scenario B to the referent scenario (no vaccination) from 2025-2075.

Abbreviations: CT, *Chlamydia trachomatis*; VE, vaccine efficacy; IQR, interquartile range

| **Table S24.** Infections prevented per 1,000 doses of vaccine given, by vaccine efficacy and duration of protection for Scenario Group C ^1^ | | | | | | |
| --- | --- | --- | --- | --- | --- | --- |
| **Duration of Protection** | **VE** | **5% Catch-up Coverage** |  | **10% Catch-up Coverage** |  | **15% Catch-up Coverage** |
|  |  | Median (IQR) ^2^ |  | Median (IQR) |  | Median (IQR) |
| 0·5 Years | 10% | 37·9 (37·5, 38·2) |  | 38·2 (37·8, 38·5) |  | 38·5 (38·1, 38·8) |
|  | 20% | 83·1 (82·1, 83·7) |  | 83·6 (82·6, 84·1) |  | 84·1 (83, 84·6) |
|  | 30% | 128·4 (126·9, 129·3) |  | 129 (127·6, 130) |  | 129·7 (128·2, 130·6) |
|  | 40% | 173·8 (171·8, 175·2) |  | 174·6 (172·6, 176) |  | 175·4 (173·4, 176·8) |
|  | 50% | 219·3 (217, 221·1) |  | 220·4 (217·9, 222·1) |  | 221·3 (218·8, 223·1) |
|  | 60% | 265 (262·2, 267·2) |  | 266·2 (263·4, 268·4) |  | 267·4 (264·5, 269·5) |
|  | 70% | 310·8 (307·4, 313·5) |  | 312·2 (308·8, 314·9) |  | 313·5 (310·1, 316·1) |
|  | 80% | 356·6 (352·9, 359·8) |  | 358·2 (354·4, 361·4) |  | 359·8 (355·9, 362·9) |
|  | 90% | 402·6 (398·2, 406·3) |  | 404·4 (400·1, 408) |  | 406·1 (401·8, 409·8) |
| 1 Year | 10% | 70·4 (69·6, 71) |  | 71 (70·2, 71·6) |  | 71·6 (70·8, 72·1) |
|  | 20% | 154·8 (153·1, 155·9) |  | 155·8 (154·1, 156·9) |  | 156·7 (154·9, 157·8) |
|  | 30% | 239·5 (236·8, 241·3) |  | 240·8 (238·1, 242·6) |  | 242·1 (239·4, 243·8) |
|  | 40% | 324·3 (320·8, 327) |  | 326·1 (322·5, 328·7) |  | 327·7 (324·1, 330·4) |
|  | 50% | 409·3 (405·1, 413) |  | 411·5 (407·2, 415·1) |  | 413·5 (409·2, 417·1) |
|  | 60% | 494·6 (489·3, 499·3) |  | 497·2 (491·8, 501·7) |  | 499·6 (494·2, 504) |
|  | 70% | 580 (573·9, 585·9) |  | 583 (576·7, 588·8) |  | 585·8 (579·4, 591·5) |
|  | 80% | 665·1 (658, 672·5) |  | 668·6 (661·4, 675·9) |  | 671·8 (664·7, 679) |
|  | 90% | 750 (742·1, 759·1) |  | 753·9 (745·7, 762·9) |  | 757·6 (749·5, 766·5) |
| 5 Years | 10% | 245 (242·1, 247·5) |  | 247·3 (244·3, 249·7) |  | 249·4 (246·4, 251·7) |
|  | 20% | 526·6 (521·4, 532·4) |  | 530·5 (525·3, 536·4) |  | 534·1 (528·8, 540·1) |
|  | 30% | 805·2 (796·4, 815·2) |  | 810·7 (802, 820·8) |  | 815·8 (807·1, 825·9) |
|  | 40% | 1078·5 (1067, 1094·4) |  | 1085·6 (1074·2, 1101·6) |  | 1092·1 (1080·7, 1108·3) |
|  | 50% | 1343·6 (1328·7, 1363·2) |  | 1352 (1337·1, 1371·9) |  | 1359·9 (1344·9, 1379·9) |
|  | 60% | 1600 (1579·3, 1623·1) |  | 1609·7 (1588·8, 1633) |  | 1618·6 (1597·5, 1642) |
|  | 70% | 1840·8 (1818·3, 1871·9) |  | 1851·2 (1828·4, 1882·5) |  | 1860·7 (1837·7, 1892·2) |
|  | 80% | 2067·4 (2041·9, 2107) |  | 2077·8 (2052·2, 2118·1) |  | 2087·3 (2061·4, 2128) |
|  | 90% | 2278 (2248·5, 2325·6) |  | 2288·2 (2258·3, 2336·2) |  | 2297·3 (2267, 2345·6) |
| 10 Years | 10% | 357·5 (353·8, 361·7) |  | 360·8 (357·1, 365·1) |  | 363·9 (360·1, 368·2) |
|  | 20% | 754·6 (747·3, 765·3) |  | 760·4 (753·1, 771·3) |  | 765·7 (758·4, 776·8) |
|  | 30% | 1138·3 (1127·1, 1155·8) |  | 1146·5 (1135·4, 1164·2) |  | 1154 (1143, 1171·9) |
|  | 40% | 1503·2 (1485·6, 1527·3) |  | 1513·4 (1495·6, 1537·7) |  | 1522·7 (1504·7, 1547·2) |
|  | 50% | 1843·4 (1818·8, 1875·8) |  | 1854·4 (1829·7, 1887·5) |  | 1864·5 (1839·5, 1898·2) |
|  | 60% | 2151·9 (2122·1, 2196·4) |  | 2163·2 (2133·1, 2208·1) |  | 2173·1 (2142·8, 2218·6) |
|  | 70% | 2426·5 (2391·6, 2480·9) |  | 2436·2 (2401, 2491·6) |  | 2444·5 (2409, 2500·8) |
|  | 80% | 2663·9 (2625·7, 2731) |  | 2671·1 (2632·4, 2739·4) |  | 2676·6 (2637·5, 2746·2) |
|  | 90% | 2866 (2822·8, 2945·4) |  | 2870 (2826·3, 2950·6) |  | 2872·4 (2828·2, 2954) |
| 20 Years | 10% | 461·3 (456·4, 468) |  | 465·8 (460·8, 472·5) |  | 469·9 (464·9, 476·6) |
|  | 20% | 957 (947·3, 971·9) |  | 964·8 (955, 979·9) |  | 972 (962, 987·2) |
|  | 30% | 1422·4 (1405·9, 1446) |  | 1432·7 (1416·2, 1456·8) |  | 1442·3 (1425·6, 1466·6) |
|  | 40% | 1846·3 (1821·8, 1884·5) |  | 1858·1 (1833·3, 1897) |  | 1868·7 (1843·7, 1908·1) |
|  | 50% | 2223·1 (2190, 2272·2) |  | 2234·5 (2201·3, 2284·2) |  | 2244·4 (2211·1, 2294·8) |
|  | 60% | 2540·3 (2502·4, 2604·5) |  | 2549·2 (2511·4, 2614·4) |  | 2556·5 (2518·8, 2622·4) |
|  | 70% | 2802·7 (2758·4, 2881·1) |  | 2807·7 (2763·1, 2887·2) |  | 2810·9 (2766, 2891·2) |
|  | 80% | 3016·2 (2966·4, 3106·9) |  | 3016·9 (2966·2, 3108·8) |  | 3015·8 (2963·9, 3108·7) |
|  | 90% | 3191·3 (3131·9, 3291·2) |  | 3186·4 (3126·4, 3286·9) |  | 3179·8 (3119·3, 3281·1) |

^1^ Scenario C: Scenario A at 50% coverage + catch-up campaign among unvaccinated 15-24-year-old females in 2035

^2^ Refers to median (IQR) CT infections prevented per 1,000 doses of vaccine given comparing Scenario C to the referent scenario (no vaccination) from 2025-2075.

Abbreviations: CT, *Chlamydia trachomatis*; VE, vaccine efficacy; IQR, interquartile range

| **Table S25.** Infections prevented per 1,000 doses of vaccine given, by vaccine efficacy and duration of protection for Scenario Group D ^1^ | | | | | | |
| --- | --- | --- | --- | --- | --- | --- |
| **Duration of Protection** | **VE** | **5% Catch-up Coverage** |  | **10% Catch-up Coverage** |  | **15% Catch-up Coverage** |
|  |  | Median (IQR) ^2^ |  | Median (IQR) |  | Median (IQR) |
| 0·5 Years | 10% | 37·2 (36·8, 37·4) |  | 37·3 (36·9, 37·6) |  | 37·5 (37·1, 37·7) |
|  | 20% | 80·4 (79·5, 80·9) |  | 80·6 (79·7, 81·2) |  | 80·9 (80, 81·4) |
|  | 30% | 123·4 (122·1, 124·4) |  | 123·8 (122·4, 124·7) |  | 124·2 (122·8, 125·1) |
|  | 40% | 166·3 (164·5, 167·7) |  | 166·8 (164·9, 168·2) |  | 167·3 (165·4, 168·7) |
|  | 50% | 209 (206·8, 210·8) |  | 209·6 (207·3, 211·4) |  | 210·1 (207·9, 211·9) |
|  | 60% | 251·4 (248·9, 253·7) |  | 252·1 (249·6, 254·4) |  | 252·8 (250·3, 255·1) |
|  | 70% | 293·7 (290·8, 296·4) |  | 294·5 (291·6, 297·2) |  | 295·3 (292·4, 298) |
|  | 80% | 335·6 (332·2, 338·9) |  | 336·6 (333·1, 339·8) |  | 337·5 (334, 340·7) |
|  | 90% | 377·3 (373·5, 381·2) |  | 378·4 (374·5, 382·3) |  | 379·4 (375·5, 383·3) |
| 1 Year | 10% | 69 (68·3, 69·5) |  | 69·3 (68·6, 69·8) |  | 69·6 (68·9, 70·1) |
|  | 20% | 149·5 (147·9, 150·7) |  | 150·1 (148·4, 151·2) |  | 150·6 (148·9, 151·7) |
|  | 30% | 229·3 (226·8, 231·2) |  | 230·1 (227·5, 232) |  | 230·8 (228·3, 232·7) |
|  | 40% | 308·1 (305·1, 311) |  | 309·1 (306, 312) |  | 310 (306·9, 312·9) |
|  | 50% | 385·9 (382·1, 390) |  | 387·1 (383·3, 391·2) |  | 388·2 (384·4, 392·3) |
|  | 60% | 462·7 (457·9, 468) |  | 464·1 (459·3, 469·4) |  | 465·4 (460·7, 470·8) |
|  | 70% | 537·9 (532·1, 544·7) |  | 539·5 (533·8, 546·3) |  | 541 (535·4, 547·9) |
|  | 80% | 611·9 (604·8, 619·9) |  | 613·7 (606·5, 621·8) |  | 615·4 (608·2, 623·5) |
|  | 90% | 684·3 (676, 693·3) |  | 686·3 (678, 695·3) |  | 688·2 (679·9, 697·3) |
| 5 Years | 10% | 237·1 (234·7, 239·9) |  | 238·3 (235·9, 241·1) |  | 239·4 (236·9, 242·2) |
|  | 20% | 496·7 (491·7, 503·2) |  | 498·7 (493·6, 505·2) |  | 500·5 (495·4, 507·1) |
|  | 30% | 736·5 (727·9, 747·7) |  | 739·1 (730·5, 750·4) |  | 741·6 (733, 752·8) |
|  | 40% | 952·9 (940·4, 968·3) |  | 955·9 (943·3, 971·4) |  | 958·6 (946, 974·3) |
|  | 50% | 1139·9 (1125·2, 1162·6) |  | 1142·7 (1128, 1165·6) |  | 1145·3 (1130·5, 1168·4) |
|  | 60% | 1297·4 (1279·5, 1327·3) |  | 1299·7 (1281·8, 1329·9) |  | 1301·7 (1283·9, 1332·1) |
|  | 70% | 1426 (1405·9, 1462·3) |  | 1427·3 (1407, 1463·9) |  | 1428·4 (1407·9, 1465·2) |
|  | 80% | 1531·2 (1506·6, 1574·3) |  | 1531·3 (1506·5, 1574·8) |  | 1531·2 (1506·2, 1575·1) |
|  | 90% | 1615·5 (1587·1, 1664·3) |  | 1614·3 (1585·7, 1663·4) |  | 1612·8 (1584·3, 1662·3) |
| 10 Years | 10% | 342·9 (339·4, 347·4) |  | 344·6 (341·1, 349·2) |  | 346·2 (342·6, 350·8) |
|  | 20% | 693·5 (685·1, 704·6) |  | 696·3 (687·9, 707·4) |  | 698·9 (690·4, 710) |
|  | 30% | 994·3 (981, 1013·5) |  | 997·4 (984·1, 1016·9) |  | 1000·4 (987, 1020) |
|  | 40% | 1236·4 (1218·5, 1265) |  | 1239 (1221, 1267·9) |  | 1241·4 (1223·3, 1270·6) |
|  | 50% | 1418·4 (1397·2, 1457·2) |  | 1419·7 (1398·4, 1458·8) |  | 1420·7 (1399·3, 1460·1) |
|  | 60% | 1554·8 (1528·4, 1602·1) |  | 1554·5 (1527·8, 1602) |  | 1554 (1527, 1601·6) |
|  | 70% | 1655·6 (1624·8, 1708·3) |  | 1653·4 (1622·7, 1706·3) |  | 1651·1 (1620·3, 1704·1) |
|  | 80% | 1730·8 (1696·1, 1788·2) |  | 1727 (1691·9, 1784·5) |  | 1723 (1687·6, 1780·7) |
|  | 90% | 1788·6 (1750·6, 1850) |  | 1783·1 (1745·1, 1844·6) |  | 1777·6 (1739·6, 1839·2) |
| 20 Years | 10% | 437·3 (432·5, 444) |  | 439·5 (434·7, 446·3) |  | 441·6 (436·8, 448·3) |
|  | 20% | 854·1 (842·6, 870·6) |  | 857·4 (845·8, 874) |  | 860·4 (848·9, 877·1) |
|  | 30% | 1180·3 (1162·8, 1207·4) |  | 1183·2 (1165·7, 1210·7) |  | 1185·9 (1168·3, 1213·6) |
|  | 40% | 1411·7 (1388·8, 1450·7) |  | 1413 (1390, 1452·4) |  | 1414·1 (1391, 1453·7) |
|  | 50% | 1570·4 (1541·9, 1619·3) |  | 1569·7 (1540·8, 1618·7) |  | 1568·8 (1539·6, 1617·9) |
|  | 60% | 1680·5 (1646·1, 1735) |  | 1677·6 (1642·9, 1732·3) |  | 1674·6 (1639·7, 1729·5) |
|  | 70% | 1758 (1719·7, 1818·1) |  | 1753·1 (1714·7, 1813·4) |  | 1748·2 (1709·8, 1808·6) |
|  | 80% | 1815·1 (1775·1, 1879·3) |  | 1808·6 (1768·8, 1872·8) |  | 1802·2 (1762·5, 1866·3) |
|  | 90% | 1859·3 (1818·4, 1926·4) |  | 1851·4 (1810·8, 1918·4) |  | 1843·7 (1803·3, 1910·6) |

^1^ Scenario D: Scenario B at 50% coverage + catch-up campaign among unvaccinated 15-24-year-old females in 2035

^2^ Refers to median (IQR) CT infections prevented per 1,000 doses of vaccine given comparing Scenario D to the referent scenario (no vaccination) from 2025-2075.

Abbreviations: CT, *Chlamydia trachomatis*; VE, vaccine efficacy; IQR, interquartile range

| **Table S26.** Absolute^1^ and relative reduction^2^ in sequelae between 2025-2075, comparing vaccination scenarios to no vaccination, under base-case assumptions^3,*^ | | |
| --- | --- | --- |
| Scenario A ^4^ | | |
| Sequelae | Absolute Reduction | Relative Reduction (%) |
| Epididymitis | 430,054 | 35·5 |
| Pelvic Inflammatory Disease | 3,456,280 | 37·1 |
| Ectopic Pregnancy | 262,677 | 37·1 |
| Tubal Factor Infertility | 345,628 | 37·1 |
| Chronic Pelvic Pain | 622,130 | 37·1 |
| Scenario B ^5^ | | |
| Sequelae | Absolute Reduction | Relative Reduction (%) |
| Epididymitis | 618,168 | 51·1 |
| Pelvic Inflammatory Disease | 4,778,155 | 51·4 |
| Ectopic Pregnancy | 363,140 | 51·4 |
| Tubal Factor Infertility | 477,816 | 51·4 |
| Chronic Pelvic Pain | 860,068 | 51·4 |
| Scenario C ^6^ | | |
| Sequelae | Absolute Reduction | Relative Reduction (%) |
| Epididymitis | 450,636 | 37·2 |
| Pelvic Inflammatory Disease | 3,615,989 | 38·9 |
| Ectopic Pregnancy | 274,815 | 38·9 |
| Tubal Factor Infertility | 361,599 | 38·9 |
| Chronic Pelvic Pain | 650,878 | 38·9 |
| Scenario D ^7^ | | |
| Sequelae | Absolute Reduction | Relative Reduction (%) |
| Epididymitis | 627,586 | 51·8 |
| Pelvic Inflammatory Disease | 4,856,718 | 52·2 |
| Ectopic Pregnancy | 369,111 | 52·2 |
| Tubal Factor Infertility | 485,672 | 52·2 |
| Chronic Pelvic Pain | 874,209 | 52·2 |

^1^ Refers to difference in total CT infections comparing vaccination scenarios to the referent scenario (no vaccination) from 2025-2075.

^2^ Refers to percent reduction in total CT infections comparing vaccination scenarios to the referent scenario (no vaccination) from 2025-2075.

^3^ Base-case assumes 10 years of vaccine-conferred immunity, 70% vaccine efficacy, and 50% routine coverage attained by 2035, with 10% coverage for a catch-up campaign in 2035.

^4^ Scenario A: Vaccinating only 15-year-old females entering the model between 2025-2075.

^5^ Scenario B: Vaccinating 15-year-old females and males entering the model between 2025-2075.

^6^ Scenario C: Scenario A @ 50% coverage + catch-up campaign among unvaccinated 15-24-year-old females in 2035.

^7^ Scenario D: Scenario B @ 50% coverage + catch-up campaign among unvaccinated 15-24-year-old females in 2035.

* Assumes that pelvic inflammatory disease develops into ectopic pregnancy, tubal factor infertility, and chronic pain among 7·6%, 10%, and 18% of individuals (Kumar et al. 2021; PMID: 33492090).

Abbreviations: Vx, vaccination

| **Table S27.** Sequential cost-effectiveness analysis for female-only, sex-neutral, and catch-up vaccination strategies, under base-case assumptions ^1^ with 30%, 50%, or 90% VE (2025 - 2050) | | | | | | | | |
| --- | --- | --- | --- | --- | --- | --- | --- | --- |
| **30% VE** | | | | | | | | |
| **A. Female-only Vaccination** | | | | | | | | |
| Step | Intervention | Comparator | Total Cost (Million USD) ^2,3^ | Total QALY Decrements ^2^ | Δ Cost  (Million USD) | Δ QALYs Gained | **ICER ($/QALY)** |  |
| 1 | No Vaccination | -- | 147,921·94 | 1,257,109 | -- | -- | -- |  |
| 2 | Scenario A | No Vaccination | 148,468·41 | 1,149,221 | 546·47 | 107,888 | **5,065**·**15** |  |
| 3 | Scenario C | Scenario A | 148,453·03 | 1,136,710 | -15·38 | 12,511 | **Cost Saving** |  |
| **B. Sex-neutral Vaccination** | | | | | | | |  |
| Step | Intervention | Comparator | Total Cost (Million USD) ^2,3^ | Total QALY Decrements ^2^ | Δ Cost  (Million USD) | Δ QALYs Gained | **ICER ($/QALY)** |  |
| 1 | No Vaccination | -- | 147,921·94 | 1,257,109 | -- | -- | -- |  |
| 2 | Scenario A | No Vaccination | 148,468·41 | 1,149,221 | 546·47 | 107,888 | **5,065**·**15** |  |
| 3 | Scenario B | Scenario A | 149,165·68 | 1,061,454 | 697·27 | 87,767 | **7,944**·**56** |  |
| 4 | Scenario D | Scenario B | 149,182·04 | 1,050,750 | 16·36 | 10,704 | **1,528**·**56** |  |
| **50% VE** | | | | | | | |  |
| **A. Female-only Vaccination** | | | | | | | |  |
| Step | Intervention | Comparator | Total Cost (Million USD) ^2,3^ | Total QALY Decrements ^2^ | Δ Cost  (Million USD) | Δ QALYs Gained | **ICER ($/QALY)** |  |
| 1 | No Vaccination | -- | 147,921·94 | 1,257,109 | -- | -- | -- |  |
| 2 | Scenario A | No Vaccination | 147,159·66 | 1,072,825 | -762·28 | 184,284 | **Cost Saving** |  |
| 3 | Scenario C | Scenario A | 147,032·98 | 1,053,829 | -126·69 | 18,996 | **Cost Saving** |  |
| **B. Sex-neutral Vaccination** | | | | | | | |  |
| Step | Intervention | Comparator | Total Cost (Million USD) ^2,3^ | Total QALY Decrements ^2^ | Δ Cost  (Million USD) | Δ QALYs Gained | **ICER ($/QALY)** |  |
| 1 | No Vaccination | -- | 147,921·94 | 1,257,109 | -- | -- | -- |  |
| 2 | Scenario A | No Vaccination | 147,159·66 | 1,072,825 | -762·28 | 184,284 | **Cost Saving** |  |
| 3 | Scenario B | Scenario A | 147,176·56 | 947,167 | 16·89 | 125,658 | **134**·**45** |  |
| 4 | Scenario D | Scenario B | 147,136·15 | 933,146 | -40·41 | 14,021 | **Cost Saving** |  |
| **90% VE** | | | | | | | |  |
| **A. Female-only Vaccination** | | | | | | | |  |
| Step | Intervention | Comparator | Total Cost (Million USD) ^2,3^ | Total QALY Decrements ^2^ | Δ Cost  (Million USD) | Δ QALYs Gained | **ICER ($/QALY)** |  |
| 1 | No Vaccination | -- | 147,921·94 | 1,257,109 | -- | -- | -- |  |
| 2 | Scenario A | No Vaccination | 144,846·78 | 938,160 | -3,075·16 | 318,949 | **Cost Saving** |  |
| 3 | Scenario C | Scenario A | 144,584·79 | 911,378 | -261·99 | 26,782 | **Cost Saving** |  |
| **B. Sex-neutral Vaccination** | | | | | | | |  |
| Step | Intervention | Comparator | Total Cost (Million USD) ^2,3^ | Total QALY Decrements ^2^ | Δ Cost  (Million USD) | Δ QALYs Gained | **ICER ($/QALY)** |  |
| 1 | No Vaccination | -- | 147,921·94 | 1,257,109 | -- | -- | -- |  |
| 2 | Scenario A | No Vaccination | 144,846·78 | 938,160 | -3,075·16 | 318,949 | **Cost Saving** |  |
| 3 | Scenario B | Scenario A | 144,355·73 | 785,219 | -491·05 | 152,941 | **Cost Saving** |  |
| 4 | Scenario D | Scenario B | 144,311·30 | 770,897 | -44·43 | 14,322 | **Cost Saving** |  |
| ^1^ Base-case assumptions include Scenarios A-D: 50% coverage by 2035, 10-year duration of protection, and 70% VE; Scenarios C/D: 10% catch-up campaign coverage in 2035.  ^2^ Costs and QALYs are discounted at 3% annually.  ^3^ Costs are in 2025 USD  Abbreviations: USD, United States Dollar, QALY, quality-adjusted life years; VE, vaccine efficacy | | | | | | | | |

| **Table S28**. Sequential cost-effectiveness analysis for female-only, sex-neutral, and catch-up vaccination strategies, under base-case assumptions ^1^ with 5- or 20-year duration of protection (2025 - 2050) | | | | | | | | |
| --- | --- | --- | --- | --- | --- | --- | --- | --- |
| **5 Years** | | | | | | | | |
| **A. Female-only Vaccination** | | | | | | | | |
| Step | Intervention | Comparator | Total Cost (Million USD) ^2,3^ | Total QALY Decrements ^2^ | Δ Cost  (Million USD) | Δ QALYs Gained | **ICER ($/QALY)** |  |
| 1 | No Vaccination | -- | 147,921·94 | 1,257,109 | -- | -- | -- |  |
| 2 | Scenario A | No Vaccination | 146,883·14 | 1,058,827 | -1,038·80 | 198,282 | **Cost Saving** |  |
| 3 | Scenario C | Scenario A | 146,731·75 | 1,037,991 | -151·39 | 20,836 | **Cost Saving** |  |
| **B. Sex-neutral Vaccination** | | | | | | | |  |
| Step | Intervention | Comparator | Total Cost (Million USD) ^2,3^ | Total QALY Decrements ^2^ | Δ Cost  (Million USD) | Δ QALYs Gained | **ICER ($/QALY)** |  |
| 1 | No Vaccination | -- | 147,921·94 | 1,257,109 | -- | -- | -- |  |
| 2 | Scenario A | No Vaccination | 146,883·14 | 1,058,827 | -1,038·80 | 198,282 | **Cost Saving** |  |
| 3 | Scenario B | Scenario A | 146,796·49 | 927,028 | -86·65 | 131,799 | **Cost Saving** |  |
| 4 | Scenario D | Scenario B | 146,743·37 | 911,761 | -53·12 | 15,267 | **Cost Saving** |  |
| **20 Years** | | | | | | | |  |
| **A. Female-only Vaccination** | | | | | | | |  |
| Step | Intervention | Comparator | Total Cost (Million USD) ^2,3^ | Total QALY Decrements ^2^ | Δ Cost  (Million USD) | Δ QALYs Gained | **ICER ($/QALY)** |  |
| 1 | No Vaccination | -- | 147,921·94 | 1,257,109 | -- | -- | -- |  |
| 2 | Scenario A | No Vaccination | 145,298·09 | 962,984 | -2,623·85 | 294,125 | **Cost Saving** |  |
| 3 | Scenario C | Scenario A | 145,066·22 | 938,269 | -231·87 | 24,715 | **Cost Saving** |  |
| **B. Sex-neutral Vaccination** | | | | | | | |  |
| Step | Intervention | Comparator | Total Cost (Million USD) ^2,3^ | Total QALY Decrements ^2^ | Δ Cost  (Million USD) | Δ QALYs Gained | **ICER ($/QALY)** |  |
| 1 | No Vaccination | -- | 147,921·94 | 1,257,109 | -- | -- | -- |  |
| 2 | Scenario A | No Vaccination | 145,298·09 | 962,984 | -2,623·85 | 294,125 | **Cost Saving** |  |
| 3 | Scenario B | Scenario A | 144,873·26 | 813,863 | -424·83 | 149,121 | **Cost Saving** |  |
| 4 | Scenario D | Scenario B | 144,832·87 | 800,151 | -40·38 | 13,712 | **Cost Saving** |  |
| ^1^ Base-case assumptions include Scenarios A-D: 50% coverage by 2035, 10-year duration of protection, and 70% VE; Scenarios C/D: 10% catch-up campaign coverage in 2035.  ^2^ Costs and QALYs are discounted at 3% annually.  ^3^ Costs are in 2025 USD  Abbreviations: USD, United States Dollar, QALY, quality-adjusted life years; VE, vaccine efficacy | | | | | | | | |

| **Table S29.** Sequential cost-effectiveness analysis for female-only, sex-neutral, and catch-up vaccination strategies, under base-case assumptions ^1^ with 30% or 70% vaccine coverage by 2035 (2025 - 2050) | | | | | | | | |
| --- | --- | --- | --- | --- | --- | --- | --- | --- |
| **30% Coverage by 2035** | | | | | | | | |
| **A. Female-only Vaccination** | | | | | | | | |
| Step | Intervention | Comparator | Total Cost (Million USD) ^2,3^ | Total QALY Decrements ^2^ | Δ Cost  (Million USD) | Δ QALYs Gained | **ICER ($/QALY)** |  |
| 1 | No Vaccination | -- | 147,921·94 | 1,257,109 | -- | -- | -- |  |
| 2 | Scenario A | No Vaccination | 146,545·46 | 1,092,704 | -1,376·48 | 164,405 | **Cost Saving** |  |
| **B. Sex-neutral Vaccination** | | | | | | | |  |
| Step | Intervention | Comparator | Total Cost (Million USD) ^2,3^ | Total QALY Decrements ^2^ | Δ Cost  (Million USD) | Δ QALYs Gained | **ICER ($/QALY)** |  |
| 1 | No Vaccination | -- | 147,921·94 | 1,257,109 | -- | -- | -- |  |
| 2 | Scenario A | No Vaccination | 146,545·46 | 1,092,704 | -1,376·48 | 164,405 | **Cost Saving** |  |
| 3 | Scenario B | Scenario A | 145,864·42 | 978,480 | -681·04 | 114,224 | **Cost Saving** |  |
| **70% Coverage by 2035** | | | | | | | |  |
| **A. Female-only Vaccination** | | | | | | | |  |
| Step | Intervention | Comparator | Total Cost (Million USD) ^2,3^ | Total QALY Decrements ^2^ | Δ Cost  (Million USD) | Δ QALYs Gained | **ICER ($/QALY)** |  |
| 1 | No Vaccination | -- | 147,921·94 | 1,257,109 | -- | -- | -- |  |
| 2 | Scenario A | No Vaccination | 145,584·33 | 925,448 | -2,337·61 | 331,661 | **Cost Saving** |  |
| **B. Sex-neutral Vaccination** | | | | | | | |  |
| Step | Intervention | Comparator | Total Cost (Million USD) ^2,3^ | Total QALY Decrements ^2^ | Δ Cost  (Million USD) | Δ QALYs Gained | **ICER ($/QALY)** |  |
| 1 | No Vaccination | -- | 147,921·94 | 1,257,109 | -- | -- | -- |  |
| 2 | Scenario A | No Vaccination | 145,584·33 | 925,448 | -2,337·61 | 331,661 | **Cost Saving** |  |
| 3 | Scenario B | Scenario A | 145,944·09 | 769,895 | 359·75 | 155,553 | **Cost Saving** |  |
| ^1^ Base-case assumptions include Scenarios A-D: 50% coverage by 2035, 10-year duration of protection, and 70% VE; Scenarios C/D: 10% catch-up campaign coverage in 2035.  ^2^ Costs and QALYs are discounted at 3% annually.  ^3^ Costs are in 2025 USD  Abbreviations: USD, United States Dollar, QALY, quality-adjusted life years; VE, vaccine efficacy | | | | | | | | |

| **Table S30.** Sequential cost-effectiveness analysis for catch-up vaccination, under base-case assumptions ^1^ with 5% or 15% catch-up campaign coverage by 2035 (2025 - 2050) | | | | | | | | |
| --- | --- | --- | --- | --- | --- | --- | --- | --- |
| **5% Catch-up Campaign Coverage** | | | | | | | | |
| **A. Female-only Vaccination** | | | | | | | | |
| Step | Intervention | Comparator | Total Cost (Million USD) ^2,3^ | Total QALY Decrements ^2^ | Δ Cost  (Million USD) | Δ QALYs Gained | **ICER ($/QALY)** |  |
| 1 | Scenario A | -- | 145,948·52 | 1,002,268 | -- | -- | -- |  |
| 2 | Scenario C | Scenario A | 145,838·65 | 989,942 | -109·87 | 12,326 | **Cost Saving** |  |
| **B. Sex-neutral Vaccination** | | | | | | | |  |
| Step | Intervention | Comparator | Total Cost (Million USD) ^2,3^ | Total QALY Decrements ^2^ | Δ Cost  (Million USD) | Δ QALYs Gained | **ICER ($/QALY)** |  |
| 1 | Scenario B | -- | 145,594·28 | 856,325 | -- | -- | -- |  |
| 2 | Scenario D | Scenario B | 145,564·83 | 848,614 | -29·45 | 7,711 | **Cost Saving** |  |
| **15% Catch-up Campaign Coverage** | | | | | | | |  |
| **A. Female-only Vaccination** | | | | | | | |  |
| Step | Intervention | Comparator | Total Cost (Million USD) ^2,3^ | Total QALY Decrements ^2^ | Δ Cost  (Million USD) | Δ QALYs Gained | **ICER ($/QALY)** |  |
| 1 | Scenario A | -- | 145,948·52 | 1,002,268 | -- | -- | -- |  |
| 2 | Scenario C | Scenario A | 145,646·22 | 967,693 | -302·30 | 34,575 | **Cost Saving** |  |
| **B. Sex-neutral Vaccination** | | | | | | | |  |
| Step | Intervention | Comparator | Total Cost (Million USD) ^2,3^ | Total QALY Decrements ^2^ | Δ Cost  (Million USD) | Δ QALYs Gained | **ICER ($/QALY)** |  |
| 1 | Scenario B | -- | 145,594·28 | 856,325 | -- | -- | -- |  |
| 2 | Scenario D | Scenario B | 145,518·48 | 834,752 | -75·81 | 21,573 | **Cost Saving** |  |
| ^1^ Base-case assumptions include Scenarios A-D: 50% coverage by 2035, 10-year duration of protection, and 70% VE; Scenarios C/D: 10% catch-up campaign coverage in 2035.  ^2^ Costs and QALYs are discounted at 3% annually.  ^3^ Costs are in 2025 USD  Abbreviations: USD, United States Dollar, QALY, quality-adjusted life years; VE, vaccine efficacy | | | | | | | | |

**Supplementary References**

1. Makhoul M, Ayoub HH, Awad SF, et al. Impact of a potential Chlamydia vaccine in the USA: mathematical modelling analyses. *BMJ Public Health*. 2024;2(1):e000345. doi:10.1136/BMJPH-2023-000345

2. Owusu-Edusei K, Chesson HW, Gift TL, Brunham RC, Bolan G. Cost-effectiveness of Chlamydia vaccination programs for young women. *Emerg Infect Dis*. 2015;21(6):960-968. doi:10.3201/EID2106.141270

3. Ditkowsky J, Rahman A, Hammerschlag MR, Kohlhoff S, Smith-Norowitz TA. Cost–Benefit Analysis of a Chlamydia trachomatis Vaccine Program in Adolescent Girls in the United States. *J Pediatric Infect Dis Soc*. 2018;7(4):296-302. doi:10.1093/JPIDS/PIX072

4. Gray RT, Beagley KW, Timms P, Wilson DP. Modelling the impact of potential vaccines on epidemics of sexually transmitted Chlamydia trachomatis infection. *Journal of Infectious Diseases*. 2009;199(11):1680-1688. doi:10.1086/598983

5. Omori R, Chemaitelly H, Althaus CL, Abu-Raddad LJ. Original article: Does infection with Chlamydia trachomatis induce long-lasting partial immunity? Insights from mathematical modelling. *Sex Transm Infect*. 2019;95(2):115. doi:10.1136/SEXTRANS-2018-053543

6. Rönn MM, Wolf EE, Chesson H, et al. The Use of Mathematical Models of Chlamydia Transmission to Address Public Health Policy Questions: A Systematic Review. *Sex Transm Dis*. 2017;44(5):278. doi:10.1097/OLQ.0000000000000598

7. The Lancet Child & Adolescent Health. Youth STIs: an epidemic fuelled by shame. *Lancet Child Adolesc Health*. 2022;6(6):353. doi:10.1016/S2352-4642(22)00128-6

8. Cheng Y, Zheng G, Song Z, Zhang G, Rao X, Zeng T. Trends in chlamydia prevalence in the United States, 2005–2016. *Sci Rep*. 2024;14(1):1-8. doi:10.1038/S41598-024-61818-5;SUBJMETA=4025,499,692;KWRD=RISK+FACTORS,UROLOGY

9. Greaves KE, Fairley CK, Engel JL, et al. Assortative Sexual Mixing by Age, Region of Birth, and Time of Arrival in Male-Female Partnerships in Melbourne, Australia. *Sex Transm Dis*. 2023;50(5):288-291. doi:10.1097/OLQ.0000000000001772

10. Malagón T, Burchell A, El-Zein M, Tellier PP, Coutlée F, Franco EL. Assortativity and mixing by sexual behaviors and sociodemographic characteristics in young adult heterosexual dating partnerships. *Sex Transm Dis*. 2017;44(6):329. doi:10.1097/OLQ.0000000000000612

11. Kreisel KM, Weston EJ, St. Cyr SB, Spicknall IH. Estimates of the Prevalence and Incidence of Chlamydia and Gonorrhea Among US Men and Women, 2018. *Sex Transm Dis*. 2021;48(4):222. doi:10.1097/OLQ.0000000000001382

12. United Nations Department of Economic and Social Affairs Population Division. 2024 Revision of World Population Prospects. 2024. Accessed February 12, 2025. https://population.un.org/wpp/

13. Centers for Disease Control and Prevention. National Center for HIV, Viral Hepatitis, STD, and Tuberculosis Prevention’s AtlasPlus Tool. Accessed December 9, 2024. https://www.cdc.gov/nchhstp/about/atlasplus.html

14. Learner ER, Powers KA, Torrone EA, Pence BW, Fine JP, Miller WC. The influence of screening, misclassification, and reporting biases on reported chlamydia case rates among young women in the US, 2000 through 2017. *Sex Transm Dis*. 2020;47(6):369. doi:10.1097/OLQ.0000000000001157

15. Kreisel KM, Spicknall IH, Gargano JW, et al. Sexually Transmitted Infections Among US Women and Men: Prevalence and Incidence Estimates, 2018. *Sex Transm Dis*. 2021;48(4):208. doi:10.1097/OLQ.0000000000001355

16. Tao G, Hoover KW, Kent CK. Chlamydia testing patterns for commercially insured women, 2008. *Am J Prev Med*. 2012;42(4):337-341. doi:10.1016/J.AMEPRE.2011.11.013

17. Emory AMIS - American Men’s Internet Survey. Accessed January 11, 2025. https://emoryamis.org/

18. Davidson KW, Barry MJ, Mangione CM, et al. Screening for Chlamydia and Gonorrhea: US Preventive Services Task Force Recommendation Statement. *JAMA*. 2021;326(10):949-956. doi:10.1001/JAMA.2021.14081

19. Tao G, Hufstetler K, He L, et al. Underlying Reasons for Primary Care Visits Where Chlamydia Testing Was Performed in the United States, 2019 to 2022. *Sex Transm Dis*. 2024;51(7):456-459. doi:10.1097/OLQ.0000000000001976

20. Brase PR, Dombrowski JC, Berzkalns A, Manhart LE, Golden MR, Khosropour CM. Trends in Chlamydia trachomatis Treatment Prescribing Practices in King County, Washington, 2010-2018. *Sex Transm Dis*. 2024;51(5):305-312. doi:10.1097/OLQ.0000000000001940

21. Berman SM, Satterwhite CL. A Paradox: Overscreening of older women for chlamydia while too few younger women are being tested. *Sex Transm Dis*. 2011;38(2):130-132. doi:10.1097/OLQ.0B013E3182027E00

22. Bennett BW, DuBose S, Huang YLA, et al. Population Percentage and Population Size of Men Who Have Sex With Men in the United States, 2017-2021: Meta-Analysis of 5 Population-Based Surveys. *JMIR Public Health Surveill*. 2024;10. doi:10.2196/56643

23. Mosher WD, Chandra A, Jones J. Sexual Behavior and Selected Health Measures: Men and Women 15-44 Years of Age, United States, 2002. *Number*. 2002;362.

24. NSFG - National Survey of Family Growth. Accessed January 11, 2025. https://www.cdc.gov/nchs/nsfg/index.htm

25. Torrone EA, Geisler WM, Gift TL, Weinstock HS. Chlamydia trachomatis Infection Among Women 26 to 39 Years of Age in the United States, 1999 to 2010. *Sex Transm Dis*. 2013;40(4):335. doi:10.1097/OLQ.0B013E31827CD60D

26. HEDIS - NCQA. Accessed January 11, 2025. https://www.ncqa.org/hedis/

27. Weiss KM, Jones JS, Anderson EJ, et al. Optimizing Coverage vs Frequency for Sexually Transmitted Infection Screening of Men Who Have Sex With Men. *Open Forum Infect Dis*. 2019;6(10). doi:10.1093/OFID/OFZ405

28. Morris E, Kanny D, Teplinskaya A, et al. HIV Surveillance Special Report: HIV Infection Risk, Prevention, and Testing Behaviors Among Men Who Have Sex With Men—National HIV Behavioral Surveillance, 19 U.S. Cities, 2023. Preprint posted online 2024. Accessed January 11, 2025. https://stacks.cdc.gov/view/cdc/162349

29. Berry SA, Ghanem KG, Mathews WC, et al. GONORRHEA AND CHLAMYDIA TESTING INCREASING BUT STILL LAGGING IN HIV CLINICS IN THE UNITED STATES. *J Acquir Immune Defic Syndr*. 2015;70(3):275. doi:10.1097/QAI.0000000000000711

30. Hoots BE, Torrone EA, Bernstein KT, Paz-Bailey G. Self-Reported Chlamydia and Gonorrhea Testing and Diagnosis Among Men Who Have Sex With Men—20 US Cities, 2011 and 2014. *Sex Transm Dis*. 2018;45(7):469. doi:10.1097/OLQ.0000000000000786

31. European Centre for Disease Prevention and Control. Factsheet About Chlamydia. Accessed January 9, 2025. https://www.ecdc.europa.eu/en/chlamydia/facts

32. Brunham RC, Pourbohloul B, Mak S, White R, Rekart ML. The unexpected impact of a Chlamydia trachomatis infection control program on susceptibility to reinfection. *Journal of Infectious Diseases*. 2005;192(10):1836-1844. doi:10.1086/497341,

33. McWhirter L, Lou Y, Reingold S, et al. Rates of appropriate treatment and follow-up testing after a gonorrhea and/or chlamydia infection in an urban network of federally qualified health center systems. *Sex Transm Dis*. 2022;49(5):319. doi:10.1097/OLQ.0000000000001600

34. Dukers-Muijrers NHTM, Wolffs PFG, De Vries H, et al. Treatment Effectiveness of Azithromycin and Doxycycline in Uncomplicated Rectal and Vaginal Chlamydia trachomatis Infections in Women: A Multicenter Observational Study (FemCure). *Clin Infect Dis*. 2019;69(11):1946. doi:10.1093/CID/CIZ050

35. Lowe P, O’Loughlin P, Evans K, White M, Bartley PB, Vohra R. Comparison of the Gen-Probe APTIMA Combo 2 Assay to the AMPLICOR CT/NG Assay for Detection of Chlamydia trachomatis and Neisseria gonorrhoeae in Urine Samples from Australian Men and Women. *J Clin Microbiol*. 2006;44(7):2619. doi:10.1128/JCM.00476-06

36. Gaydos CA, Quinn TC, Willis D, et al. Performance of the APTIMA Combo 2 Assay for Detection of Chlamydia trachomatis and Neisseria gonorrhoeae in Female Urine and Endocervical Swab Specimens. *J Clin Microbiol*. 2003;41(1):304. doi:10.1128/JCM.41.1.304-309.2003

37. Van Pol B Der, Liesenfeld O, Williams JA, et al. Performance of the cobas CT/NG Test Compared to the Aptima AC2 and Viper CTQ/GCQ Assays for Detection of Chlamydia trachomatis and Neisseria gonorrhoeae. *J Clin Microbiol*. 2012;50(7):2244. doi:10.1128/JCM.06481-11

38. Owusu-Edusei K, Gift TL, Chesson HW, Kent CK. Investigating the potential public health benefit of jail-based screening and treatment programs for chlamydia. *Am J Epidemiol*. 2013;177(5):463-473. doi:10.1093/AJE/KWS240,

39. Brunham RC, Gottlieb SL, Paavonen J. Pelvic Inflammatory Disease. Campion EW, ed. *New England Journal of Medicine*. 2015;372(21):2039-2048. doi:10.1056/NEJMRA1411426

40. *Epididymitis - STI Treatment Guidelines*. 2021. Accessed May 10, 2025. https://www.cdc.gov/std/treatment-guidelines/epididymitis.htm

41. Rogers B, Tao J, Murphy M, Chan PA. The COVID-19 pandemic and sexually transmitted infections: Where do we go from here? *Sex Transm Dis*. 2021;48(7):e94. doi:10.1097/OLQ.0000000000001445

42. Gavin HP. The Levenberg-Marquardt algorithm for nonlinear least squares curve-fitting problems. Published online 2024.

43. Patel CG, Trivedi S, Tao G. The Proportion of Young Women Tested for Chlamydia Who Had Urogenital Symptoms in Physician Offices. *Sex Transm Dis*. 2018;45(9):e72. doi:10.1097/OLQ.0000000000000858

44. Farley TA, Cohen DA, Elkins W. Asymptomatic sexually transmitted diseases: the case for screening. *Prev Med (Baltim)*. 2003;36(4):502-509. doi:10.1016/S0091-7435(02)00058-0

45. *Infertility & STDs*. 2024. Accessed May 10, 2025. https://archive.cdc.gov/www_cdc_gov/std/infertility/default.htm

46. Herzog SA, Althaus CL, Heijne JCM, et al. Timing of progression from Chlamydia trachomatis infection to pelvic inflammatory disease: A mathematical modelling study. *BMC Infect Dis*. 2012;12(1):1-9. doi:10.1186/1471-2334-12-187/FIGURES/3

47. Sivaraj V, Ahamed A, Artykov R, Menon-Johansson A. Epididymitis and its aetiologies in a central London sexual health clinic. *Int J STD AIDS*. 2021;32(1):96-99. doi:10.1177/0956462420963879

48. Gift TL, Gaydos CA, Kent CK, et al. The program cost and cost-effectiveness of screening men for Chlamydia to prevent pelvic inflammatory disease in women. *Sex Transm Dis*. 2008;35(11 Suppl). doi:10.1097/OLQ.0B013E31818B64AC

49. Price MJ, Ades A, Soldan K, et al. The natural history of Chlamydia trachomatis infection in women: a multi-parameter evidence synthesis. *Health Technol Assess (Rockv)*. 2016;20(22). doi:10.3310/hta20220

50. Lewis J, Price MJ, Horner PJ, White PJ. Genital Chlamydia trachomatis Infections Clear More Slowly in Men Than Women, but Are Less Likely to Become Established. *J Infect Dis*. 2017;216(2):237. doi:10.1093/INFDIS/JIX283

51. He L, Patel C, Tao G. National Chlamydia Screening Rate in Young Sexually Active Women Using HEDIS Measures in the United States, 2011 to 2020. *Sex Transm Dis*. 2023;50(7):415-419. doi:10.1097/OLQ.0000000000001809

52. Nelson SJ, Hughes JP, Foxman B, et al. Age- and gender-specific estimates of partnership formation and dissolution rates in the Seattle Sex Survey. *Ann Epidemiol*. 2010;20(4):308. doi:10.1016/J.ANNEPIDEM.2009.11.003

53. Eaton JW, Hallett TB, Garnett GP. Concurrent Sexual Partnerships and Primary HIV Infection: A Critical Interaction. *AIDS Behav*. 2011;15(4):687. doi:10.1007/S10461-010-9787-8

54. Althaus CL, Heijne JCM, Low N. Towards more robust estimates of the transmissibility of chlamydia trachomatis. *Sex Transm Dis*. 2012;39(5):402-404. doi:10.1097/OLQ.0B013E318248A550,

55. Ayoub HH, Chemaitelly H, Abu-Raddad LJ. Characterizing the transitioning epidemiology of herpes simplex virus type 1 in the USA: model-based predictions. *BMC Med*. 2019;17(1):57. doi:10.1186/S12916-019-1285-X

56. National Center for Health Statistics. *National Health and Nutrition Examination Survey Questionnaire*. Accessed January 31, 2025. https://wwwn.cdc.gov/nchs/nhanes/default.aspx

57. Stoecker C, Monnette A, Qu Z, Schmidt N, Craig-Kuhn MC, Kissinger PJ. Cost-effectiveness of Check It: A Novel Community-Based Chlamydia Screening and Expedited Treatment Program for Young Black Men. *Clin Infect Dis*. 2022;74(12):2166. doi:10.1093/CID/CIAB818

58. Rönn MM, Li Y, Gift TL, et al. Costs, Health Benefits, and Cost-Effectiveness of Chlamydia Screening and Partner Notification in the United States, 2000–2019: A Mathematical Modeling Analysis. *Sex Transm Dis*. 2023;50(6):351. doi:10.1097/OLQ.0000000000001786

59. Li Y, You S, Lee K, et al. The Estimated Lifetime Quality-Adjusted Life-Years Lost Due to Chlamydia, Gonorrhea, and Trichomoniasis in the United States in 2018. *J Infect Dis*. 2023;227(8):1007. doi:10.1093/INFDIS/JIAD047

60. Chesson H, Spicknall IH, Kreisel KM, Gift TL. Estimates of the Lifetime Productivity Costs of Chlamydia, Gonorrhea, and Syphilis in the United States. *Sex Transm Dis*. 2024;51(10):635. doi:10.1097/OLQ.0000000000001973

61. Goldie SJ, Kohli M, Grima D, et al. Projected Clinical Benefits and Cost-effectiveness of a Human Papillomavirus 16/18 Vaccine. *JNCI: Journal of the National Cancer Institute*. 2004;96(8):604-615. doi:10.1093/JNCI/DJH104

62. Kim JJ, Ortendahl J, Goldie SJ. Cost-Effectiveness of HPV Vaccination and Cervical Cancer Screening in Women over Age 30 in the United States. *Ann Intern Med*. 2009;151(8):538. doi:10.7326/0003-4819-151-8-200910200-00007

63. Chesson HW, Meites E, Ekwueme DU, Saraiya M, Markowitz LE. Cost-effectiveness of HPV vaccination for adults through age 45 years in the United States: Estimates from a simplified transmission model. *Vaccine*. 2020;38(50):8032. doi:10.1016/J.VACCINE.2020.10.019

64. Prosser LA, O’Brien MA, Molinari NAM, et al. Non-traditional settings for influenza vaccination of adults: Costs and cost effectiveness. *Pharmacoeconomics*. 2008;26(2):163-178. doi:10.2165/00019053-200826020-00006/METRICS

65. Centers for Disease Control and Prevention. Current CDC Vaccine Price List. U.S. Department of Health and Human Services. 2025. Accessed May 23, 2025. https://www.cdc.gov/vaccines-for-children/php/awardees/current-cdc-vaccine-price-list.html

66. Kumar S, Chesson H, Gift TL. Estimating the Direct Medical Costs and Productivity Loss of Outpatient Chlamydia and Gonorrhea Treatment. *Sex Transm Dis*. 2021;48(2):e18. doi:10.1097/OLQ.0000000000001240

67. Eckman MH, Reed JL, Trent M, Goyal MK. Cost-effectiveness of Sexually Transmitted Infection Screening for Adolescents and Young Adults in the Pediatric Emergency Department. *JAMA Pediatr*. 2021;175(1):1. doi:10.1001/JAMAPEDIATRICS.2020.3571

68. Dean LT, Montgomery MC, Raifman J, et al. The Affordability of Providing Sexually Transmitted Disease Services at a Safety-net Clinic. *Am J Prev Med*. 2018;54(4):552. doi:10.1016/J.AMEPRE.2017.12.016

69. Kumar S, Chesson HW, Spicknall IH, Kreisel KM, Gift TL. The Estimated Lifetime Medical Cost of Chlamydia, Gonorrhea, and Trichomoniasis in the United States, 2018. *Sex Transm Dis*. 2021;48(4):238. doi:10.1097/OLQ.0000000000001357

70. Grosse SD. Assessing cost-effectiveness in healthcare: History of the $50,000 per QALY threshold. *Expert Rev Pharmacoecon Outcomes Res*. 2008;8(2):165-178. doi:10.1586/14737167.8.2.165,

71. Neumann PJ, Kim DD. Cost-effectiveness Thresholds Used by Study Authors, 1990-2021. *JAMA*. 2023;329(15):1312-1314. doi:10.1001/JAMA.2023.1792
